# Novel long-chain compounds with both immunomodulatory and MenA inhibitory activities against *Staphylococcus aureus* and its biofilm

**DOI:** 10.1038/srep40077

**Published:** 2017-01-10

**Authors:** Seoung-ryoung Choi, Joel Frandsen, Prabagaran Narayanasamy

**Affiliations:** 1 Department of Pathology and Microbiology, College of Medicine, University of Nebraska Medical Center, Omaha, Nebraska 68198, USA.

## Abstract

Menaquinone (MK) biosynthesis pathway is a potential target for evaluating antimicrobials in gram-positive bacteria. Here, 1,4-dihydroxy-2-naphthoate prenyltransferase (MenA) was targeted to reduce methicillin-resistant *Staphylococcus aureus* (MRSA) growth. MenA inhibiting, long chain-based compounds were designed, synthesized and evaluated against MRSA and menaquinone utilizing bacteria in aerobic conditions. The results showed that these bacteria were susceptible to most of the compounds. Menaquinone (MK-4) supplementation rescued MRSA growth, suggesting these compounds inhibit MK biosynthesis. **3a** and **7c** exhibited promising inhibitory activities with MICs ranging 1–8 μg/mL against MRSA strains. The compounds did not facilitate small colony variant formation. These compounds also inhibited the biofilm growth by MRSA at high concentration. Compounds **3a**, **6b** and **7c** displayed a promising extracellular bactericidal activity against MRSA at concentrations equal to and four-fold less than their respective MICs. We also observed cytokines released from THP-1 macrophages treated with compounds **3a**, **6b** and **7c** and found decreases in TNF-α and IL-6 release and increase in IL-1β. These data provide evidence that MenA inhibitors act as TNF-α and IL-6 inhibitors, raising the potential for development and application of these compounds as potential immunomodulatory agents.

Lipid-soluble vitamin K was discovered in 1929^1^ as an essential nutrient for anti-hemorrhage. Its structure and chemical nature were previously reported^2^. It is biosynthesized by many bacteria including *Escherichia coli, Mycobacterium tuberculosis* and *Staphylococcus aureus*. Vitamin K can be divided into natural and synthetic forms; Vitamin K_1_ (phylloquinone) and K_2_ (menaquinone, MK-4) are natural forms stored in the fat tissue and liver. Several homologues of vitamin K_2_ (MK-7 to MK-15) are produced by bacteria by changing the length of the isoprenoid chain. Synthetic forms of K_3_ (menaphthone or menadione), K_4_, and K_5_ are used as a nutritional supplement or fungal growth inhibitor. In humans, phylloquionone is supplemented from food while menaquinones (MKs) are produced by conversion of phylloquinone by intestinal bacteria. Humans do not possess a MK biosynthesis pathway, and MK is not utilized in the electron transport chain. Because of this, enzymes involved in either function have recently received attention as potential drug targets to treat Gram-positive pathogens.

Ubiquinone and MK play important roles in the prokaryote electron transport chain by transferring electrons between membrane bound protein complexes^3^. In Gram-positive bacteria, MK biosynthesis is essential for survival because MK is the only electron carrier in both aerobic and anaerobic conditions. MKs are biosynthesized via two independent classical and futalosin pathways^4^,^5^. Both pathways utilize chorismate as substrate - derived from phosphoenolpyruvate and D-erythrose-4-phosphate by the shikimate pathway - but do not share other similarities. As shown in Fig. 1, 1,4-dihydroxy-2-naphthoate (DHNA) is synthesized from chorismate by MenFDHCEB enzymes involved in the classical MK pathway. Prenylation by MenA followed by methylation by methyltransferse (MenG) convert the 2-naphthoate to MK. The futalosin pathway uses MqnABCD enzymes to synthesize 1,4-dihydroxy-6-naphthoate which is converted to MK *via* a series of reactions catalyzed by unknown enzymes.

Inhibitors of enzymes involved in MK biosynthesis demonstrate that targeting these enzymes may lead to therapeutics for treatments of infections by Gram-positive bacteria including *Bacillus subtilis, M. tuberculosis*, and *S. aureus*^6^,^7^. 4-oxo-4-phenylbutanoate analogues were developed as MenB inhibitors active against MRSA and both replicating and nonreplicating *M. tuberculosis*^8^,^9^. Mechanism-based inhibitors of an acyl-CoA synthetase (MenE) have been reported with low micromolar IC_50_ values^10^,^11^,^12^. Inhibitors of MenD^13^ and MenC^14^ were also discovered, validating the classical menaquinone pathway as a potential target for the development of antibacterial drugs. Here, we report the synthesis and evaluation of potential MenA inhibiting antimicrobials and immunomodulatory compounds.

## Results and Discussion

**Figure 1:**
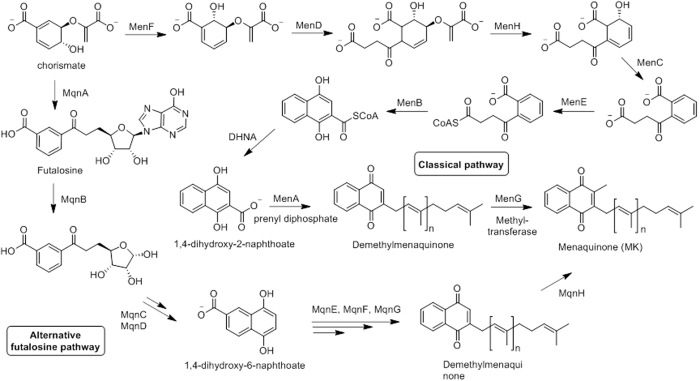
Menaquinone biosynthesis pathways. Classical menaquinone and alternative futalosine pathways from chorismate. MqnA: futalosine synthase, MqnB: futalosine hydrolase, MqnC: dehypoxanthinyl futalosine cyclase, MqnD: 1,4-dihydroxy-6-naphthoate synthase. MenF: isochorismate synthase, MenC: o-succinylbenzoate synthase. MenD: 2-succinyl-5-enolpyruvyl-6-hydroxy-3-cyclohexene-1-carboxylate synthase. MenH: (1R, 6R)-6-hydroxy-2-succinylcyclohexa-2, 4-diene-1-carboxylate synthase.

**Figure 2:**
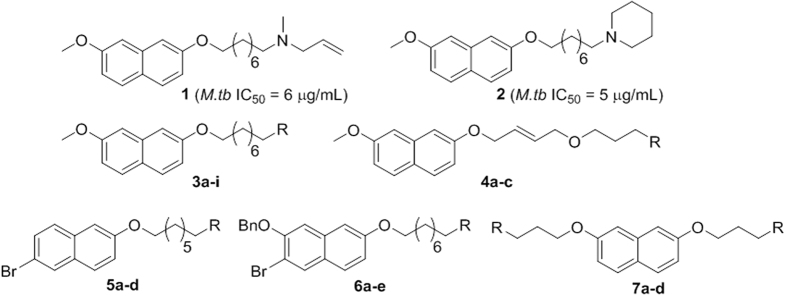
Synthesis of Long chain MenA inhibitors.

A transmembrane protein 1,4-dihydroxy-2-naphthoate prenyltransferase (MenA) catalyzes formation of demethylmenaquinone (DMMK) via consecutive decarboxylation and prenylation of DHNA (Fig. 1). MenA is a promising target for Gram-positive bacteria, especially MRSA and drug-resistant *M.tb*. Benzophenone-based analogues were synthesized and identified as MenA inhibitors against MRSA and nonreplicating and replicating *M.tb*^15^,^16^,^17^,^18^. However, benzoquinone analogues are known to be toxic due to the presence of a benzophenone moiety^15^,^19^,^20^,^21^. Previously, we reported that MenA inhibitors possessing a simple, chemical structural backbone of 7-methoxy naphthalene are highly active against Gram-positive bacteria with low micromolar MIC and IC_50_ values (compound **1** and **2** in Fig. 2)^6^. Here, we synthesized novel compounds to explore structure-activity relationships (compounds **3** to **7** in Fig. 2), determined effect on cytokine expression by macrophages and reduced MRSA growth and its biofilm.

### Antimicrobial activity of naphthalene derivatives

MICs were determined against methicillin-resistant *S. aureus* strains and Gram-positive and negative bacteria. We previously reported that compound **1** and **2** exhibited inhibition of both *M.tb* and MenA enzyme activity with IC_50_ values of 6 and 5 μg/mL, respectively^6^. We synthesized long chain compounds with different functionalities to improve activity. Various active groups were introduced at the terminal part of carbon chain (tail group) to find a lead structure and evaluate its structure-activity relationship against MRSA. As expected, all the synthesized compounds showed no inhibition against Gram-negative *P. aeruginosa,* whereas most of the compounds inhibited growth of Gram-positive bacteria including seven MRSA strains, MSSA, EF and two species of *Mycobacterium*. Compounds **3a**, **3b**, **3c** and **7c** showed the most potent inhibitory activities against all Gram-positive bacteria with MIC values ranging from 0.5 to 16 μg/mL (Table 1). These compounds share a phenylethylamine group that plays an important role in growth inhibition of Gram-positive bacteria. Although the compounds are very active, replacing the fluorine in **3a** with other halogens did not significantly improve the MIC, and introduction of bicyclic or tricyclic ring in the terminal end reduced the activity (**3d-3f**). Replacing the amine group with oxazolidinone also did not improve the activity (**3g-3h**) but the linezolid control was active. Similarly, the presence of fluorine in the piperidine group did not increase inhibitory activity (**3i**). Introduction of oxygen in the carbon chain had no effect on inhibitory activity (**4a** and **4b**), but substitution of 4-fluorophenylethylamine at the tail end (**4c**) maintained activity. Modification of the 7-methoxynaphthalene group by replacing the methoxy group with bromine did not improve inhibitory activity of 4-fluorophenylethylamine compounds (**5a-c**). However, bromine substituted naphthalene increased activity for piperidine tail end substitution (**5d)**. Likewise, replacement of methoxy group with benzyloxy group did not affect growth inhibition for piperidine terminal end but significantly reduced the activity of 4-fluorophenylethylamine terminal end (**6a-e**). These data show that a bulkier substitution is needed at any one of the terminal groups. Interestingly, it was found that **7c** was the most potent inhibitor against all tested Gram-positive bacteria showing MIC between 1–6 μg/mL. Dual substitution of 4-fluorophenylethylamine increased the activity, but dual substitution of piperidine or allyl amine decreased the activity.

### MRSA growth rescued by menaquinone (MK-4)

**Figure 3:**
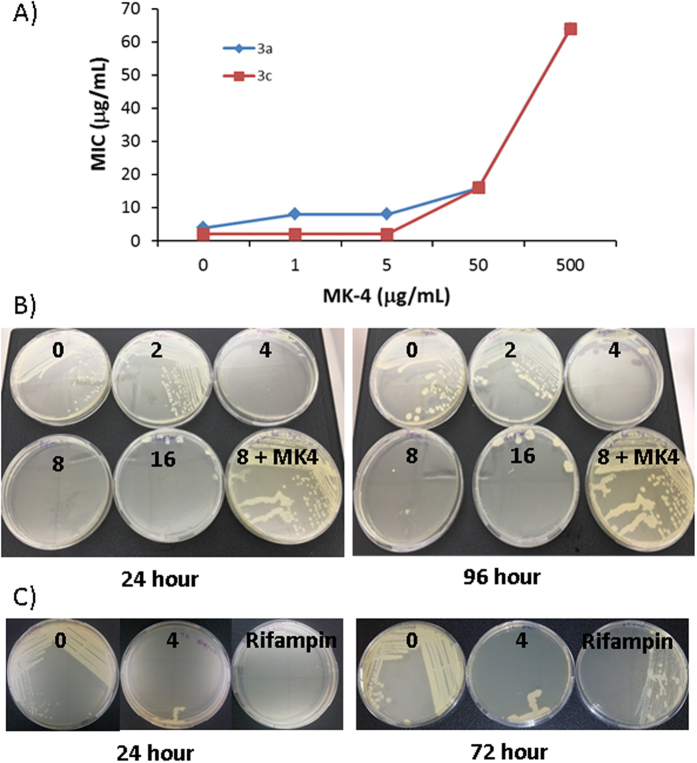
Menaquinone rescue (**A**) and colony morphology (**B**). (**A**) MRSA (USA 700 for **3a**, USA 200 for **3c**) was treated with various concentrations of menaquinone (MK-4) in the presence of inhibitor. (**B**) MRSA (USA 200) was incubated on TSA containing various concentrations of **3a** (0, 2, 4, 8, 16 μg/mL and 8 μg/mL +100 μg/mL MK4) up to 96 h. (**C**) MRSA (USA 200) was incubated on TSA containing **7c** and rifampin (positive control) up to 72 h.

Rescue experiments were performed via resazurin reduction assay to investigate the effect of menaquinone in the presence of various concentrations (0.5–64 μg/mL) of inhibitor **3a** or **3c** against two MRSA strains USA 700 or 200, respectively. MIC of **3a** was 4 μg/mL against USA 700 and **3c** was 2 μg/mL against USA 200 (Table 1). As shown in Fig. 3 and Figure S1, MIC values for both inhibitors increased in a concentration-dependent manner by addition of MK-4. MICs of both inhibitors moved to 64 μg/mL in the presence of 0.5 mg/mL MK-4. Similarly, when the media is supplemented with 0.05 mg/mL MK-4, MICs increased to 16 μg/mL. Thus, the rescue experiments suggested that menaquinone biosynthesis is inhibited by **3a** and **3c**.

### Colony morphology

A slow growing auxotrophic bacterial subpopulation resulting from mutations in metabolic genes has very distinctive phenotype compared to its parent wild-type strain. Gene mutations, caused by environmental factors including treatment of antibiotics, metabolic defects and genetic defects, lead to slow growing small colony variants (SCV) of bacteria that are related to electron transport defective strains and thymidylate biosynthesis^22^,^23^. Among the clinical bacteria forming SCV, *Pseudomonas aeruginosa, Enterococcus faecalis*^24^,^25^, and *S. aureus* SCV are a more prominent problem due to their association with intracellular persistence and linked chronic recurrent and antibiotic-resistant infections. Patients with cystic fibrosis, a genetic disorder caused by a mutation in CFTR gene, are especially in danger of higher probability of *S. aureus* SCV infection which can persist intracellularly in the host^26^. Development of inhibitors that do not induce SCV are urgent and important to treat *S. aureus*. We tested **3a**, to analyze induction of SCV by growing MRSA on TSA. MRSA (USA200) was grown on TSA containing 1×, 2×, 4×, or 8× MIC of **3a** up to 96 h at 37 °C (Figs 3 and S2). No colonies were observed on agar plates that contained more than 2× MIC of **3a** after 24 h. MK-4 (100 μg/mL) supplemented agar plate containing 8 μg/mL of **3a** showed wild-type colonies, indicating that the inhibitory activity was hampered by MK-4. However, no growth of SCV was observed even after incubation of TSA with 4–16 μg/mL up to 96 h, suggesting **3a** did not induce SCV. MRSA (USA200) was also grown on TSA containing **7c** and rifampin (positive control). At 72 h, rifampin showed growth of SCV and confirming that **7c** did not induce SCV (Fig. 3C).

### Antimicrobial activity against MRSA biofilms

**Figure 4:**
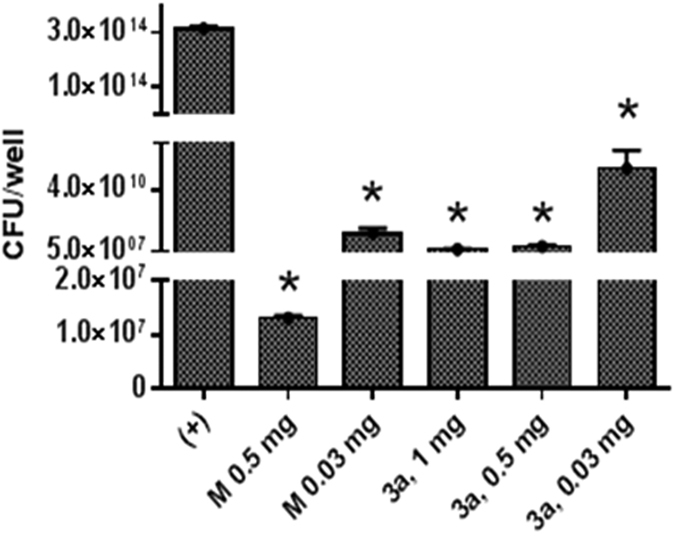
Antibacterial activity against MRSA biofilm. MRSA (USA700) biofilms were treated with **3a** or minocycline for 24 h and serially diluted and plated on TSA plates to determine CFU. Data represents the mean ± SEM of triplicate (n = 3). M: minocycline, Statistical differences were determined using Student’s *t* test: *p < 0.001 compared to (+) positive control.

We next investigated if inhibitor **3a** exhibits inhibitory activity against MRSA biofilms. Biofilms by Gram-positive bacteria are known to be more resistant to many antibiotics and the required concentration to eradicate biofilm is much higher than MIC^27^. Minocycline, a tetracycline antibiotic used for skin infection, was used as a control for biofilm eradication. It showed significant decrease in MRSA biofilm growth at concentrations of 0.5 and 0.03 mg/mL compared to the positive control (Fig. 4). Concurrently, Compound **3a** showed 10^4^, 10^6^ and 10^7^ fold decrease in MRSA growth from the treated biofilm at 0.03, 0.5 and 1 mg/mL, respectively. Thereby **3a** was determined to be effective in reducing biofilm growth.

### Growth Inhibition of extracellular MRSA (USA700) in human macrophages by inhibitor

**Figure 5:**
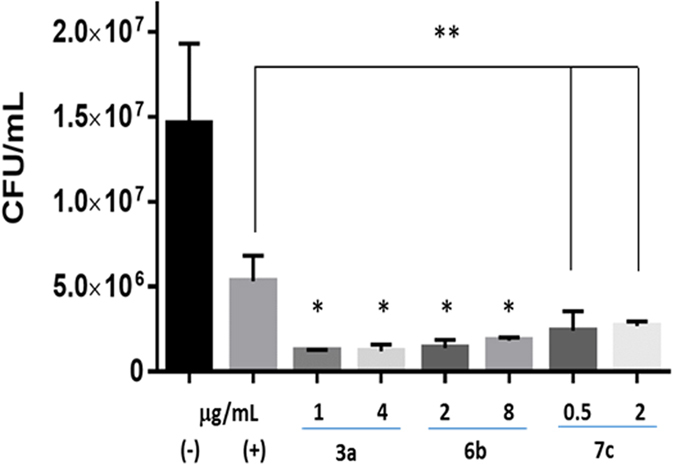
Macrophage-mediated inhibition of MRSA growth with inhibitors. Human monocytes were differentiated into macrophages (MDM) in the presence of MCSF. MDMs were incubated with MRSA (USA 700) in the presence of inhibitor for 24 h at 37 °C. Media were serially diluted and plated on TSA to determine CFU. (−): No MDM and No inhibitor, (+): No inhibitor. Data represents the mean ± SEM of triplicate (n = 3). Statistical differences were determined using Student’s *t* test: *p < 0.002, **p < 0.01 compared to (+) positive control.

We next investigated the bactericidal activities of inhibitors in human macrophages incubated with MRSA. Monocyte-derived macrophages were obtained by incubating monocytes in the presence of MCSF in DMEM media containing 10% human serum. Adherent MDMs were incubated with MRSA (1 × 10^6^ CFU/mL) and treated with inhibitors at concentrations of MIC and MIC/4 for 24 h. Extracellular MRSA surviving in media was determined by counting colonies. As seen in control experiments in Fig. 5, a 3 fold decrease of extracellular antibacterial activity by MDMs was observed in culture media, indicating that MRSA was phagocytized and killed by MDMs. Our inhibitors were then tested in the presence or absence of MDMs. Antibacterial activities of inhibitors (**3a**, **6b**, **7c**) tested were increased by 6–12 fold compared to activities without MDMs and by 1.5 – 3-fold compared to activities with MDMs. This result suggests that these inhibitors prevented extracellular bacterial growth at their MIC and MIC/4, but use of a concentration greater than the MIC is required for complete bactericidal activity. However, we did not observe concentration-dependent antibacterial activities when MDMs were treated with MRSA and inhibitors at different concentrations.

### Cytokine secretion by THP-1 macrophages treated with MenA inhibitors

**Figure 6:**
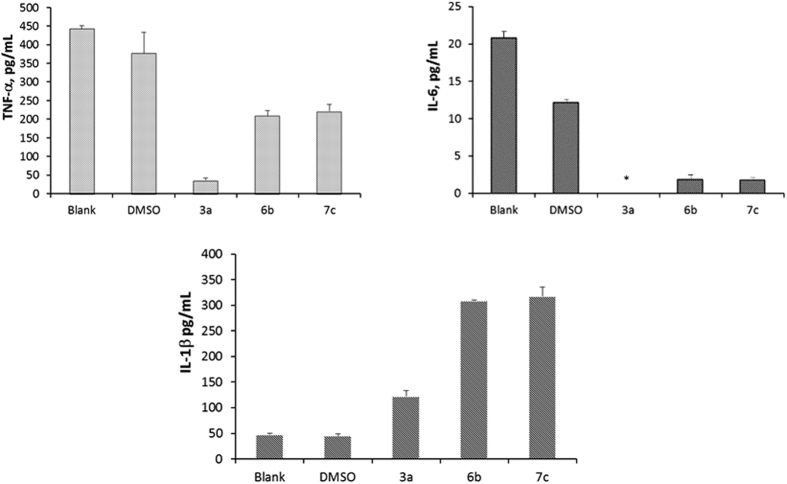
TNF-α, IL-6 and IL-1β released from macrophages treated with long-chain inhibitors. THP-1 macrophages were treated with **3a, 6b** or **7c** at concentrations equal to their MICs for 24 h at 37 °C. Culture supernatants were analyzed for cytokines. Data represents the mean ± SEM of triplicate samples (n = 3). Blank: No inhibitor was added. DMSO (<0.05%), *out of range below the detection limit.

Since MenA inhibitors enhanced the bactericidal activity of macrophages, we next examined whether macrophages treated with inhibitors display an altered cytokine profile. The cytokines released from THP-1 macrophages were assessed from culture supernatants using a Luminex system. Among 10 cytokines analyzed, IL-8 was most abundant in ~25 ng/ml (Figure S2) but there was no significant difference in IL-8 expression in the presence or absence of inhibitors. Interestingly, we found that amounts of TNF-α and IL-6 released from THP-1 macrophages were decreased in the presence of inhibitors **3a**, **6b** and **7c** whereas these inhibitors stimulated the release of IL-1β at concentrations equal to their MICs (Fig. 6). These intriguing results suggest that this class of small molecules, in addition to antibiotic activity, possesses potential to be developed as TNF-α and IL-6 suppressors for use as immunomodulatory therapy for the treatment of inflammatory diseases. In addition, IL-1β was also regulated by the same MenA inhibitors.

The MenA inhibitors are composed of three main groups: naphthol, carbon chain linker, and amine tail group. We explored this class of inhibitor by modifying these three main backbone structures to find a more potent lead structure against *S. aureus*. We found that **3a-c** and **5a** have potent activity against MRSA strains, MSSA and other bacteria with MIC values ranging 1–16 μg/mL. As expected, all compounds showed no activity against Gram-negative *P. aeruginosa*. Interestingly, **7c** showed very low MIC values against MK utilizing bacteria (Table 1), suggesting it is a potential lead compound for future optimization. MK rescue experiment confirmed the involvement of the MK biosynthetic pathway in reducing MRSA growth. MRSA strains treated with **3a** or **7c** were rescued by MK4, supporting disruption of electron transfer chain by MenA inhibitor. IC_50_ analysis also confirmed that MenA is the target for compounds. **3a** was found to inhibit MRSA biofilm growth, which is defined as a sessile microbial community. *S. aureus* biofilms colonize medical implants and host tissue and contribute to the persistence of chronic infections. The cells are protected in an extracellular polymeric matrix in which their physiologies are altered with respect to gene expression and protein production. Thus, biofilm formation reduces susceptibility to antimicrobials and requires use of larger quantity of antimicrobials. As expected, minocycline as a reference did not completely inhibit MRSA (USA 700) biofilm at 8300 × MIC (broth MIC < 0.06 μg/mL, Table 1), showing 10^6^-fold reduction compared to the positive control. Likewise, treatment of MRSA biofilm with **3a** (250 × MIC) inhibited the MRSA growth by 10^5^ fold. The results suggest that MenA inhibitor **3a** is highly effective in preventing MRSA growth.

Development of antibiotics that are active against both extracellular and intracellular pathogens is important to eradicate infectious bacteria. MenA inhibitors in the presence of macrophages showed promising activities against extracellular MRSA. We observed macrophage stimulation by MenA inhibitors alters the cytokine-release profile, causing a reduction of TNF-α and IL-6, and an increase of IL-1β. These three cytokines play a vital role in immune defense by responding to pathogens and inflammatory states. They have also been implicated as novel immunomodulatory therapeutic targets.

The pro- and anti-inflammatory IL-6 participates in the host immune defense. IL-6 binding to either form of its receptors (transmembrane and soluble IL-6 receptors) triggers biological signal transductions, affecting various cell populations^28^. However, dysregulation of IL-6 production is a major causative factor of autoimmune and chronic inflammatory diseases. There are various drugs that block IL-6 signaling pathways by targeting IL-6, IL-6 receptors or gp130 in development stage or market. For example, monoclonal antibody (tocilizumab) to IL-6R has been approved and used for treatment of rheumatoid arthritis, indicating that IL-6 blockade is a novel strategy for inflammatory diseases. Since macrophages are one of major sources of IL-6, reducing or blocking IL-6 release from macrophages by an anti-IL-6 reagent is an alternative strategy in development of therapeutic agent. As shown in Fig. 6, it is observed that treatment of THP-1 macrophages with MenA inhibitors (**3a**, **6b**, or **7c**) significantly reduced IL-6 release, providing a rational strategy for the development of immunomodulatory agent and prompting us to investigate mechanism of action of MenA inhibitor that affects IL-6 release of macrophage.

Several monoclonal antibody drugs such as infliximab (Remicade), etanercept (Enbrel), adalimumab (Humira), certolizumab pegol (Cimzia) have been developed against tumor necrosis factor (TNF-α) overproduction to treat inflammatory and autoimmune diseases including rheumatoid arthritis, psoriatic arthritis, ankylosing spondylitis and Crohn’s disease. It is also suggested that a combination of TNF inhibitors and antibiotics might be beneficial for treatment of *S. aureus* sepsis^29^. TNF-α is released by monocytes, lymphocytes and macrophages, and facilitates macrophage apoptosis in response to *M.tb*^30^,^31^,^32^. TNF-α also recruits other immune cells to sites of inflammation. During immune response to pathogens or stimuli, macrophages synthesize and secrete TNF to the extracellular space via a series of secretion pathways. Many trafficking proteins are involved to deliver TNF outside the plasma membrane. Our MenA inhibitor potentially inhibited TNF release from THP-1 macrophages by either inhibiting one of trafficking proteins involved in secretion pathways, or binding to a membrane receptor that blocks activation of macrophages during the immune response. Earlier, it was reported that adenosine as agonist reduces TNF-α release from macrophage by activating the cAMP-elevating A(2 A) adenosine receptor (AR) subtype^33^,^34^. However, further study will be conducted to identify the anti-inflammatory role of these MenA inhibitors.

Unlike TNF-α and IL-6, we observed an increase in IL-1β release from THP-1 macrophages by stimulation by MenA inhibitors. Pro-inflammatory cytokine IL-1β is important in defending infection, but dysregulation of IL-1β is responsible for chronic inflammatory diseases and acute tissue injuries^35^. These results indicate that stimuli-mediated secretion of cytokines is more complicated. It is challenging to develop selective stimulating agents that block the release of cytokines of interest.

A series of long-chain compounds were developed to inhibit growth of MRSA and other gram-positive bacteria that utilize menaquinone under aerobic conditions. MenA inhibitors displayed a promising extracellular bactericidal activity against growth of MRSA and its biofilm. More importantly, naphthalene-based MenA inhibitors showed potential as inhibitors of extracellular bacterial growth and as immunomodulatory agents to reduce the release of IL-6 and TNF-α from macrophages. These MenA inhibitors could be used to reduce macrophage cytokine release and it would provide insight in the development of pharmaceuticals for MRSA septic shock and other inflammatory diseases.

## Methods

### Materials

All chemicals were purchased from Aldrich (St. Louis, MO, USA) and Across (Geel, Belgium). Microbial species are purchased from ATCC (Manassas, VA, USA). NMR spectroscopies were recorded on a Varian Unity/Inova-500 NB (500 MHz, Varian Medical Systems Inc. Palo Alto, CA, USA). Chemical shifts are reported in parts per million (ppm) downfield from TMS. Molecular weight is determined using MALDI TOF/TOF (Applied Biosystems, CA, USA).

### Minimum inhibitory concentration (MIC) against *S. aureus*

96-well plates were seeded with 5 × 10^5^ CFU/mL in a volume of 100 μL of tryptic soy broth (TSB) per well for *Staphylococcus aureus* assays. Compounds were dissolved in DMSO and serially diluted as 1:2 dilutions to give the final concentration ranging from 0.25 to 128 μg/mL per well. The final concentration of DMSO in the assay plates did not exceed 2%. Plates were incubated at 37 °C for 18–20 hours and MIC was determined by reading OD_625_. For *M.avium* and *M.abscessus*, 7H9 broth media was used for determination of MICs. The final concentration was 5 × 10^5^ CFU/mL in each well. The plate was incubated at 37 °C for 7–8 days and MICs were determined using a Tecan microplate reader (OD_625_) and resazurin reduction assay.

### Menaquinone rescue experiments with MRSA

Resazurin reduction assay was performed in 96-well plate. MRSA was cultured in TSB until OD_625_ reached 0.4~0.5 and diluted and plated in 96-well plate. MRSA (5 × 10^5^ CFU/mL/well) was treated with menaquinone (MK4, 0, 1, 5, 50, 500 μg/mL) in the presence of 0.5~128 μg/mL of inhibitor. The plate was incubated overnight at 37 °C followed by 20 μL of resazurin (0.15 mg/mL) was added to each well. The plate was incubated for 2–4 h and fluorescence was measured at 560 nm excitation/590 nm emission.

### Cytotoxicity Assay

To determine cytotoxicity of the inhibitors, THP-1 cells were differentiated into macrophages by PMA (7.5 ng/mL) for 24 h. THP-1 macrophages (5 × 10^5^/well) were treated with inhibitors in 48-well plates for 24 h at 37 °C in 5% carbon dioxide. Cells were washed with PBS buffer three times and serum-free culture medium was added to each well. Cytotoxicity was assessed using resazurin assay.

### *S. aureus* biofilm assay

Biofilm assays were performed using a method previously described with modification^36^. A 96 well plate was precoated with 20% human plasma (Sigma) in 0.05 M carbonate buffer, pH 9.6 at 4 °C for 24 h. MRSA was cultured overnight in TSB containing 3% NaCl and 0.5% glucose. The overnight culture was diluted with TSB/NaCl/Glucose media until OD_600_ reached 0.05. After carefully removing the liquid from the 96 well plate, 200 μL of MRSA dilution was added to each well in triplicate. The plate was incubated at 37 °C for 24 h and carefully washed with PBS twice. MH broth (200 μL) containing a compound was added to each well in triplicate and incubated at 37 °C for 24 h. After washing with PBS twice, the biofilm was scraped in 100 μL of PBS and serially diluted and plated on TSA to determine CFU.

### Colony morphology

MRSA was streaked and then incubated on tryptic soy agar (TSA) containing 0, 2, 4, 8 or 16 μg/mL of inhibitor **3a** or 4 μg/mL of **7C** or 0.025 μg/mL of rifampin at 37 °C for 96 h. MK-4 (100 μg/mL) was supplemented in TSA containing 8 μg/mL of **3a** for the rescue experiment.

### MenA inhibitor activity against extracellular *S. aureus* in the presence of human MDMs

Human monocyte-derived macrophages (MDM) were cultured at a concentration of 1 × 10^6^ cells/mL/well in a 24 well plate^37^. MRSA (USA 700) was cultured in TSB (10 mL) until OD_625_ reached 0.4~0.5 and then centrifuged at 14,000 rpm for 10 min. The pellets were resuspended in 10 mL of DMEM supplemented with 10% human serum (no antibiotics). The bacteria medium was diluted with DMEM containing an inhibitor to obtain 1 × 10^6^ CFU/mL and added to human MDMs (MOI = 1) in a 24-well plate. After 24 hour infection at 37 °C, the supernatants were serially diluted for CFU determination (TSA plates).

### Quantitation of cytokines

THP-1 cells were differentiated into macrophages by treatment with PMA (7.5 ng/mL). Levels of the human cytokines (GM-CSF, IFN-γ, IL-1β, IL-2, IL-4, IL-5, IL-6, IL-8, IL-10, TNF-α) released from THP-1 macrophages were determined using a multiplex kit from Life Technologies according to the manufacturer’s instructions. In brief, the 96 well filter plate was pre-wetted, and then 25 μL of the diluted bead suspension was added to each well and washed twice. 100 μL of samples and standards were added to each well. The plate was incubated at room temperature for 2 h on a shaker. Following washing, biotinylated detection antibody was added and incubated for 1 h. After washing, streptavidin-RPE was added to each well and incubated for 0.5 h. The plate was again washed, resuspended in 100 μL of the washing buffer, and read on the Luminex xMAP™ system (Bio-Rad, AtheNA multi-Lyte™, USA). All samples were run in triplicate and standards were run in duplicate.

### Statistical analysis

Statistical analysis was performed using Student’s t-test. Data are represented as mean ± SEM. Data are significant at p < 0.05.

## Additional Information

**How to cite this article**: Choi, S.-r. *et al*. Novel long-chain compounds with both Immunomodulatory and MenA Inhibitory activities against *Staphylococcus aureus* and its biofilm. *Sci. Rep.* **7**, 40077; doi: 10.1038/srep40077 (2017).

**Publisher's note:** Springer Nature remains neutral with regard to jurisdictional claims in published maps and institutional affiliations.

## Supplementary Material

Supporting Information

## Figures and Tables

**Table 1:**
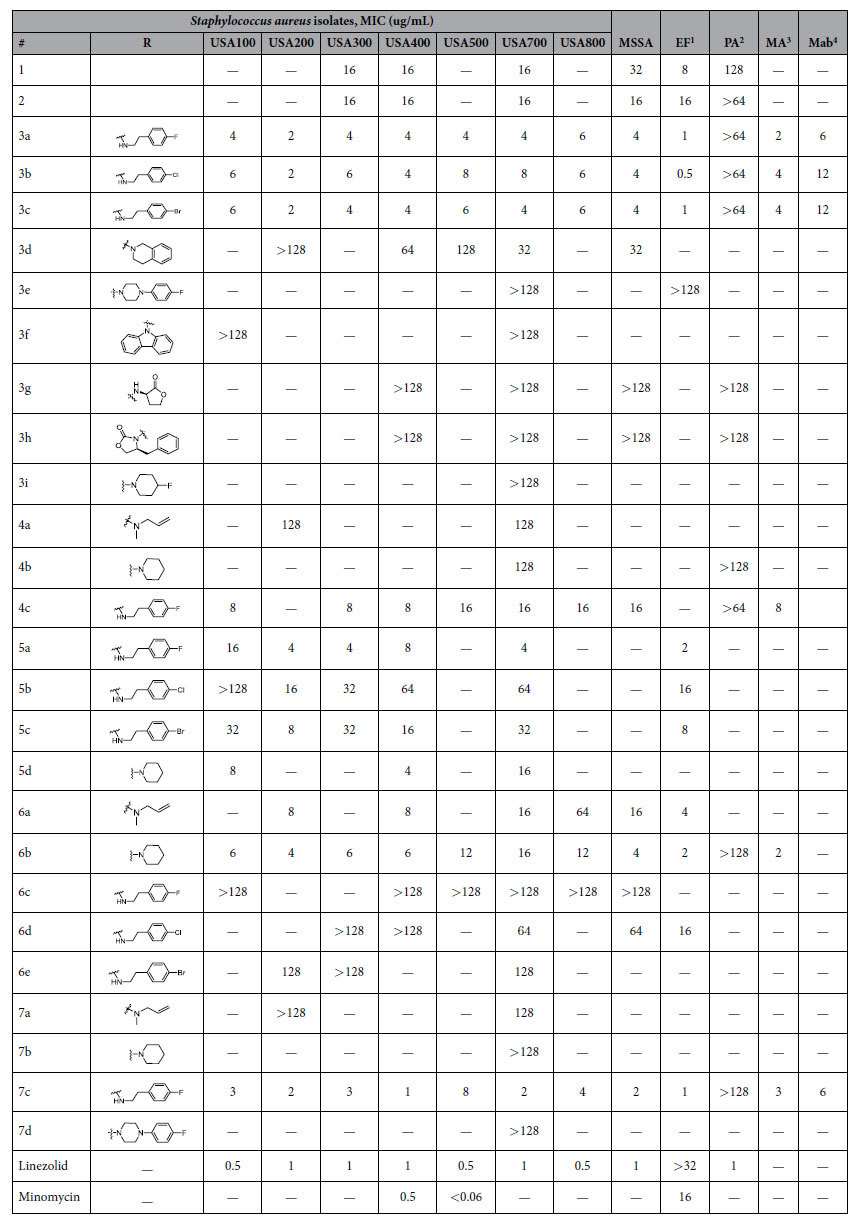
Minimum inhibitory concentration against MRSA strains and other bacteria.

^1^EF: *Enterococcus faecalis*.

^2^PA: *Pseudomonas aeruginosa*.

^3^MA: *Mycobacterium avium*.

^4^Mab: *Mycobacterium abscessus*, MSSA: Methicillin susceptible *Staphylococcus aureus*, (−): not determined.
