# Supplementary material for: Novel long-chain compounds with both immunomodulatory and MenA inhibitory activities against Staphylococcus aureus and its biofilm
Source: Sci Rep. 2017 Jan 10;7:40077. doi: 10.1038/srep40077 (PMC5223195; doi:10.1038/srep40077)
Supplement: Supporting Information [file srep40077-s1.pdf]

Supporting Information

Novel long-chain compounds with both immunomodulatory and MenA inhibitory activities against *Staphylococcus aureus* and its biofilm

Seoung-ryoung Choi, Joel Frandsen and Prabagaran Narayanasamy,*
Department of Pathology and Microbiology, College of Medicine, University of Nebraska Medical Center, Omaha, Nebraska 68198

Corresponding author: p.narayanasamy@unmc.edu

1. Synthesis of 3a-3i……………………………………………..……………..………….2
2. Synthesis of 4a-4c ……………………………..……………………………………………6
3. Synthesis of 5a-5c ……………………………………………………………..……………9
4. Synthesis of 6a-6c ……………………………………………………………………………11
5. Synthesis of 7a-7c ……………………………………………………………………………13
6. Figure S1. Menaquinone rescue experiments…………………………………………15
7. Figure S2. IL-8 released from macrophages treated with inhibitors………………..15
8. ^{1}H and ^{13}C NMR ……………………………………………….…………………..…16

1. Synthesis of 3a-3i

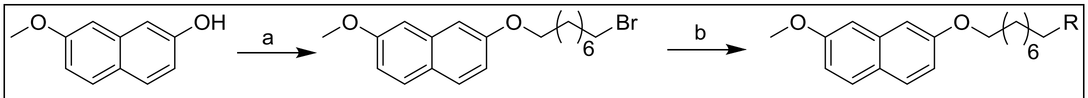

3a-h

a) K_2CO_3, 1,8-dibromooctane, CH_3CN, 60 °C, b) K_2CO_3, 1° or 2° amine, CH_3CN, 60 °C.

2-((8-bromooctyl)oxy)-7-methoxynaphthalene. To a solution of 7-methoxy-2-naphthol (0.5 g, 2.87 mmol) and anhydrous K_2CO_3 (5.74 mmol) in acetonitrile (10 mL) was added 1,8-dibromooctane (7.17 mmol) at rt. The mixture was stirred overnight at 60 °C. The salts were filtered off and the solvent was removed under reduced pressure. The residue was dissolved in ethyl acetate, washed with water and brine and dried over $MgSO_4$. The residue was purified by column chromatography (ethyl acetate:hexane = 1:19) to give a pale yellow solid (0.682 mg, 65%). ^{1}H NMR ($CDCl_3$): δ 7.66 (d, *J* = 8.5, 2H), 7.06 (m, 2H), 7.00 (m, 2H), 4.07 (t, *J* = 6.5, 2H), 3.43 (t, *J* = 7.0, 2H), 1.87 (m, 4H), 1.47 (m, 8H); ^{13}C NMR: δ 135.88, 129.07, 129.02, 124.15, 116.29, 115.85, 105.99, 105.14, 67.82, 55.19, 33.98, 32.74, 29.17, 29.16, 28.66, 28.06, 25.98; MS (m/z) calcd. for $C_{19}H_{25}BrO_2$ 366.1017 [M+2], found 366.092 $[M+2]^+$.

General procedure for amination. To a mixture of alkyl halide and anhydrous K_2CO_3 (2 eq.) in acetonitrile was added amine compound (2 eq.) at rt. The mixture was stirred overnight at 60 °C. The salts were filtered off and the solvent was removed under reduced pressure. The residue was dissolved in CH_2Cl_2, washed with water and brine and dried over $MgSO_4$. The residue was purified by column chromatography (silica, 5-10 % MeOH in CH_2Cl_2).

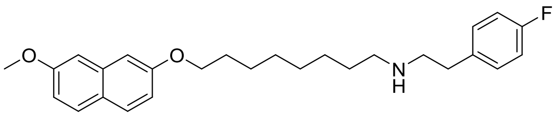

N-(4-fluorophenethyl)-8-((7-methoxynaphthalen-2-yl)oxy)octan-1-amine (3a). To a mixture of 2-((8-bromooctyl)oxy)-7-methoxynaphthalene (1.02 mmol) and K_2CO_3 (2 mmol) in acetonitrile was added 4-chlorophenylethylamine (2 mmol) at rt. Purification by fresh column chromatogranpy (silica, 5-10% MeOH in CH_2Cl_2) gave a white solid (160 mg, 37 %); ^{1}H NMR ($CDCl_3$) δ 7.64 (d, *J* = 9.0, 2H), 7.16 (d, *J*=5.5, 1H), 7.15 (d, *J*=5.5, 1H), 7.08 (d, *J*=5.5, 2H),

7.05 (m, 2H), 6.98 (m, 4H), 4.02 (t, *J*=6.5, 2H), 3.88 (s, 3H), 2.84 (t, *J*=6.5, 2H), 2.79 (t, *J*=6.5, 2H), 2.61 (t, *J*=7.0, 2H), 1.81 (p, *J*=6.5, 2H), 1.47 (m, 4H), 1.31 (m, 6H); ^{13}C NMR ($CDCl_3$) δ 162.3, 160.4, 158.1, 157.6, 135.9, 135.7, 135.6, 130.0, 129.0, 124.1, 116.2, 115.8, 115.2, 115.0, 106.0, 105.1, 67.8, 55.1, 51.2, 49.8, 35.5, 29.9, 29.4, 29.27, 29.2, 27.1, 25.9; MS [M+H$^+$]; calcd. for $C_{27}H_{35}FNO_2$ 424.2652, found 424.21.

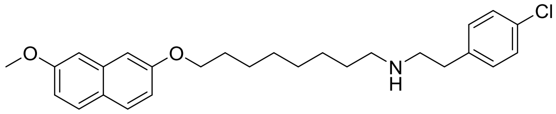

N-(4-chlorophenethyl)-8-((7-methoxynaphthalen-2-yl)oxy)octan-1-amine (3b). To a mixture of 2-((8-bromooctyl)oxy)-7-methoxynaphthalene (0.27 mmol) and K_2CO_3 (0.54 mmol) in acetonitrile was added 4-chlorophenylethylamine (0.54 mmol) at rt. Purification by fresh column chromatogranpy (silica, 5% MeOH in CH_2Cl_2) gave a white solid (80 mg, 67 %). ^{1}H NMR ($CDCl_3$) δ 7.62 (d, *J*=8.5, 2H), 7.23 (d, *J*=8.5, 2H), 7.08 (d, *J*=8.5, 2H), 7.02 (m, 2H), 6.98 (m, 2H), 4.02 (t, *J*=6.5, 2H), 3.88 (s, 3H), 2.84 (t, *J*=6.5, 2H), 2.79 (t, *J*=6.5, 2H), 2.61 (t, *J*=7.5, 2H), 1.81 (p, *J*=6.5, 11.0, 2H), 1.47 (m, 4H), 1.31 (m, 6H); ^{13}C NMR ($CDCl_3$) δ 158.06, 157.63, 138.19, 135.87, 131.88, 129.95, 129.02, 128.97,128.50, 124.13, 116.26, 115.80, 105.99, 105.15, 67.83, 55.13, 50.78, 49.63, 35.31, 29.59, 29.34, 29.22, 29.16, 27.16, 25.98; MS [M+H$^+$]; calcd. for $C_{27}H_{34}ClNO_2$ 440.2356, found 440.19.

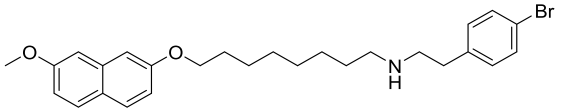

N-(4-bromophenethyl)-8-((7-methoxynaphthalen-2-yl)oxy)octan-1-amine (3c). Fresh column (5% MeOH in CH_2Cl_2) was performed to give white solid as a product (67 mg, 52%). ^{1}H NMR ($CDCl_3$) δ 7.63 (d, *J*=8.5, 2H), 7.41 (d, *J*=8.5, 2H), 7.08 (d, J=8.0, 2H), 7.04 (m, 2H), 6.99 (m, 2H), 4.05 (t, *J*=6.5, 2H), 3.90 (s, 3H), 2.90 (t, *J*=7.0, 2H), 2.81 (t, *J*=7.0, 2H), 2.67 (t, *J*=7.5, 2H), 1.82 (p, *J*=7.0, 11.0, 2H), 1.49 (m, 4H), 1.34 (m, 6H); ^{13}C NMR ($CDCl_3$) δ 158.05, 157.64, 138.37, 135.88, 131.52, 130.37, 129.04, 128.98, 124.15, 120.06, 116.28, 115.82, 106.02, 105.17, 67.83, 55.16, 50.56, 49.50, 35.03, 29.31, 29.25, 29.23, 29.17, 27.10, 25.99; MS [M+H$^+$]; calcd. for $C_{27}H_{35}BrNO_2$ 484.1851, found 484.15, 486.15 $[M+H+2]^+$.

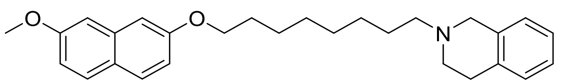

2-(8-((7-methoxynaphthalen-2-yl)oxy)octyl)-1,2,3,4-tetrahydroisoquinoline (3d). Purification by fresh column chromatogranpy (silica, 5% MeOH in CH_2Cl_2) gave a yellow oil (83 mg, 73 %). ^{1}H NMR ($CDCl_3$) δ 7.65 (d, J=9.0, 2H), 7.10 (m, 3H), 7.00 (m, 5H), 4.07 (t, J=6.5, 2H), 3.91 (s, 3H), 3.63 (s, 2H), 2.91 (t, J=6, 2H), 2.73 (t, J=7.5, 2H), 1.85 (p, J=6.5, 11.0, 2H), 1.62 (m, 2H), 1.52 (m, 2H), 1.40 (m, 6H); ^{13}C NMR ($CDCl_3$) δ 158.16, 157.74, 135.96, 134.94, 134.39, 129.12, 129.06, 128.63, 126.60, 126.05, 125.54, 124.23, 116.38, 115.89, 106.09, 105.24, 67.97, 58.57, 56.27, 55.25, 51.01, 29.55, 29.36, 29.27, 29.12, 27.57, 27.20, 26.06; MS [M+Na$^+$]; calcd. for $C_{28}H_{35}NO_2Na$ 440.2565, found 440.22.

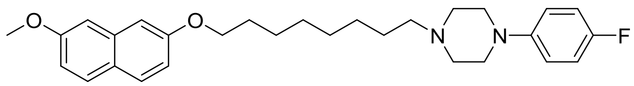

1-(4-fluorophenyl)-4-(8-((7-methoxynaphthalen-2-yl)oxy)octyl)piperazine (3e). Purification by fresh column chromatogranpy (silica, 2% MeOH in CH_2Cl_2) gave a white solid (100 mg, 79 %). ^{1}H NMR ($CDCl_3$) δ 7.66 (d, *J*=9.0, 2H), 7.06 (m, 2H), 7.01 (m, 2H), 6.97 (t, *J*=8.0, 2H), 6.88 (m, 2H), 4.07 (t, *J*=6.5, 2H), 3.91 (s, 3H), 3.13 (t, *J*=5.0, 4H), 2.60 (t, *J*=5.0, 4H), 2.40 (t, *J*=8.0, 2H), 1.86 (p, *J*=7.0, 11.0, 2H), 1.56 (m, 4H), 1.40 (m, 6H); ^{13}C NMR ($CDCl_3$) δ 158.02, 157.99, 157.66, 156.09, 147.99, 147.98, 135.90, 129.06, 129.00, 124.16, 117.67, 117.61, 116.29, 115.83, 115.49, 115.31, 106.01, 105.17, 67.86, 58.70, 55.16, 53.24, 50.08, 29.43, 29.25, 29.20, 27.45, 26.83, 26.00; MS [M+H$^+$]; calcd. for $C_{29}H_{38}FN_2O_2$ 465.2917, found 465.23.

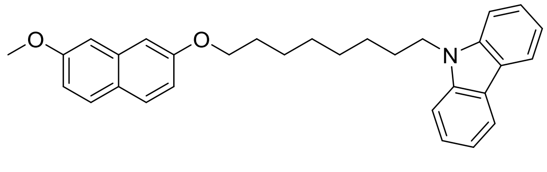

9-(8-((7-methoxynaphthalen-2-yl)oxy)octyl)-9*H*-carbazole (3f). Purification by fresh column chromatography (silica, ethyl acetate:hexane = 1:10) gave a yellow oil (83 mg, 73 %). ^{1}H NMR ($CDCl_3$) δ 8.09 (d, *J* = 9.0, 2H), 7.63 (d, *J* = 9.0, 2H), 7.45 (t, *J* = 7.0, 2H), 7.39 (d, *J* = 8.5, 2H), 7.21 (t, *J* = 8.0, 2H), 7.02 (d, J = 2.0, 2H), 6.98 (t, *J* = 2.5, 1H), 6.96 (t, *J* = 2.5, 1H), 4.29 (t, *J* = 6.0, 2H), 4.01 (t, *J* = 6.0, 2H), 3.89 (s, 3H), 1.87 (p, *J* = 7.0 , 2H), 1.85 (p, *J* = 7.0, 2H), 1.38 (m, 8H); ^{13}C NMR ($CDCl_3$) δ 158.13, 157.67, 140.40, 135.92, 129.09, 129.03, 125.54, 124.20,

122.79, 120.31, 118.68, 116.32, 115.86, 108.61, 106.06, 105.21, 67.83, 55.22, 43.02, 29.32, 29.20, 29.15, 28.96, 27.22, 26.00; MS [M+H$^+$]; calcd. for $C_{31}H_{34}NO_2$ 452.2590, found 452.21.

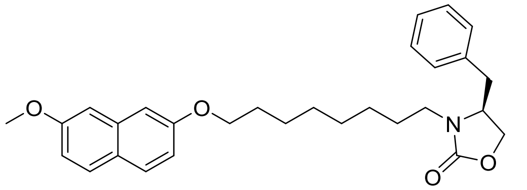

(S)-4-benzyl-3-(8-((7-methoxynaphthalen-2-yl)oxy)octyl)oxazolidin-2-one (3g). White solid (110 mg, 88.7 %). ^{1}H NMR ($CDCl_3$) δ 7.66 (d, *J* = 8.5, 2H), 7.35 (t, *J* = 7.5, 2H), 7.29 (m, 1H), 7.17 (d, *J* = 7.5, 2H), 7.07 (m, 2H), 7.01 (m, 2H), 4.17 (m, 1H), 4.08 (t, *J* = 6.5, 2H), 4.01 (m, 2H), 3.92 (s, 3H), 3.53 (m, 1H), 3.10 (m, 2H), 2.67 (dd, *J* = 12, 9.0, 1H), 1.87 (p, *J* = 7.0, 2H), 1.53 (m, 4H), 1.41 (m, 6H); ^{13}C NMR ($CDCl_3$) δ 158.1, 158.0, 157.6, 135.8, 135.5, 129.0, 128.98, 128.93, 128.8, 127.1, 124.1, 116.2, 115.8, 106.0, 105.1, 67.8, 66.6, 55.8, 55.1, 42.0, 38.4, 29.18, 29.14, 29.1, 27.3, 26.5, 25.9; MS [M+Na$^+$]; calcd. for $C_{29}H_{35}NO_4Na$ 484.2464, found 484.19.

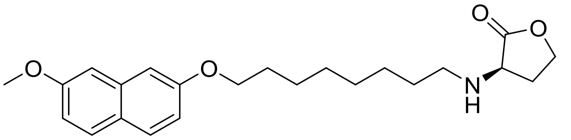

(R)-3-((8-((7-methoxynaphthalen-2-yl)oxy)octyl)amino)dihydrofuran-2(3H)-one (3h). Silica column chromatography (CH_2Cl_2:methanol = 95:5). White solid (20 mg, 15 %). ^{1}H NMR ($CDCl_3$) δ 7.64 (d, *J* = 8.5, 2H), 7.04 (d, *J* = 2.5, 2H), 6.98 (m, 2H), 4.38 (td, *J* = 2.0, 9.0, 1H), 4.17 (m, 1H), 4.05 (t, *J* = 6.5, 2H), 3.53 (dd, *J* = 10, 8.0, 1H), 2.72 (m, 1H), 2.62 (m, 1H), 2.48 (m, 1H), 2.09 (m, 1H), 1.83 (p, *J* = 6.5, 2H), 1.51 (m, 4H), 1.36 (m, 6H); ^{13}C NMR ($CDCl_3$) δ 177.2, 158.1, 157.6, 135.8, 129.0, 128.9, 124.1, 116.2, 115.8, 106.0, 105.1, 67.8, 65.6, 56.4, 55.1, 48.1, 30.2, 29.9, 29.3, 29.2, 29.1; MS (m/z); calcd. for $C_{23}H_{31}NO_4Na$ 408.2151, found 408.16 [M+Na$^+$], 424.14 [M+K$^+$].

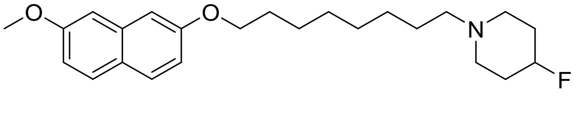

4-fluoro-1-(8-((7-methoxynaphthalen-2-yl)oxy)octyl)piperidine (3i). Silica column chromatography (CH_2Cl_2:methanol = 95:5). white solid (90 mg, 86 %). ^{1}H NMR ($CDCl_3$) δ 7.65 (s, 1H), 7.63 (s, 1H), 7.04 (d, *J* = 3.5, 2H), 6.98 (m, 2H), 4.71 (br, 0.5H), 4.61 (br, 0.5H), 4.04 (t,

J = 7, 2H), 3.89 (s, 3H), 2.57 (br, 2H), 2.35 (br, 2H), 2.32 (t, *J* = 8, 2H), 1.86 (m, 6H), 1.49 (m, 4H), 1.36 (m, 6H); ^{13}C NMR ($CDCl_3$) δ 158.0, 157.6, 135.8, 129.0, 128.9, 124.1, 116.2, 115.8, 105.9, 105.1, 67.8, 58.6, 55.1, 49.5, 31.5, 31.3, 29.4, 29.2, 29.1, 27.4, 27.0, 25.9; MS [M+Na$^+$]; calcd. for $C_{24}H_{34}FNO_2Na$ 410.2471, found 410.18.

2. Synthesis of **4a-4c**

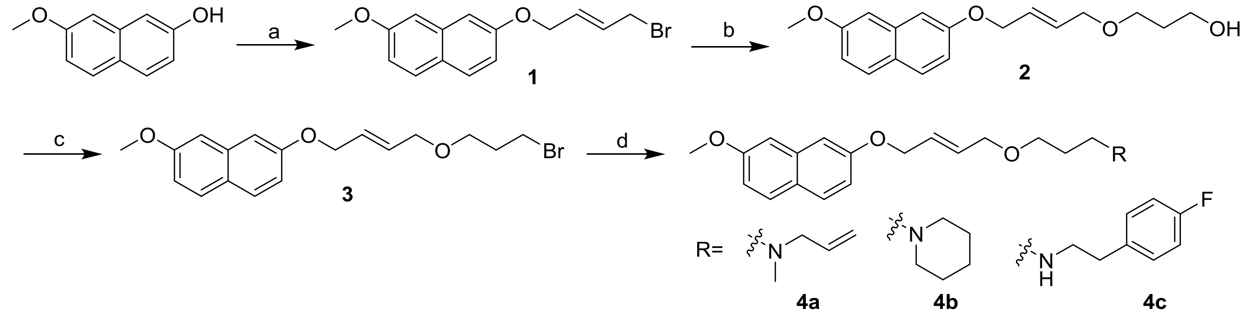

a) K_2CO_3, 1,4-dibromobut-2-ene, CH_3CN, 60 °C, b) K_2CO_3,1,3-propandiol, CH_3CN, c) PBr_3, CH_2Cl_2, d) K_2CO_3, 1° or 2° amine, CH_3CN, 60 °C.

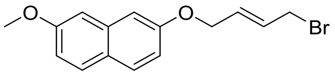

(E)-2-((4-bromobut-2-en-1-yl)oxy)-7-methoxynaphthalene (2). To a mixture of 7-methoxy-2-naphthol (5.45 mmol) and K_2CO_3 (11 mmol) in acetonitrile (10 mL) was added 1,4-dibromobutene (13.6 mmol) at 0 °C. The mixture was stirred at 0 °C for 8 h. K_2CO_3 was filtered off and acetonitrile was removed under reduced vacuum. The residue was dissolved in ethyl acetate and washed with water and brine. The organic layer was dried over $MgSO_4$. Purification by flash column chromatography (silica gel, ethyl acetate:hexane = 1:8) gave a white solid (1.42g, 85 %). ^{1}H NMR ($CDCl_3$) δ 7.68 (d, *J*=3.5, 1H), 7.66 (d, *J*=4.0, 1H), 7.03 (m, 4H), 6.11 (m, 1H), 6.05 (m, 1H), 4.64 (d, *J*=5.0, 2H), 4.01 (d, *J*=7.5, 2H), 3.17 (s, 3H); ^{13}C NMR ($CDCl_3$) δ 158.16, 156.83, 135.73, 129.98, 129.18, 129.06, 124.35, 116.16, 116.05, 106.43, 105.22, 67.10, 55.17, 31.34. MS (M+H$^+$); calcd. for $C_{15}H_{16}BrO_2$ 307.0334, found 307.11.

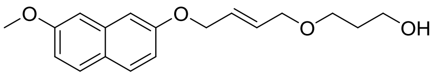

(E)-3-((4-((7-methoxynaphthalen-2-yl)oxy)but-2-en-1-yl)oxy)propan-1-ol (3). To a stirred solution of 1,3-propanediol (10.58 mmol) in DMF (5 mL) at 0 C was added NaH (12.7 mmol).

The bromide compound **2** in DMF (5 mL) was slowly added at 0 C. The mixture was stirred at room temperature for 6 h and quenched with 1N HCl. The mixture was extracted with EtOAc, washed with water and brine, and dried over $MgSO_4$. Silica column chromatography (ethyl acetate:Hexane = 1:3) was performed to give yellow oil (523 mg, 82 %). ^{1}H NMR ($CDCl_3$) δ 7.65 (d, *J*=3.5, 1H), 7.63 (d, J=3.5, 1H), 7.01 (m, 4H), 5.98 (m, 2H), 4.62 (d, *J*=3.5, 2H), 4.03 (d, *J*=4.0, 2H), 3.89 (s, 3H), 3.75 (t, *J*=6.0, 2H), 3.61 (t, *J*=6.0, 2H), 1.84 (p, *J*=6.0, 8.5, 2H); ^{13}C NMR ($CDCl_3$) δ 158.07, 156.95, 135.72, 129.91, 129.06, 129.00, 127.60, 124.25, 116.12, 116.02, 106.35, 105.16, 70.77, 69.09, 67.65, 61.37, 55.12, 32.03. MS [M+Na$^+$]; calcd. for $C_{18}H_{23}O_4Na$ 325.1416, found 325.06.

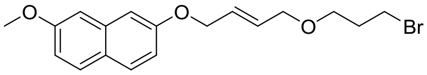

(E)-2-((4-(3-bromopropoxy)but-2-en-1-yl)oxy)-7-methoxynaphthalene (4).

Triphenylphosphine (1.62 mmol) was added to the mixture of the alcohol **3** (1.24 mmol) and CBr_4 (1.62 mmol) in CH_2Cl_2 (10 mL) at room temperature. The mixture was stirred at rt for 1.5 h. the solvent was removed under reduced vacuum and column chromatography (silica, ethylacetate:hexane = 1:6) was performed to afford a yellow oil as product (0.43 g, 95 %). ^{1}H NMR ($CDCl_3$) δ 7.72 (d, *J*=3.5, 1H), 7.65 (d, *J*=3.5, 1H), 7.03 (m, 4H), 6.01 (m, 2H), 4.65 (d, *J*=4.5, 2H), 4.05 (d, *J*=4.5, 2H), 3.91 (s, 3H), 3.58 (t, *J*=6.0, 2H), 3.52 (t, *J*=6.5, 2H), 2.12 (p, *J*=6.5, 2H); ^{13}C NMR ($CDCl_3$) δ 158.13, 157.02, 135.77, 130.07, 129.11, 129.05, 127.56, 124.29, 116.19, 116.07, 106.41, 105.18, 70.69, 67.73, 67.66, 55.18, 32.80, 30.54. MS (m/z); calcd. for $C_{18}H_{22}BrO_3$ 365.0752, found 365.02 [M+H$^+$], 366.03 [M+2]$^+$.

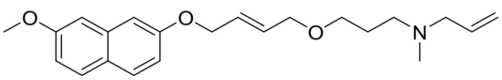

(E)-N-(3-((4-((7-methoxynaphthalen-2-yl)oxy)but-2-en-1-yl)oxy)propyl)-N-methylprop-2-en-1-amine (4a). A pale yellow oil (57 mg, 59 %). ^{1}H NMR ($CDCl_3$) δ 7.65 (d, *J*=2.5, 1H), 7.63 (d, *J*=2.5, 1H), 7.03 (m, 4H), 6.00 (m, 2H), 5.87 (m, 1H), 5.16 (m, 2H), 4.63 (d, *J*=4.0, 2H), 4.02 (d, *J*=4.0, 2H), 3.90 (s, 3H), 3.49 (t, *J*=6.5, 2H), 3.02 (d, *J*=6.5, 2H), 2.46 (t, *J*=7.5, 2H), 1.80 (p, *J*=6.5, 2H); ^{13}C NMR ($CDCl_3$) δ 158.1, 157.0, 135.7, 135.0, 130.04, 129.1, 129.0, 127.3, 124.3,

117.9, 116.2, 116.0, 106.4, 105.2, 70.6, 68.7, 67.8, 60.7, 55.2, 54.0, 41.7, 27.4. MS [M+H$^+$]; calcd. for $C_{22}H_{30}NO_3$ 356.2226, found 356.15

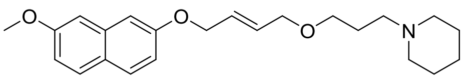

(E)-1-(3-((4-((7-methoxynaphthalen-2-yl)oxy)but-2-en-1-yl)oxy)propyl)piperidine (4b). A pale yellow solid (86 mg, 86 %). ^{1}H NMR ($CDCl_3$) δ 7.64 (d, *J*=3, 1H), 7.63 (d, *J*=3, 1H), 7.03 (m, 4H), 5.99 (m, 2H), 4.63 (d, *J*=4.0, 2H), 4.01 (d, *J*=4.0, 2H), 3.89 (s, 3H), 3.48 (t, *J*=6.5, 2H), 2.40 (m, 6H), 1.81 (p, *J*=6.5, 2H), 1.60 (p, *J*=6.0, 4H), 1.43 (m, 2H); ^{13}C NMR ($CDCl_3$) δ 158.1, 157.0, 135.7, 130.4, 129.07, 129.03, 127.2, 124.2, 116.1, 116.0, 106.3, 105.1, 70.5, 68.9, 67.7, 56.1, 55.1, 54.4, 26.9, 25.6, 24.2. MS (m/z); calcd. 370.2382 for $C_{23}H_{32}NO_3$, found 370.21 [M+H$^+$], 392.19 [M+Na]$^+$.

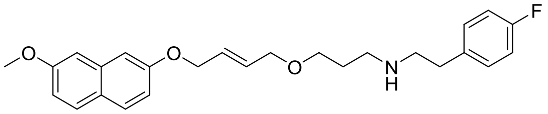

(E)-N-(4-fluorophenethyl)-3-((4-((7-methoxynaphthalen-2-yl)oxy)but-2-en-1-yl)oxy)propan-1-amine (4c). To a mixture of the bromide (0.27 mmol) and K_2CO_3 (0.41 mmol) in acetonitrile (5 mL) was added 4-fluorophenylethylamine (0.41 mmol) at room temperature. The mixture was stirred at 50-60 °C for overnight. The solvent was removed under reduced vacuum and the residue was dissolved in CH_2Cl_2 and washed with water and brine. The organic layer was dried under MgSO4. Fresh column (5-10 % MeOH in CH_2Cl_2) was performed to give a white solid as a product (64 mg, 56%). ^{1}H NMR ($CDCl_3$) δ 7.65 (d, *J*=3.0, 1H), 7.63 (d, *J*=3.0, 1H), 7.14 (d, *J*=5.5, 1H), 7.15 (d, *J*=5.5, 1H), 7.01 (m, 6H), 5.95 (m, 2H), 4.63 (d, *J*=5.0, 2H), 3.98 (d, *J*=5.0, 2H), 3.89 (s, 3H), 3.49 (t, *J*=6.0, 2H), 2.86 (t, *J*=7.0, 2H), 2.79 (t, *J*=7.0, 2H), 2.75 (t, J=7.0, 2H), 1.79 (p, *J*=6.5, 10.0, 2H); ^{13}C NMR ($CDCl_3$) δ 162.39, 160.44, 158.13, 157.01, 135.77, 135.28, 130.19, 129.97, 129.10, 127.45, 124.29, 116.16, 116.06, 115.25, 115.08, 106.40, 105.19, 70.60, 68.81, 67.73, 55.16, 50.94, 47.07, 35.04, 29.54. MS [M+H$^+$]; calcd. for $C_{26}H_{31}FNO_3$ 424.2288, found 424.20

3. Synthesis of 5a-5c

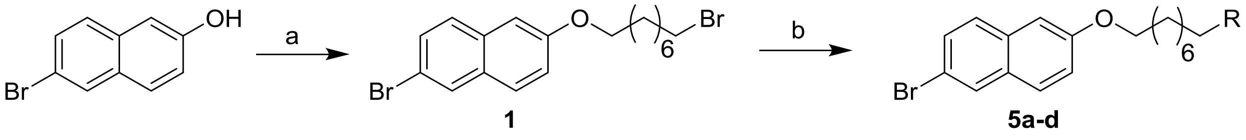

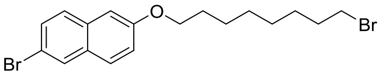

2-bromo-6-((8-bromooctyl)oxy)naphthalene (1). Silica column chromatography (ethyl acetate:hexane = 1:10), White solid (3.1 g, 75 %). ^{1}H NMR ($CDCl_3$) δ 7.89 (s, 1H), 7.62 (d, *J* = 8.5, 1H), 7.57 (d, *J* = 8.5, 1H), 7.49 (dd, *J* = 2.5, 7.0, 1H), 7.17 (dd, *J* = 2.5, 7.0, 1H), 7.07 (d, *J* = 2.0), 4.04 (t, *J* = 6.5, 2H), 3.41 (t, *J* = 7.0, 2H), 1.87 (m, 4H), 1.40 (m, 6H); ^{13}C NMR ($CDCl_3$) δ 157.3, 133.0, 129.8, 129.5, 129.4, 128.3, 128.2, 119.9, 116.8, 106.4, 67.9, 33.9, 33.8, 32.7, 32.6, 29.1, 29.0, 28.6; MS [M+H$^+$]; calcd. for $C_{18}H_{23}Br_2O$ 413.0116, found 413.92.

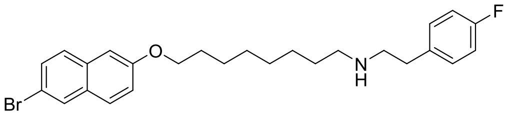

8-((6-bromonaphthalen-2-yl)oxy)-N-(4-fluorophenethyl)octan-1-amine (5a). Silica column chromatography (ethyl acetate:methanol = 95:5), White solid (140 mg, 62 %). ^{1}H NMR ($CDCl_3$) δ 9.70 (br, NH), 7.89 (s, 1H), 7.61 (d, *J* = 9.0, 1H), 7.56 (d, *J* = 9.0, 1H), 7.47 (dd, *J* = 1.5, 8.5, 1H), 7.20 (d, *J* = 5.5, 1H), 7. 19 (d, *J* = 5.5, 1H), 7.13 (dd, *J* = 2.0, 8.5, 1H), 7.07 (d, *J* = 2.0, 1H), 6.98 (d, *J* = 8, 1H), 6.96 (d, *J* = 8, 1H), 3.98 (t, *J* = 6.5, 2H), 3.25 (t, *J* = 5.5, 2H), 3.12 (t, *J* = 5.5, 2H), 2.93 (t, *J* = 8.0, 2H), 1.80 (p, *J* = 8.5, 16, 2H) 1.75 (p, *J* = 6.5, 14.5, 2H), 4H), 1.36 (m, 8H); ^{13}C NMR ($CDCl_3$) δ 157.3, 133.0, 132.1, 130.2, 130.1, 129.9, 129.59, 129.52, 128.3, 128.2, 119.9, 116.8, 115.8, 115.6, 106.5, 67.8, 49.1, 48.0, 31.5, 29.08, 29.04, 28.9, 26.7, 26.0, 25.9; MS [M+H$^+$]; calcd. for $C_{26}H_{32}BrFNO$ 472.1621, found 472.10, 474.10 [M+H+2]$^+$.

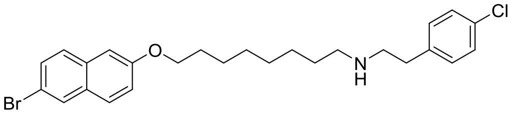

8-((6-bromonaphthalen-2-yl)oxy)-N-(4-chlorophenethyl)octan-1-amine (5b). Silica column chromatography (ethyl acetate:methanol = 95:5), White solid (140 mg, 62 %). ^{1}H NMR ($CDCl_3$) δ 9.70 (br, NH), 7.89 (s, 1H), 7.61 (d, *J* = 9.0, 1H), 7.56 (d, *J* = 9.0, 1H), 7.47 (dd, *J* = 1.5, 8.5, 1H), 7.20 (d, *J* = 5.5, 1H), 7. 19 (d, *J* = 5.5, 1H), 7.13 (dd, *J* = 2.0, 8.5, 1H), 7.07 (d, *J* = 2.0, 1H),

6.98 (d, *J* = 8, 1H), 6.96 (d, *J* = 8, 1H), 3.98 (t, *J* = 6.5, 2H), 3.25 (t, *J* = 5.5, 2H), 3.12 (t, *J* = 5.5, 2H), 2.93 (t, *J* = 8.0, 2H), 1.80 (p, *J* = 8.5, 2H) 1.75 (p, *J* = 6.5, 2H), 1.36 (m, 8H); ^{13}C NMR ($CDCl_3$) δ 157.3, 133.0, 132.4, 130.0, 129.9, 129.6, 129.52, 128.4, 128.3, 120.0, 116.8, 106.5, 67.9, 50.1, 49.1, 34.0, 29.24, 29.2, 29.1, 28.3, 27.0, 25.9; MS [M+H$^+$]; calcd. for $C_{26}H_{32}BrClNO$ 488.1356, found 488.09.

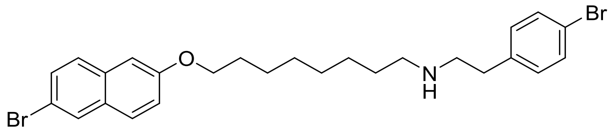

8-((6-bromonaphthalen-2-yl)oxy)-N-(4-bromophenethyl)octan-1-amine (5c). Silica column chromatography (ethyl acetate:methanol = 95:5), White solid (130 mg, 51 %). ^{1}H NMR ($CDCl_3$) δ 7.89 (s, 1H), 7.62 (d, *J* = 9.0, 1H), 7.57 (d, *J* = 9.0, 1H), 7.47 (dd, *J* = 2, 9, 1H), 7.39 (d, *J* = 8.5, 1H), 7. 15 (dd, *J* = 2.0, 9.0, 1H), 7.08 (m, 3H), 4.03 (t, *J* = 6.5, 2H), 2.87 (t, *J* = 7.0, 2H), 2.79 (t, *J* = 7.0, 2H), 2.63 (t, *J* = 7.5, 2H), 2.21 (br, NH), 1.80 (p, *J* = 7.0, 2H), 1.50 (m, 4H), 1.35 (m, 8H); ^{13}C NMR ($CDCl_3$) δ 157.3, 138.5, 133.0, 131.5, 130.4, 129.8, 129.59, 129.50, 128.3, 128.2, 120.05, 120.02, 106.4, 68.0, 50.6, 49.5, 35.2, 29.4, 29.3, 29.2, 29.1, 27.1, 25.9; MS [M+H$^+$]; calcd. for $C_{26}H_{32}Br_2NO$ 532.0851, found 532.08.

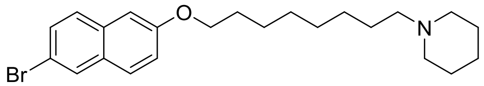

1-(8-((6-bromonaphthalen-2-yl)oxy)octyl)piperidine (5d). Silica column chromatography (ethyl acetate:methanol = 95:5), White solid (372 mg, 88 %). ^{1}H NMR ($CDCl_3$) δ 7.83 (s, 1H), 7.59 (d, *J* = 9.0, 1H), 7.51 (d, *J* = 9.0, 1H), 7.41 (d, *J* = 7.5, 1H), 7.08 (d, *J* = 9, 1H), 7. 01 (s, 1H), 3.96 (t, *J* = 6, 2H), 2.56 (br, 4H), 2.45 (t, *J* = 8, 2H), 1.76 (t, *J* = 7.0, 2H), 1.67 (m, 4H), 1.55 (br, 2H), 1.42 (br, 4H), 1.26 (m, 6H); ^{13}C NMR ($CDCl_3$) δ 177.0, 157.2, 132.9, 129.7, 129.3, 129.2, 128.1, 119.8, 116.6, 106.3, 67.8, 58.2, 53.4, 29.08, 29.01, 28.9, 27.1, 25.7, 25.1, 24.3, 23.3; MS (m/z); calcd. for $C_{23}H_{33}BrNO$ 418.1746, found 418.09 [M+H$^+$], 420.09 [M+H+2]$^+$.

4. Syntheis of 6a-6e

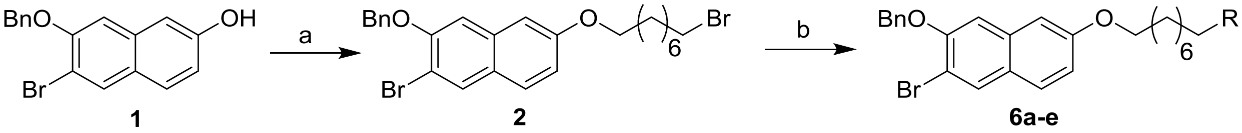

a) K_2CO_3, 1,8-dibromooctane, CH_3CN, 60 °C, b) K_2CO_3, 1° or 2° amine, CH_3CN, 60 °C.

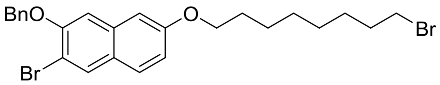

3-(benzyloxy)-2-bromo-6-((8-bromooctyl)oxy)naphthalene (2). To a mixture of **1** (1.51 mmol) and K_2CO_3 (3.79 mmol) in acetonitrile was added 1,8-dibromooctane (8.0 mmol) at rt. Purification by fresh column chromatogranpy (hexane:ethyl acetate = 1:10) gave a yellow solid (678 mg, 87 %). ^{1}H NMR ($CDCl_3$) δ 8.01 (s, 1H), 7.59 (d, *J* =9.0, 1H), 7.57 (d, *J* =8.0, 2H), 7.45 (t, *J* =7.5, 2H), 7.37 (t, *J* =7.5, 1H), 7.13 (s, 1H), 7.05 (dd, *J* =2.0, 9.0, 1H), 7.02 (s, 1H), 5.26 (s, 2H), 4.05 (t, *J* =6.5, 2H), 3.45 (t, *J* =7.0, 2H), 1.90 (m, 4H), 1.42 (m, 8H); ^{13}C NMR ($CDCl_3$) δ 157.78, 152.98, 136.45, 134.77, 131.90, 128.52, 128.10, 127.83, 126.87, 124.82, 117.37, 110.60, 107.49, 105.80, 70.53, 67.87, 33.93, 32.71, 29.12, 28.62, 28.02, 25.93; MS (m/z); calcd. for $C_{25}H_{29}Br_2O_2$ 519.0534, found 439.06 $[M-Br]^+$.

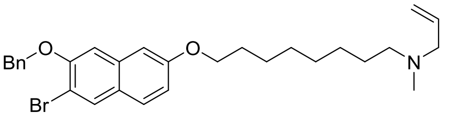

N-allyl-8-((7-(benzyloxy)-6-bromonaphthalen-2-yl)oxy)-N-methyloctan-1-amine (6a). To a mixture of **2** (0.1 mmol) and K_2CO_3 (0.3 mmol) in acetonitrile was added N-allylmethylamine (0.3 mmol) at rt. Purification by fresh column chromatogranpy (silica, 5% MeOH in CH_2Cl_2) gave a colorless oil (40 mg, 78 %). ^{1}H NMR ($CDCl_3$) δ 7.98 (s, 1H), 7.57 (d, *J* =9.0, 1H), 7.54 (d, *J* =7.5, 2H), 7.42 (t, *J*=7.5, 2H), 7.34 (t, *J*=7.5, 1H), 7.12 (s, 1H), 7.02 (dd, *J* =2.0, 9.0, 1H), 6.99 (s, 1H), 5.90 (m, 1H), 5.25 (s, 2H), 5.20 (d, *J* =9.0, 2H), 4.03 (t, *J* =6.5, 2H), 3.10 (d, *J* =6.5, 2H), 2.43 (t, *J* =7.0, 2H), 2.29 (s, 3H), 1.83 (p, *J* =7.0, 2H), 1.50 (m, 4H), 1.37 (m, 8H); ^{13}C NMR ($CDCl_3$) δ 157.84, 153.03, 136.48, 134.81, 134.25, 131.94, 128.5, 128.1, 127.8, 126.9, 124.8, 118.6, 117.4, 110.6, 107.5, 105.8, 70.5, 67.9, 60.5, 57.0, 41.6, 29.3, 29.2, 29.1, 27.3, 26.9, 25.6; MS (m/z); calcd. for $C_{29}H_{37}BrNO_2$ 510.2008, found 510.14 $[M+H^+]$, 430.21 $[M-Br]^+$.

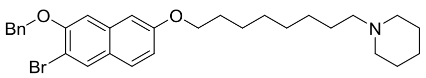

1-(8-((7-(benzyloxy)-6-bromonaphthalen-2-yl)oxy)octyl)piperidine (6b). Purification by fresh column chromatogranpy (silica, 5% MeOH in CH_2Cl_2) gave a white solid (28 mg, 54 %). ^1H NMR ($CDCl_3$) δ 7.98 (s, 1H), 7.57 (d, *J* =9.0, 1H), 7.54 (d, *J* =7.5, 2H), 7.42 (t, *J*=7.5, 2H), 7.34 (t, *J*=7.5, 1H), 7.12 (s, 1H), 7.02 (dd, *J* =2.5, 9.0, 1H), 6.99 (s, 1H), 5.25 (s, 2H), 4.03 (t, *J* =6.0, 2H), 2.40 (br, 4H), 2.30 (t, *J* =8.0, 2H), 1.83 (p, *J* =6.5, 2H), 1.61 (p, *J* = 5.5, 4H), 1.49 (m, 6H), 1.37 (m, 6H); ^{13}C NMR ($CDCl_3$) δ 157.8, 153.0, 136.5, 134.8, 134.2, 131.9, 128.5, 128.1, 127.8, 126.9, 124.8, 117.4, 110.6, 107.5, 105.8, 70.6, 67.9, 59.5, 54.6, 29.4, 29.2, 29.1, 27.6, 26.8, 26.0, 25.8, 24.0; MS (m/z); calcd. for $C_{30}H_{39}BrNO_2$ 524.2164, found 524.16 [$M+H^+$], 444.22 $[M\text{-}Br]^+$.

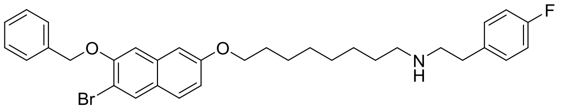

8-((7-(benzyloxy)-6-bromonaphthalen-2-yl)oxy)-N-(4-fluorophenethyl)octan-1-amine (6c). Purification by fresh column chromatogranpy (silica, 5% MeOH and 1% NH_4OH in CH_2Cl_2) gave a white solid (41 mg, 50 %). ^1H NMR ($CDCl_3$) δ 7.98 (s, 1H), 7.57 (d, *J* =9.0, 1H), 7.54 (d, *J* =7.5, 2H), 7.42 (t, *J*=7.5, 2H), 7.35 (t, *J*=7.5, 1H), 7.17 (m, 3H), 7.12 (s, 1H), 7.00 (m, 3H), 5.25 (s, 2H), 4.03 (t, *J* =6.5, 2H), 2.87 (t, *J* =7.0, 2H), 2.80 (t, *J* =7.0, 2H), 2.63 (t, *J* =7.5, 2H), 1.83 (p, *J* =6.5, 2H), 1.50 (m, 5H), 1.34 (m, 7H); ^{13}C NMR ($CDCl_3$) δ 162.3, 160.4, 157.8, 153.0, 136.4, 135.6, 135.5, 134.8, 131.9, 130.0, 129.9, 128.5, 128.1, 127.8, 126.9, 124.8, 117.4, 115.2, 115.0, 110.6, 107.5, 105.8, 70.5, 67.9, 51.2, 49.8, 35.4, 29.8, 29.4, 29.2, 29.1, 27.2, 25.9, 25.8, 24.0; MS (m/z); calcd. for $C_{33}H_{38}BrFNO_2$ 578.2070, found 578.16 [$M+H^+$], 580.14 [$M+H^++2$].

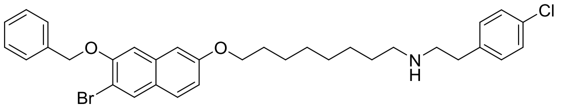

8-((7-(benzyloxy)-6-bromonaphthalen-2-yl)oxy)-N-(4-chlorophenethyl)octan-1-amine (6d). Purification by fresh column chromatogranpy (silica, 5% MeOH and 1% NH_4OH in CH_2Cl_2) gave a colorless oil (70 mg, 69%), ^1H NMR ($CDCl_3$) δ 7.95 (s, 1H), 7.53 (m, 3H), 7.41 (m, 4H), 7.32 (t, *J*=7.0, 1H), 7.07 (m, 3H), 6.99 (dd, *J* = 2.0, 9.0, 1H), 6.97 (s, 1H), 5.22 (s, 2H), 4.01 (t, *J* =6.5, 2H), 2.83 (t, *J* =7.0, 2H), 2.74 (t, *J* =7.0, 2H), 2.59 (t, *J* =7.0, 2H), 1.81 (p, *J* = 7.0, 15, 2H),

1.45 (m, 4H), 1.31 (m, 6H); ^{13}C NMR ($CDCl_3$) δ 157.8, 153.0, 139.0, 136.4, 134.8, 131.9, 131.4, 130.3, 128.5, 128.1, 127.8, 126.9, 124.8, 119.8, 117.4, 110.6, 107.5, 105.8, 70.5, 67.9, 50.9, 49.8, 35.8, 30.0, 29.4, 29.2, 29.1, 27.2, 25.9; MS (m/z); calcd. for $C_{33}H_{37}BrClNO_2$ 593.1696 $[M]^+$, found 514.19 $[M-Br]^+$.

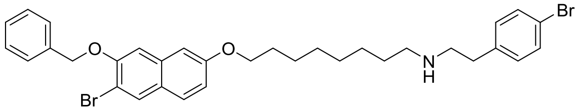

8-((7-(benzyloxy)-6-bromonaphthalen-2-yl)oxy)-N-(4-bromophenethyl)octan-1-amine (6e). Purification by fresh column chromatogranpy (silica, 5% MeOH and 1% NH_4OH in CH_2Cl_2) gave a white solid (70 mg, 79 %), ^1H NMR ($CDCl_3$) δ 7.96 (s, 1H), 7.52 (m, 3H), 7.39 (t, *J* = 7.5, 2H), 7.31 (t, *J*=7.0, 1H), 7.23 (d, *J* =8.5, 2H), 7.11 (d, *J* =8.5, 2H), 7.07 (s, 1H), 6.99 (dd, *J* = 2.0, 9.0, 1H), 6.96 (s, 1H), 5.20 (s, 2H), 3.99 (t, *J* =6.5, 2H), 2.82 (t, *J* =7.0, 2H), 2.74 (t, *J* =7.0, 2H), 2.58 (t, *J* =7.0, 2H), 1.80 (p, *J* = 7.0, 15, 2H), 1.44 (m, 4H), 1.33 (m, 6H); ^{13}C NMR ($CDCl_3$) δ 157.7, 152.9, 138.5, 136.4, 134.7, 131.8, 131.7, 129.9, 128.5, 128.4, 128.0, 127.8, 126.8, 124.8, 117.3, 110.5, 107.4, 105.7, 70.5, 67.9, 50.9, 49.8, 35.6, 29.9, 29.3, 29.2, 29.1, 27.2, 25.9; MS $[M+H^+]$; calcd. for $C_{33}H_{38}Br_2NO_2$ 638.1269, found 638.08.

5. Synthesis of 7a-7d

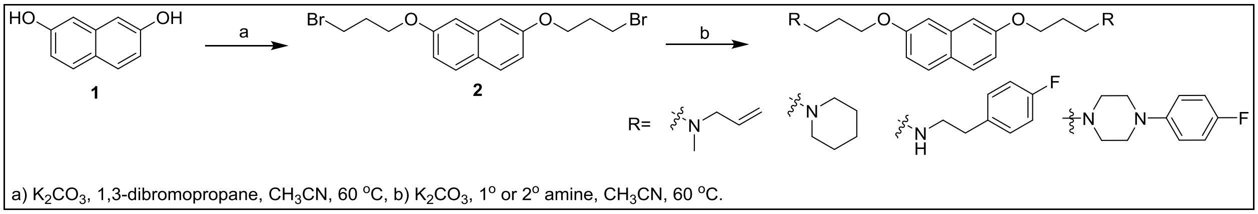

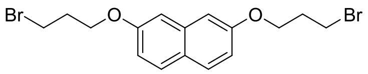

2,7-bis(3-bromopropoxy)naphthalene (2). A solution of 1,3-dibromopropane (0.1 mol), 2,7-dihydroxynaphthalene (0.01 mol) and K_2CO_3 (0.05 mol) in acetonitrile 50 mL was heated to reflux and stirred for 5 h. The mixture was cooled and filtered and the solvent was removed under reduced vacuum. The residue was dissolved in ethyl acetate and washed with water and brine. The organic layer was dried over MgSO4. Fresh column chromatography (silica gel, ethyl acetate:hexane = 1:10) and recrystallization (ethyl acetate and hexane) gave white solid (2.8g, 69%). ^1H NMR ($CDCl_3$) δ 7.68 (d, *J* = 9.0, 2H), 7.09 (d, *J* =2.5, 2H), 7.01 (dd, *J* =2.5, 9.0, 2H),

4.22 (t, *J* =6, 4H), 3.66 (t, *J* =6.5, 4H), 2.39 (p, *J* =6.0, 4H); ^{13}C NMR ($CDCl_3$) δ 157.20, 135.76, 129.12, 124.41, 116.19, 106.18, 65.24, 32.30, 30.04; MS [M+H$^+$]; calcd. for $C_{16}H_{19}Br_2O_2$ 400.9752, found 401.91.

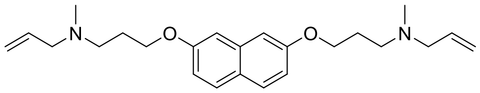

N,N'-((naphthalene-2,7-diylbis(oxy))bis(propane-3,1-diyl))bis(N-methylprop-2-en-1-amine) (7a). Purification by fresh column chromatogranpy (silica, MeOH : NH_4OH : CH_2Cl_2 = 9:1:100) gave a colorless oil (66 mg, 71 %). ^{1}H NMR ($CDCl_3$) δ 7.64 (d, *J* = 9.0, 2H), 7.05 (d, *J* =1.5, 2H), 7.00 (dd, *J* =2.0, 9.0, 2H), 5.88 (m, 2H), 5.17 (m, 4H), 4.12 (t, *J* = 6.5, 4H), 3.04 (d, *J* =6.5, 4H), 2.57 (t, *J* = 7.0, 4H), 2.27 (s, 6H), 2.02 (p, *J* = 6.5, 15, 4H); ^{13}C NMR ($CDCl_3$) δ 157.4, 135.8, 135.7, 128.9, 124.1, 117.3, 116.1, 106.0, 66.1, 60.9, 53.7, 42.0, 27.2; MS (m/z); calcd. for $C_{24}H_{35}N_2O_2$ 383.2699, found 383.21 [M+H$^+$], 405.19 [M+Na]$^+$.

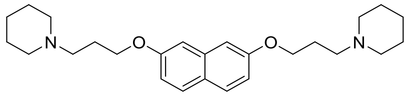

2,7-bis(3-(piperidin-1-yl)propoxy)naphthalene (7b). Purification by fresh column chromatogranpy (silica, MeOH : NH_4OH : CH_2Cl_2 = 9:1:100) gave a white solid (75 mg, 73.5 %). ^{1}H NMR ($CDCl_3$) δ 7.63 (d, *J* = 9.0, 2H), 7.04 (d, *J* =1.5, 2H), 6.98 (dd, *J* =2.5, 9.0, 2H), 4.11 (t, *J* = 6.5, 4H), 2.51 (t, *J* = 6.5, 4H), 2.50 (br, 8H), 2.04 (p, *J* = 6.5, 15, 4H), 1.61 (p, *J* = 5.5, 11, 8H), 1.45 (br, 4H); ^{13}C NMR ($CDCl_3$) δ 157.5, 135.9, 128.9, 124.1, 116.2, 106.0, 66.4, 56.0, 54.6, 26.8, 26.0, 24.4; MS [M+H$^+$]; calcd. for $C_{26}H_{39}N_2O_2$ 411.3012, found 411.20.

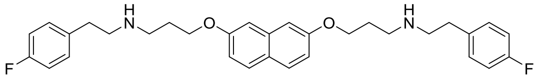

3,3'-(naphthalene-2,7-diylbis(oxy))bis(N-(4-fluorophenethyl)propan-1-amine) (7c). Purification by fresh column chromatogranpy (silica, MeOH : NH_4OH : CH_2Cl_2 = 9:1:100) gave a colorless oil (70 mg, 56 %). ^{1}H NMR ($CDCl_3$) δ 7.63 (d, *J* = 9.0, 2H), 7.16 (d, *J* = 6.0, 2H), 7.15 (d, *J* = 5.5, 2H), 6.95 (m, 8H), 3.96 (t, *J* = 6.0, 4H), 2.89 (t, *J* = 7.0, 4H), 2.85 (t, *J* = 7.0, 4H), 2.79 (t, *J* = 7.0, 4H), 2.00 (p, *J* = 6.50, 4H); ^{13}C NMR ($CDCl_3$) δ 162.3, 160.4, 157.4, 135.8, 135.67, 135.64, 130.0, 129.9, 129.0, 124.1, 116.1, 115.2, 115.0, 106.0, 66.2, 51.1, 46.8, 35.5, 29.6; MS [M+H$^+$]; calcd. for $C_{32}H_{37}F_2N_2O_2$ 519.2823, found 519.22.

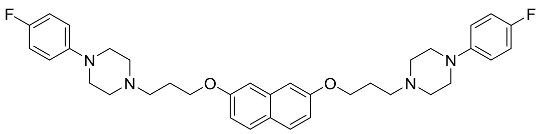

2,7-bis(3-(4-(4-fluorophenyl)piperazin-1-yl)propoxy)naphthalene (7d). White solid (110 mg, 76.3 %). ^{1}H NMR ($CDCl_3$) δ 7.65 (d, *J* = 9.0, 2H), 7.05 (s, 2H), 6.98 (m, 6H), 6.88 (d, *J* = 4.5, 2H), 6.87 (d, *J* = 4.5, 2H), 4.14 (t, *J* = 6.0, 4H), 3.14 (t, J = 4.5, 8H), 2.64 (m, 12H), 2.07 (p, *J* = 6.0, 4H); ^{13}C NMR ($CDCl_3$) δ 158.0, 157.5, 156.1, 147.9, 135.9, 129.0, 124.2, 117.7, 117.6, 116.2, 115.5, 115.3, 106.0, 66.1, 55.1, 53.2, 50.1; MS [M+H$^+$]; calcd. for $C_{36}H_{43}F_2N_4O_2$ 601.3354, found 601.27.

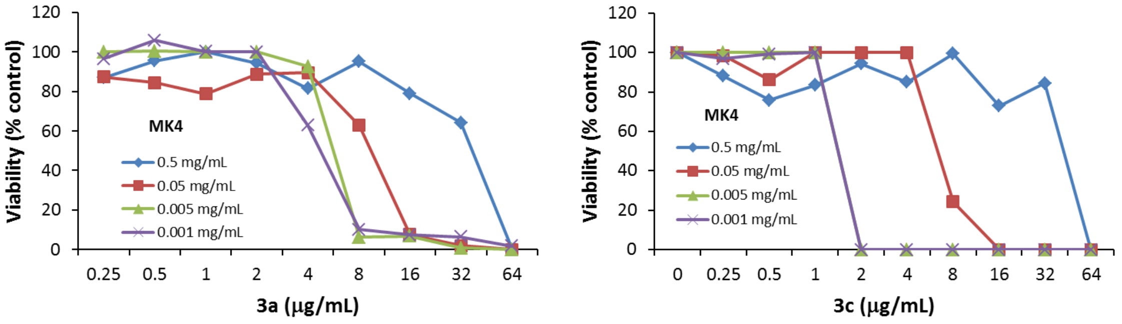

Figure **S1**. Menaquinone rescue experiments. MRSA (USA 700 for **3a**, USA 200 for **3c**) was treated with various concentrations of menaquinone (MK-4) in the presence of inhibitor.

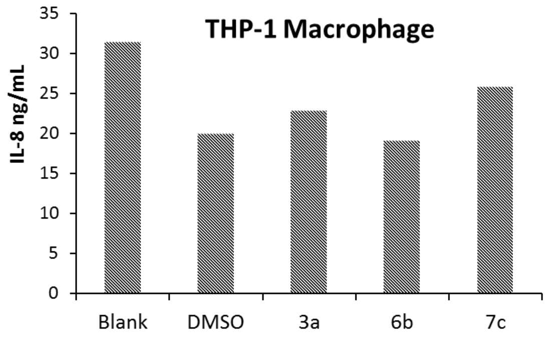

Figure **S2**. IL-8 released from macrophages treated with inhibitors. THP-1 macrophages were treated with 3a, 6b or 7c at concentration of their MIC for 24 h at 37 °C. Culture supernatants were analyzed for cytokines. Data represents the mean ± SEM of triplicate (n = 3). Blank: No inhibitor was added. DMSO (<0.05%).

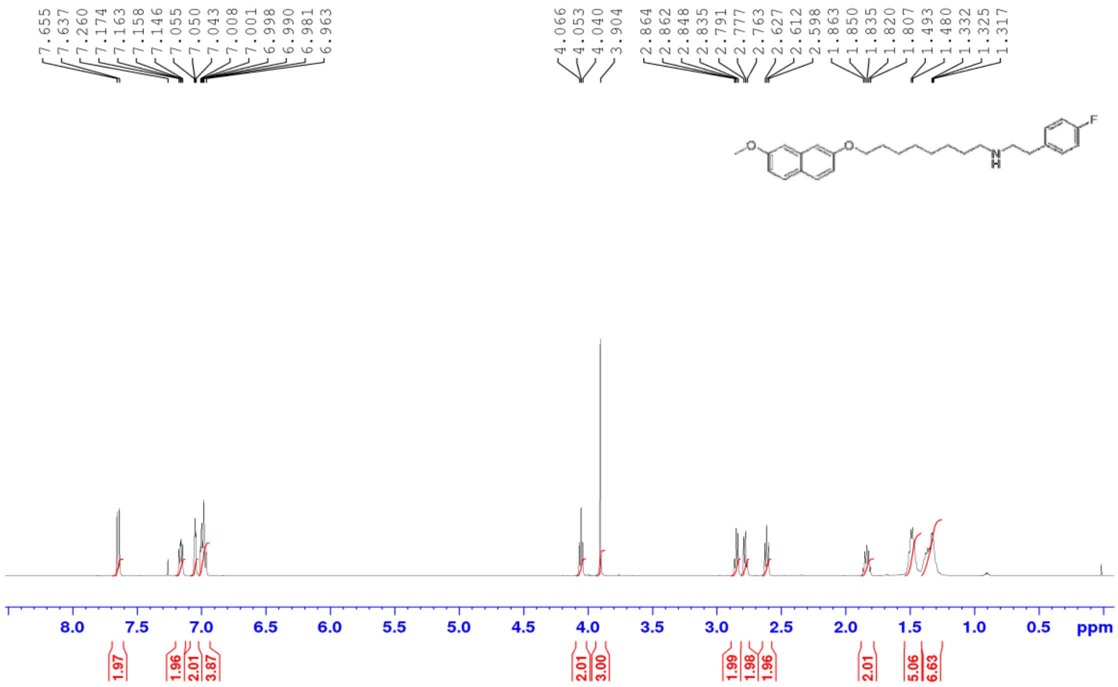

7.655
7.637
7.260
7.174
7.163
7.158
7.146
7.055
7.050
7.043
7.008
7.001
6.998
6.990
6.981
6.963
4.066
4.053
4.040
3.904
2.864
2.862
2.848
2.835
2.791
2.777
2.763
2.627
2.612
2.598
1.863
1.850
1.835
1.820
1.807
1.493
1.480
1.332
1.325
1.317
F
O
O
N
H
8.0 7.5 7.0 6.5 6.0 5.5 5.0 4.5 4.0 3.5 3.0 2.5 2.0 1.5 1.0 0.5 ppm
1.97
1.96
2.01
3.87
2.01
3.00
1.99
1.98
1.96
2.01
5.06
6.63

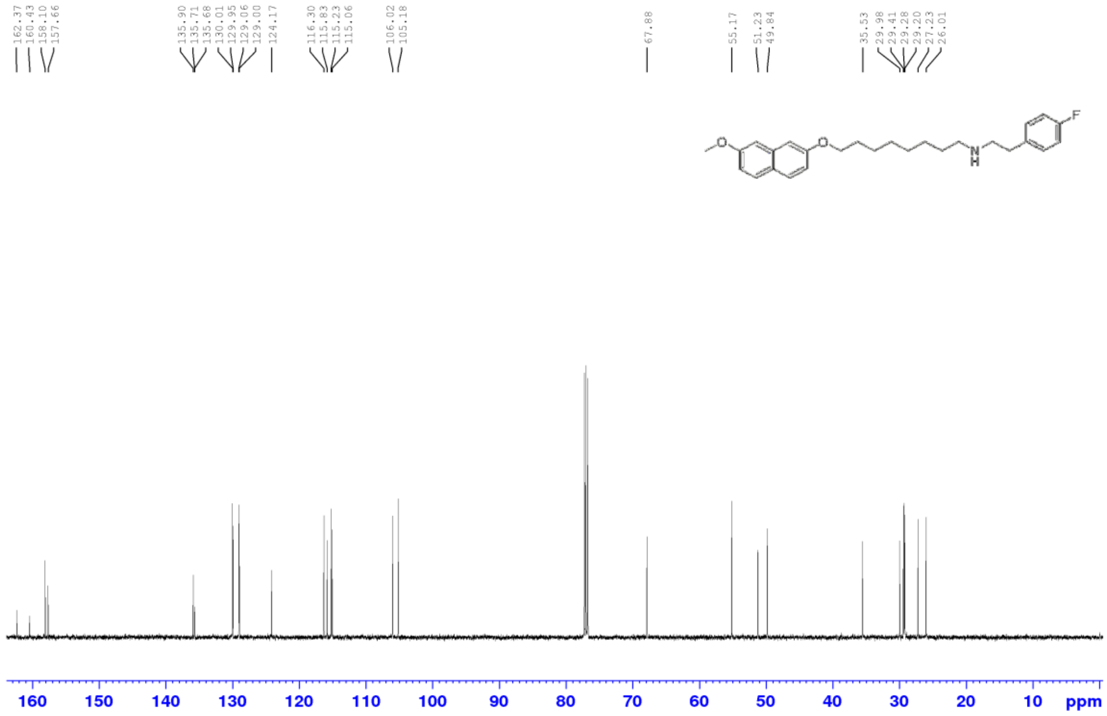

162.37
160.43
158.10
157.66
135.90
135.71
135.68
130.01
129.95
129.06
129.00
124.17
116.30
115.83
115.23
115.06
106.02
105.18
67.88
55.17
51.23
49.84
35.53
29.98
29.41
29.28
29.20
27.23
26.01
F
O
O
N
H
160 150 140 130 120 110 100 90 80 70 60 50 40 30 20 10 ppm

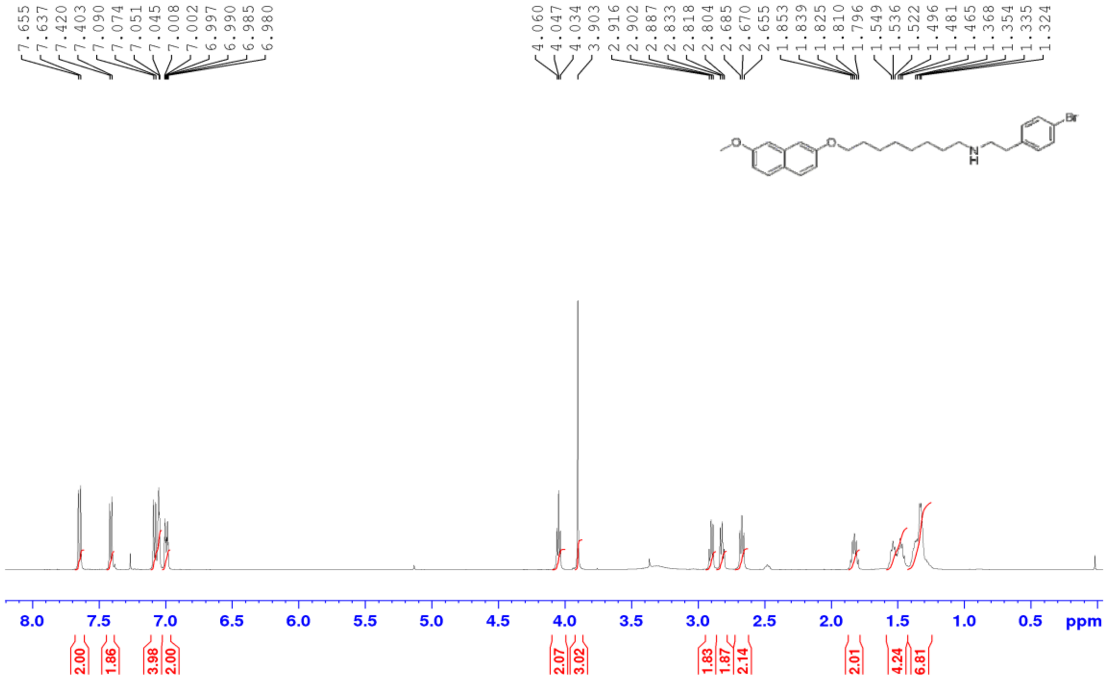

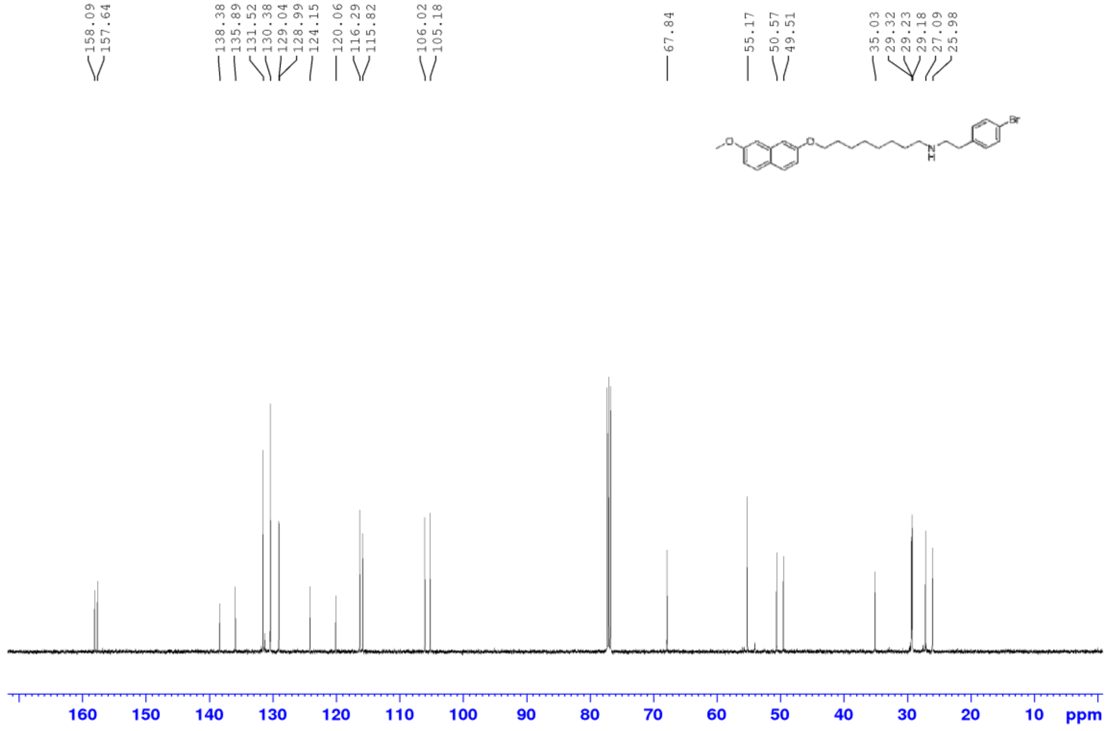

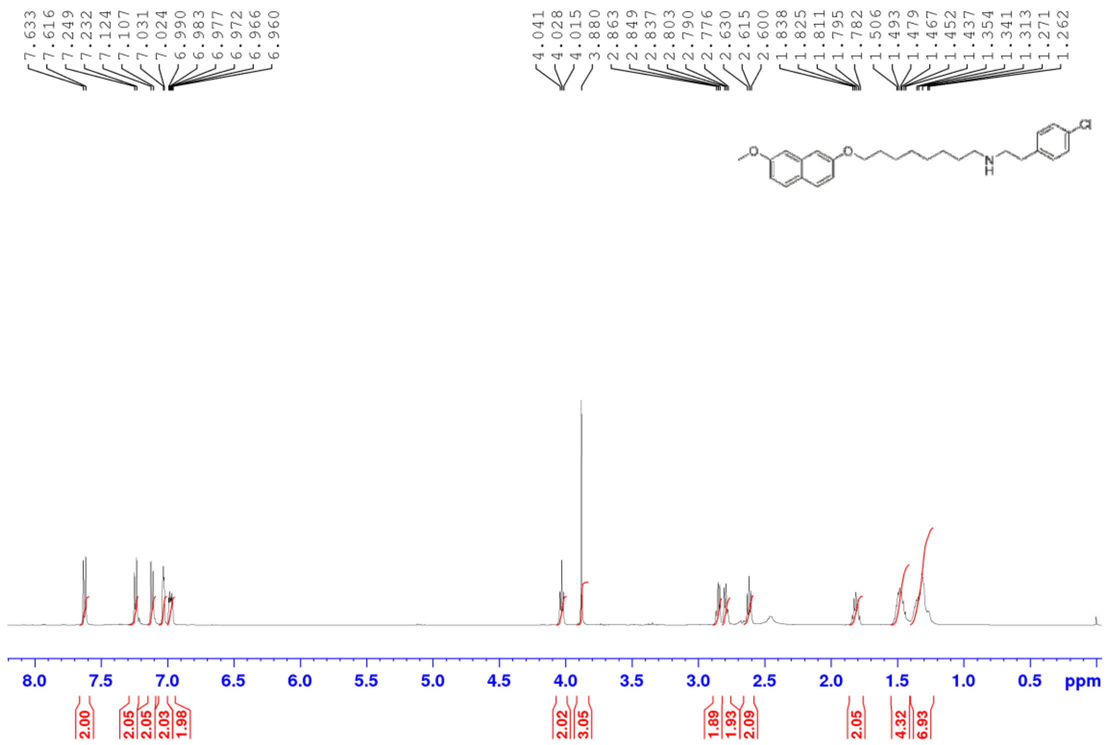

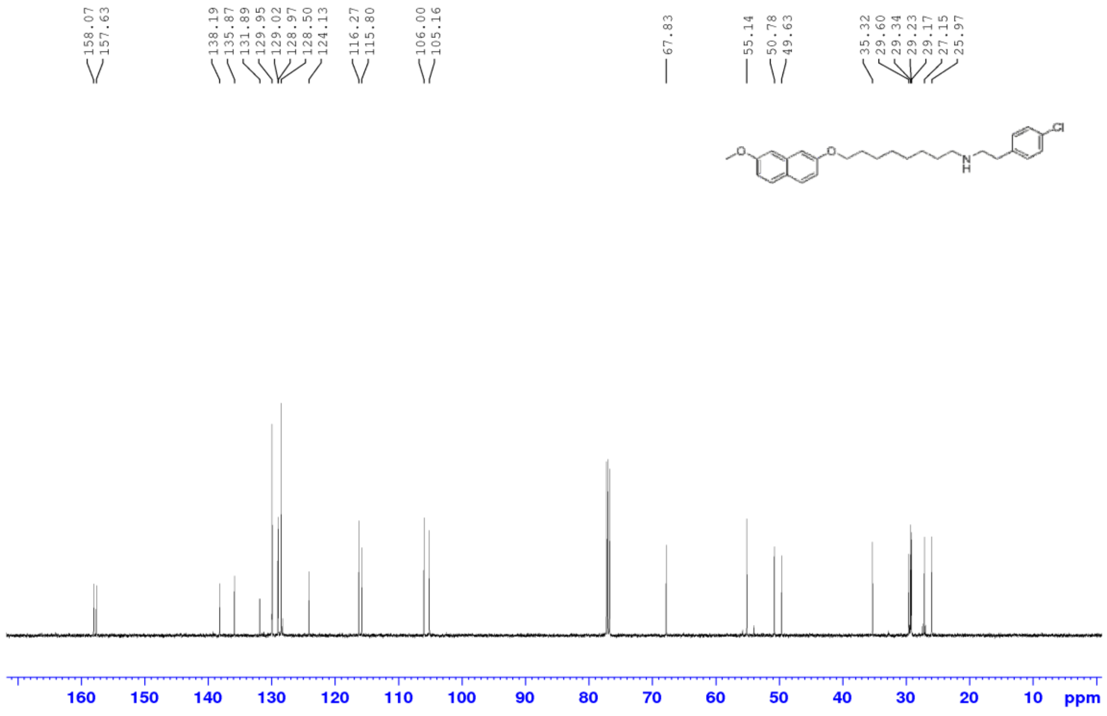

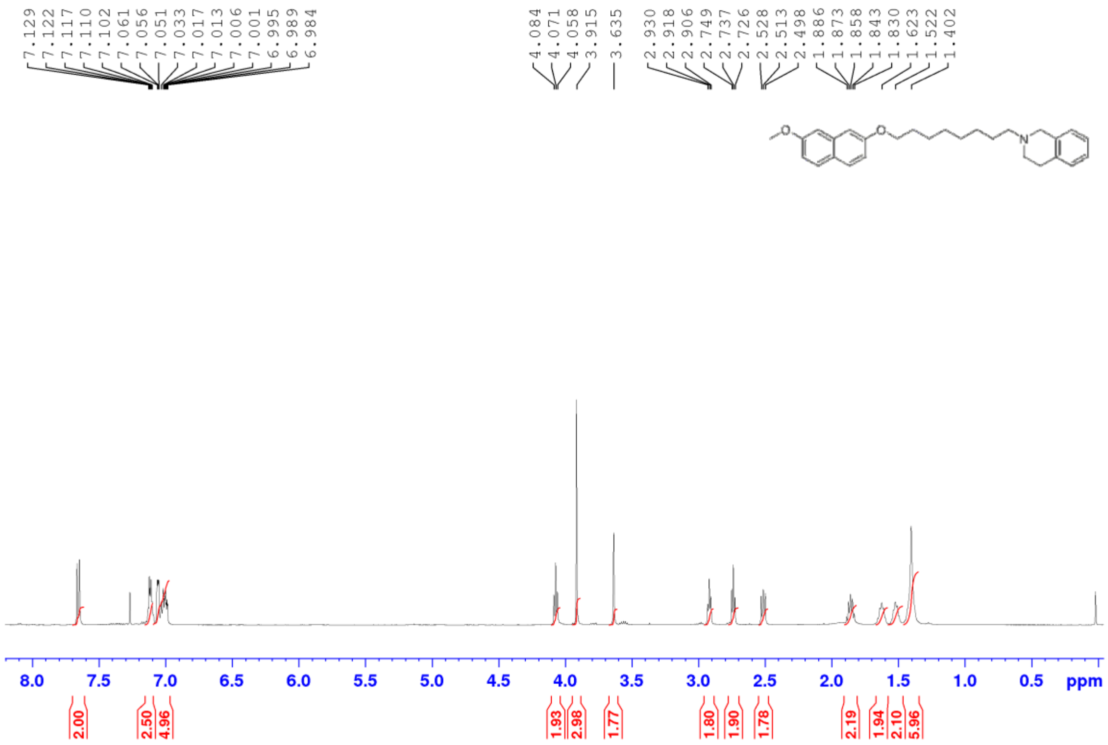
7.129
7.122
7.117
7.110
7.102
7.061
7.056
7.051
7.033
7.017
7.013
7.006
7.001
6.995
6.989
6.984
4.084
4.071
4.058
3.915
3.635
2.930
2.918
2.906
2.749
2.737
2.726
2.528
2.513
2.498
1.886
1.873
1.858
1.843
1.830
1.623
1.522
1.402
O
O
N
8.0
7.5
7.0
6.5
6.0
5.5
5.0
4.5
4.0
3.5
3.0
2.5
2.0
1.5
1.0
0.5
ppm
2.00
2.50
4.96
1.93
2.98
1.77
1.80
1.90
1.78
2.19
1.94
2.10
5.96

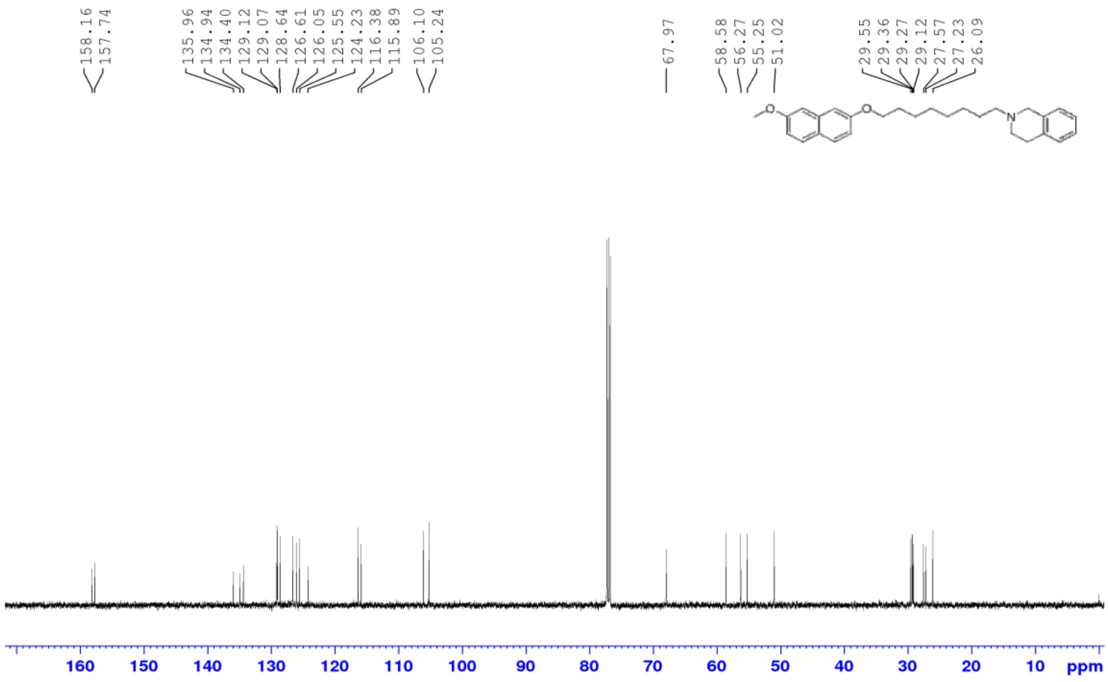
158.16
157.74
135.96
134.94
134.40
129.12
129.07
128.64
126.61
126.05
125.55
124.23
116.38
115.89
106.10
105.24
67.97
58.58
56.27
55.25
51.02
29.55
29.36
29.27
29.12
27.57
27.23
26.09
O
O
N
160
150
140
130
120
110
100
90
80
70
60
50
40
30
20
10
ppm

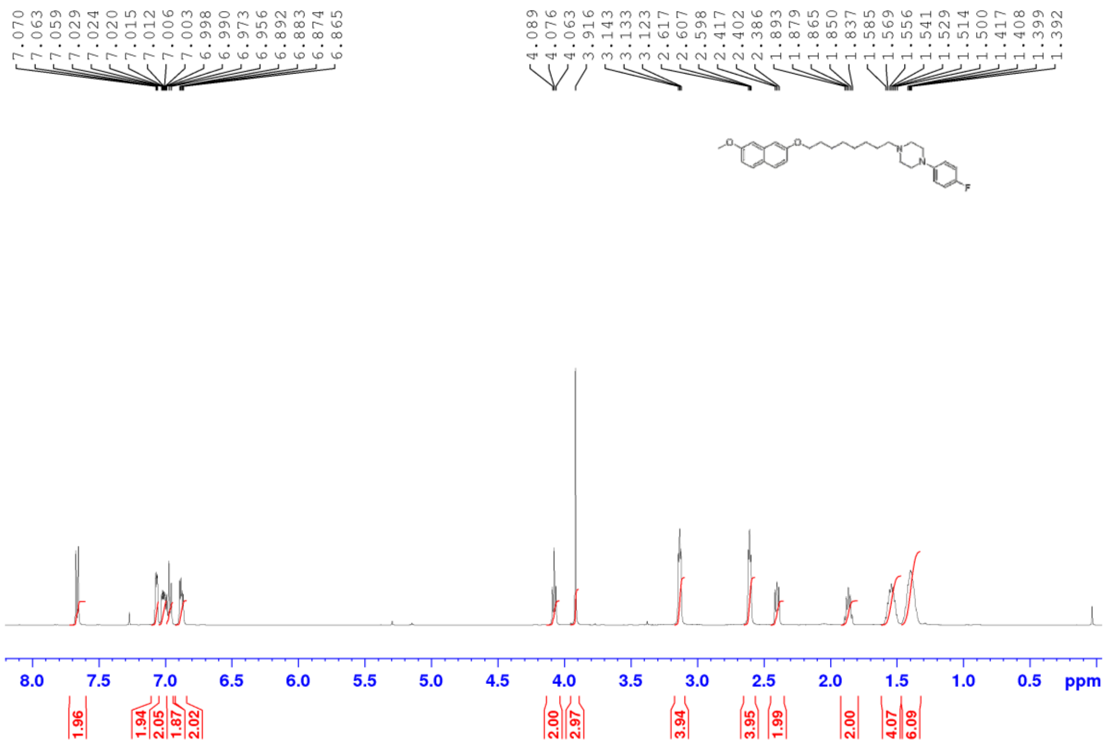
7.070
7.063
7.059
7.029
7.024
7.020
7.015
7.012
7.006
7.003
6.998
6.990
6.973
6.956
6.892
6.883
6.874
6.865
4.089
4.076
4.063
3.916
3.143
3.133
3.123
2.617
2.607
2.598
2.417
2.402
2.386
1.893
1.879
1.865
1.850
1.837
1.585
1.569
1.556
1.541
1.529
1.514
1.500
1.417
1.408
1.399
1.392
F
N
O
1.96
1.94
2.05
1.87
2.02
2.00
2.97
3.94
3.95
1.99
2.00
4.07
6.09
ppm

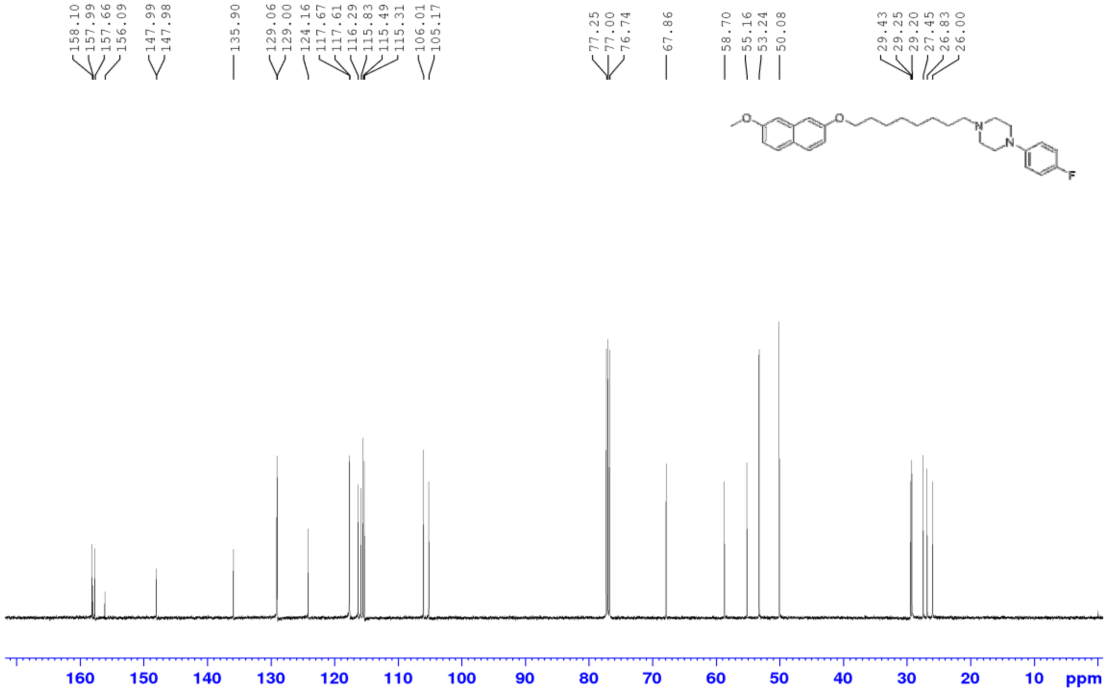
158.10
157.99
157.66
156.09
147.99
147.98
135.90
129.06
129.00
124.16
117.67
117.61
116.29
115.83
115.49
115.31
106.01
105.17
77.25
77.00
76.74
67.86
58.70
55.16
53.24
50.08
29.43
29.25
29.20
27.45
26.83
26.00
F
N
O
ppm

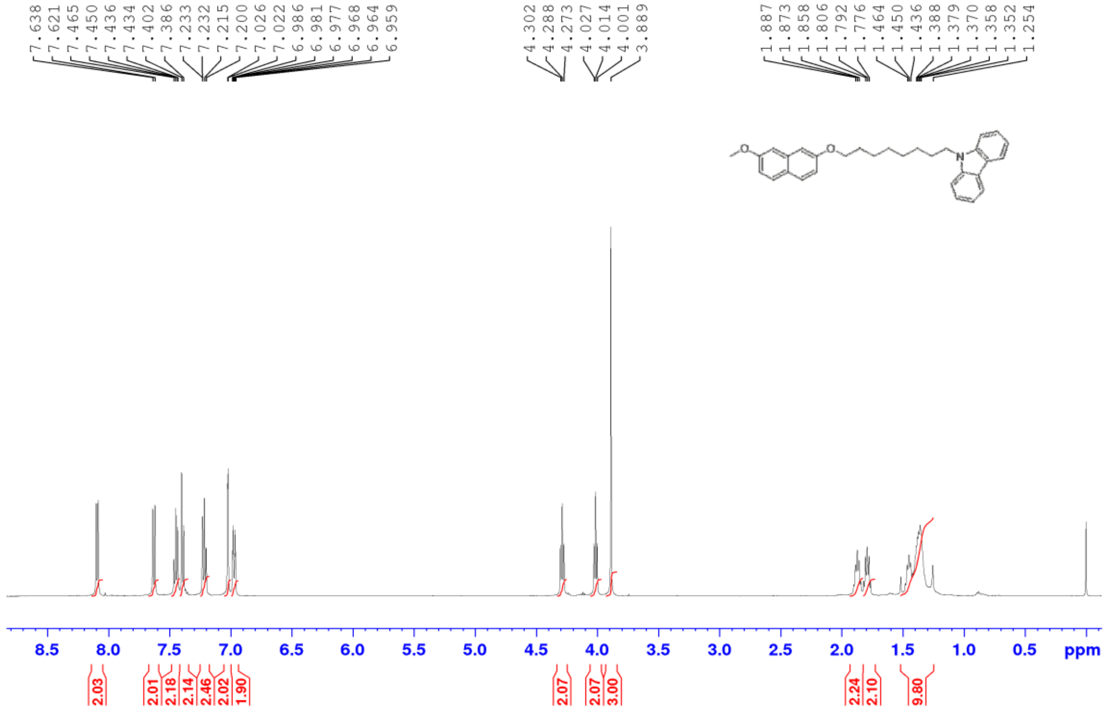

7.638
7.621
7.465
7.450
7.436
7.434
7.402
7.386
7.233
7.232
7.215
7.200
7.026
7.022
6.986
6.981
6.977
6.968
6.964
6.959
4.302
4.288
4.273
4.027
4.014
4.001
3.889
1.887
1.873
1.858
1.806
1.792
1.776
1.464
1.450
1.436
1.388
1.379
1.370
1.358
1.352
1.254
O
N
8.5
8.0
7.5
7.0
6.5
6.0
5.5
5.0
4.5
4.0
3.5
3.0
2.5
2.0
1.5
1.0
0.5
ppm
2.03
2.01
2.18
2.14
2.46
2.02
1.90
2.07
2.07
3.00
2.24
2.10
9.80

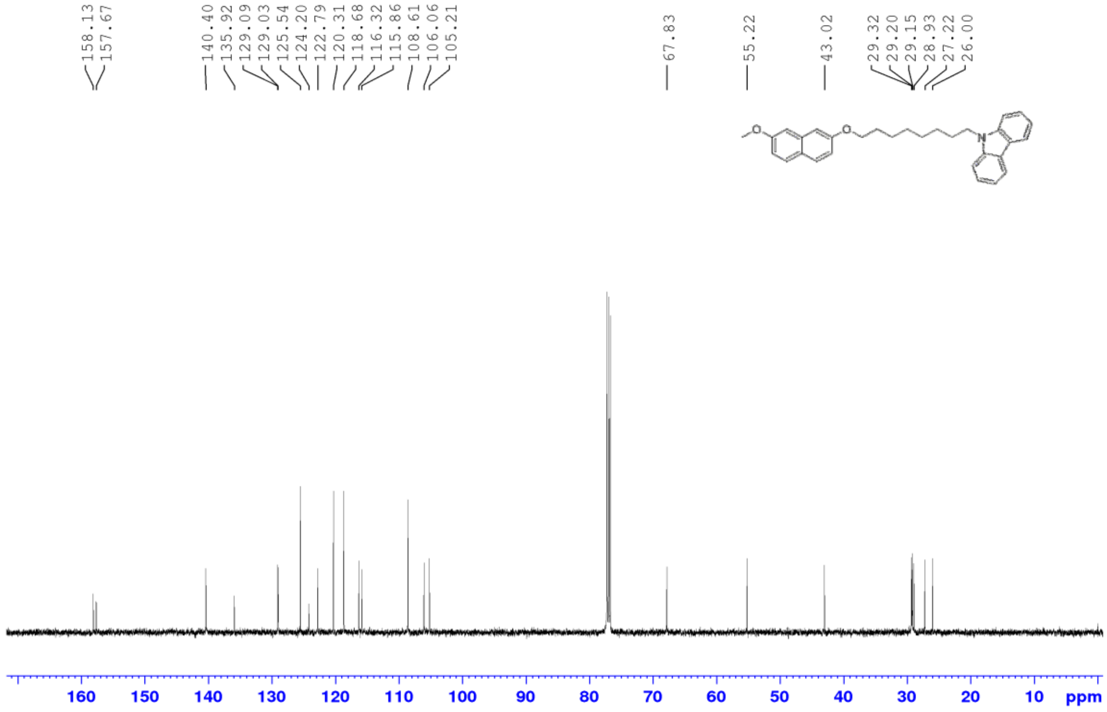

158.13
157.67
140.40
135.92
129.09
129.03
125.54
124.20
122.79
120.31
118.68
116.32
115.86
108.61
106.06
105.21
67.83
55.22
43.02
29.32
29.20
29.15
28.93
27.22
26.00
O
N
160
150
140
130
120
110
100
90
80
70
60
50
40
30
20
10
ppm

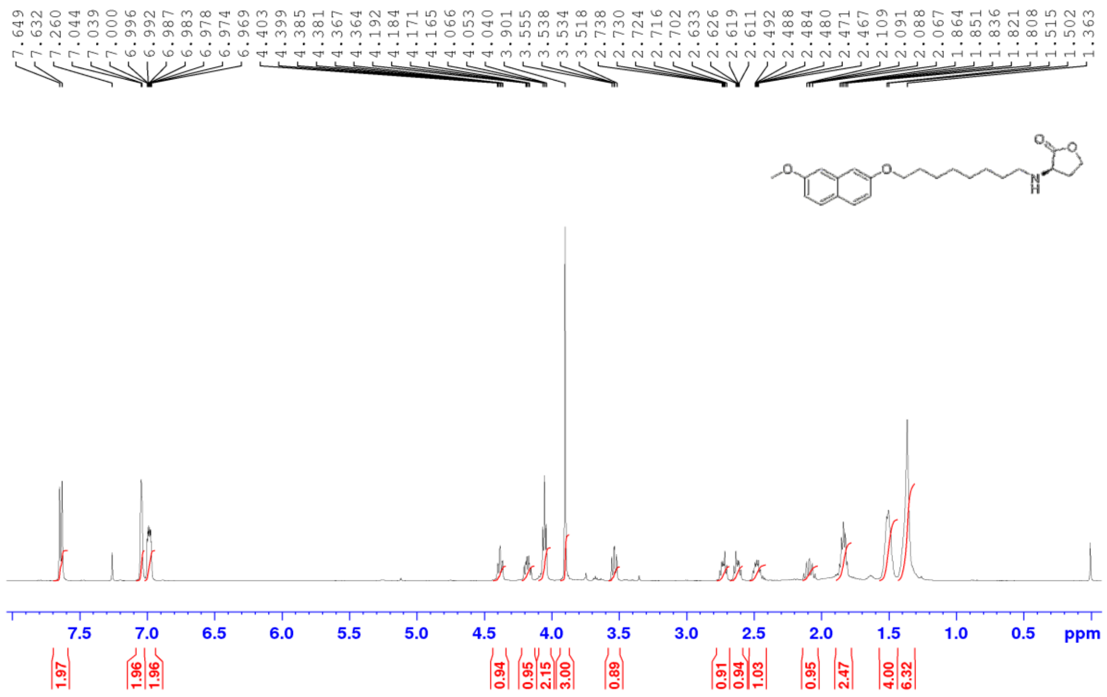

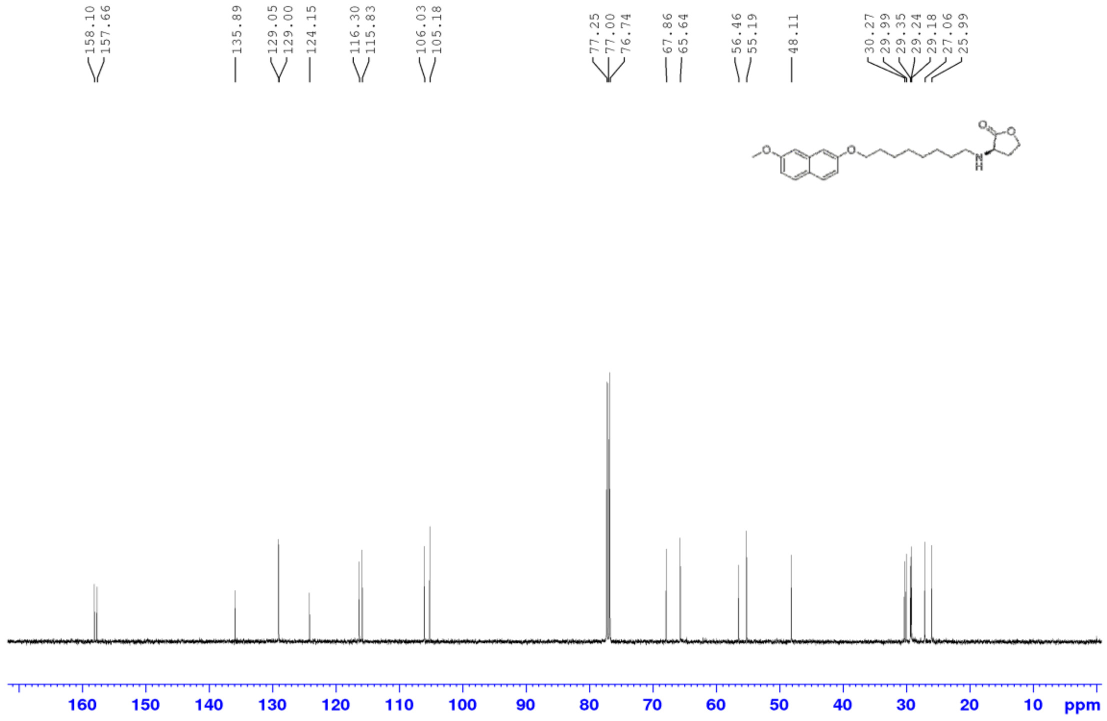

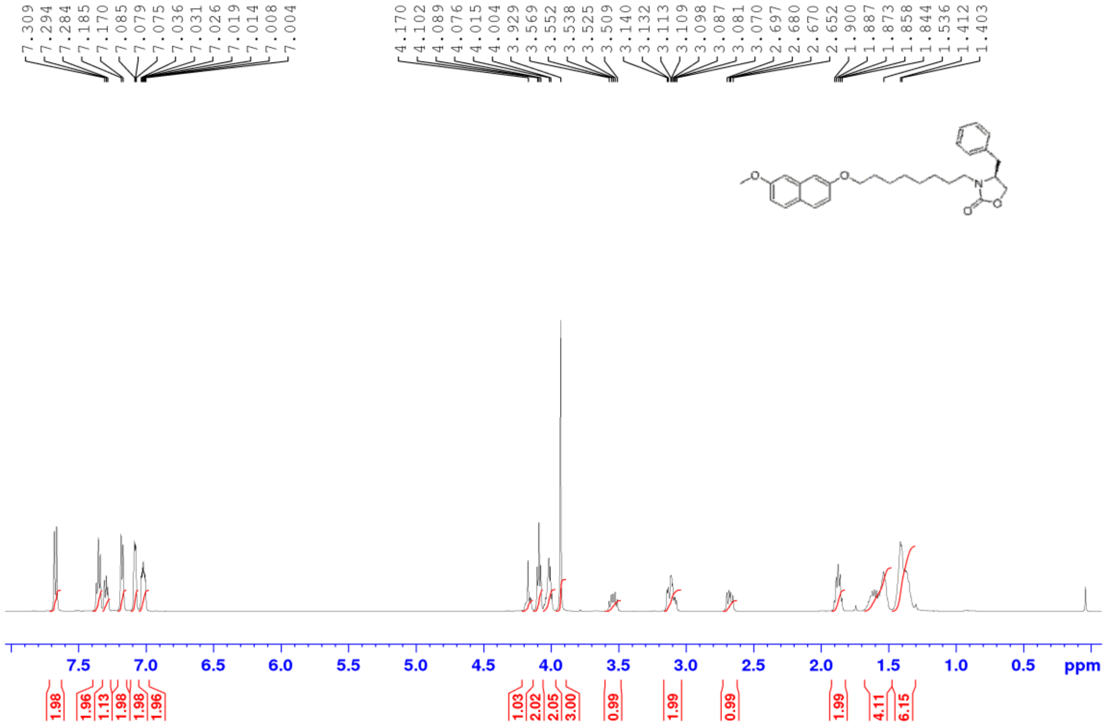
7.309
7.294
7.284
7.185
7.170
7.085
7.079
7.075
7.036
7.031
7.026
7.019
7.014
7.008
7.004
4.170
4.102
4.089
4.076
4.015
4.004
3.929
3.569
3.552
3.538
3.525
3.509
3.140
3.132
3.113
3.109
3.098
3.087
3.081
3.070
2.697
2.680
2.670
2.652
1.900
1.887
1.873
1.858
1.844
1.536
1.412
1.403
ppm
1.98
1.96
1.13
1.98
1.98
1.96
1.03
2.02
2.05
3.00
0.99
1.99
0.99
1.99
4.11
6.15

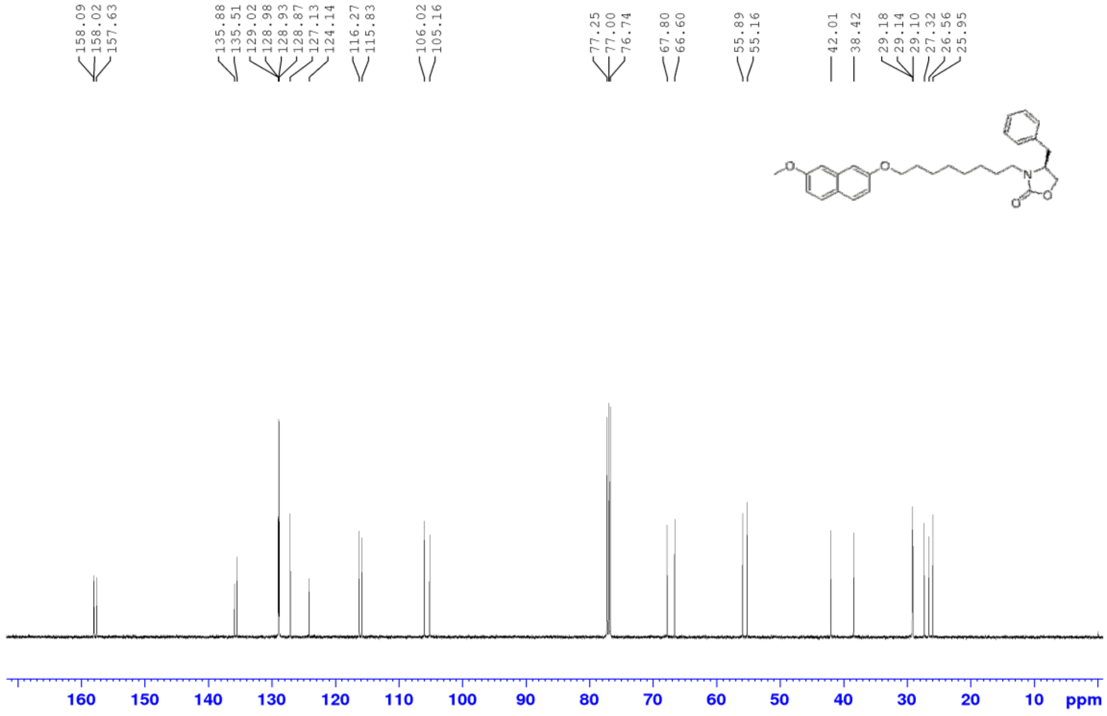
158.09
158.02
157.63
135.88
135.51
129.02
128.98
128.93
128.87
127.13
124.14
116.27
115.83
106.02
105.16
77.25
77.00
76.74
67.80
66.60
55.89
55.16
42.01
38.42
29.18
29.14
29.10
27.32
26.56
25.95
ppm

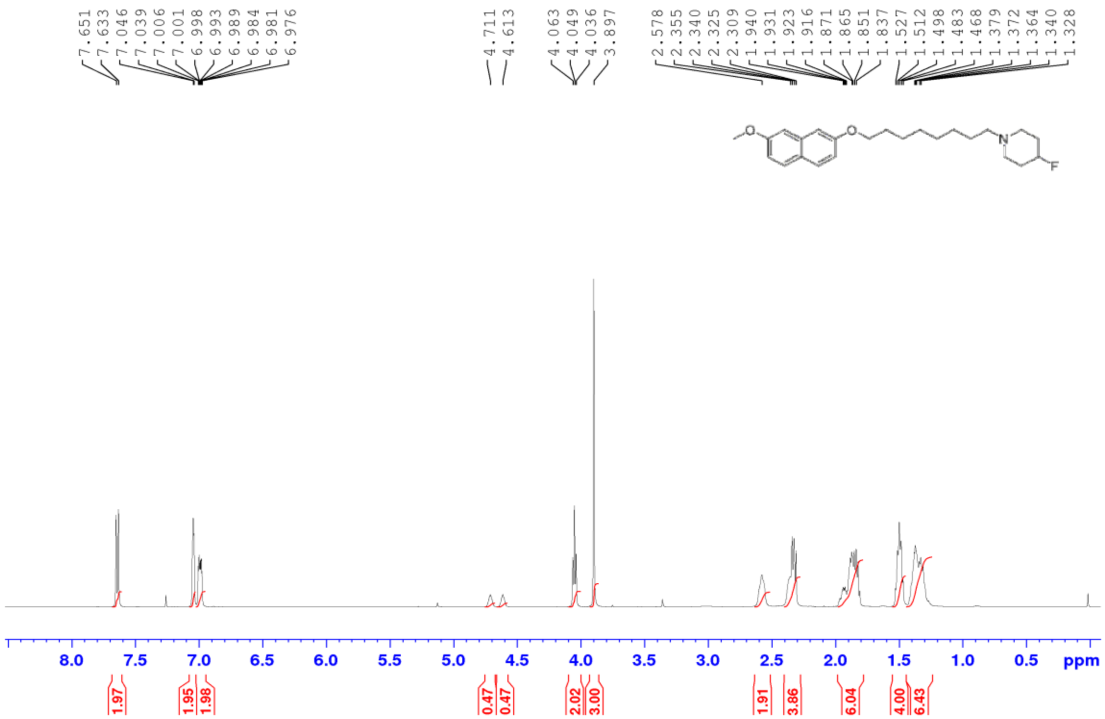

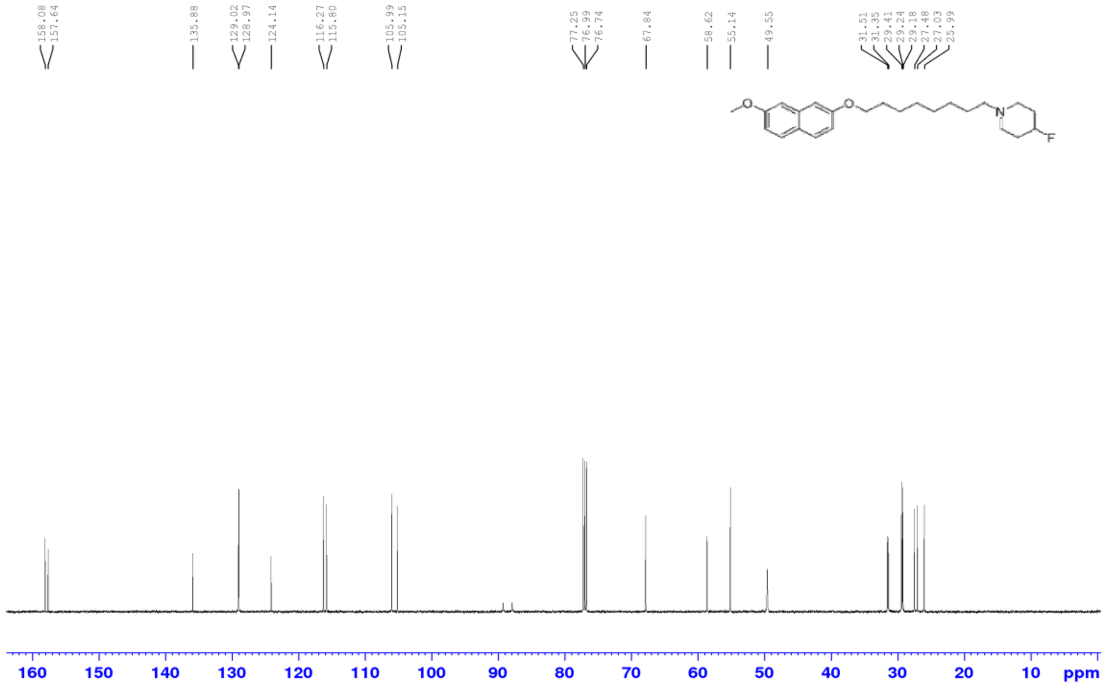

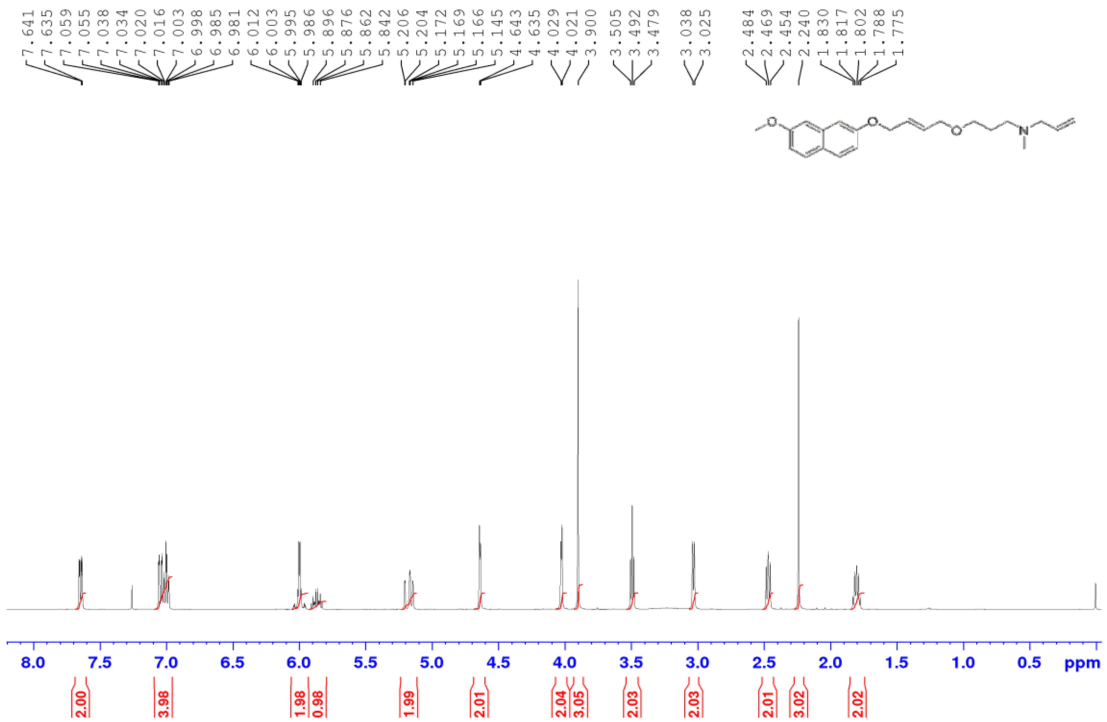

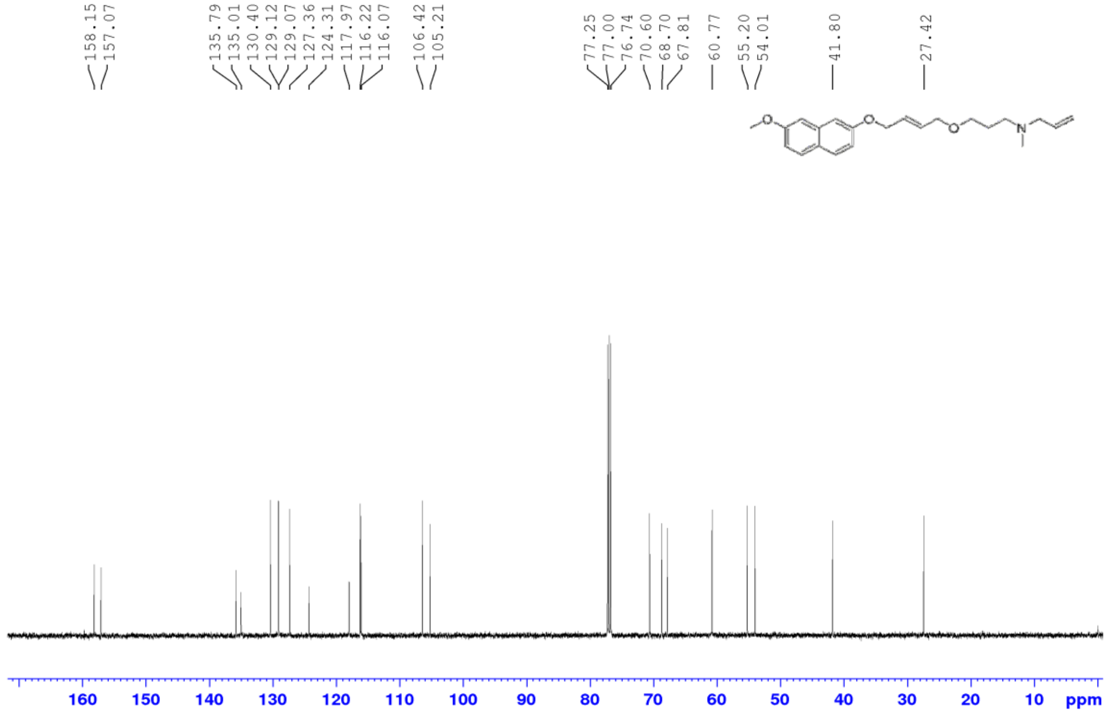

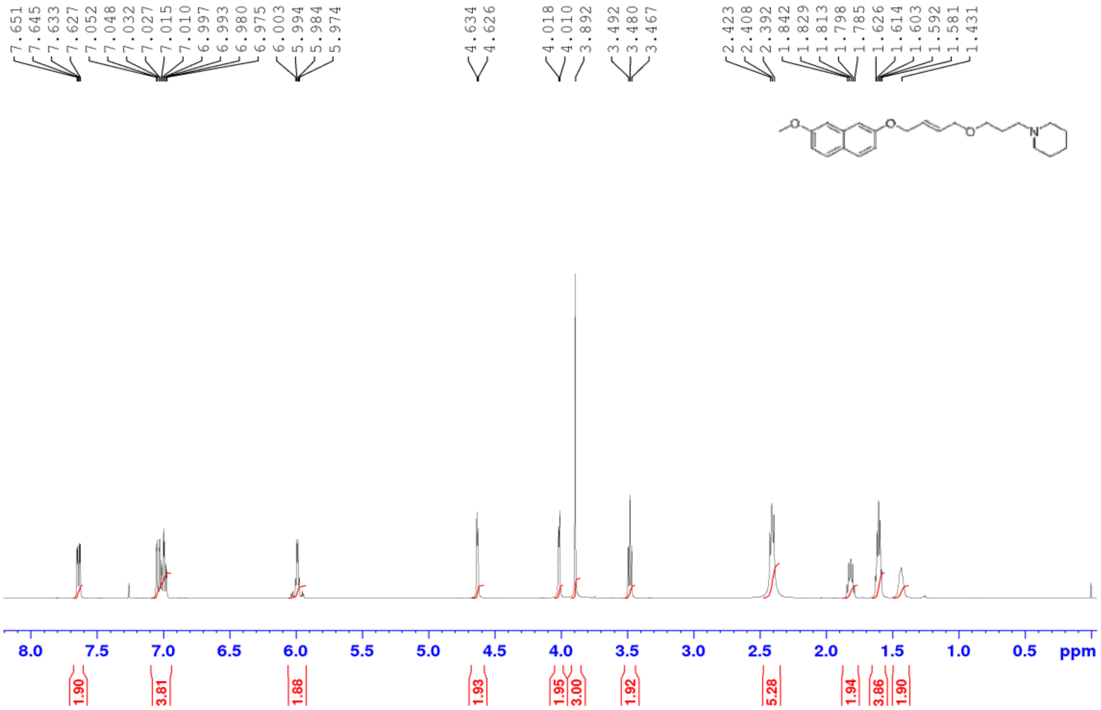
7.651 7.645 7.633 7.627 7.052 7.048 7.032 7.027 7.015 7.010 6.997 6.993 6.980 6.975 6.003 5.994 5.984 5.974
4.634 4.626
4.018 4.010 3.892
3.492 3.480 3.467
2.423 2.408 2.392 1.842 1.829 1.813 1.798 1.785 1.626 1.614 1.603 1.592 1.581 1.431
O
O
N
8.0 7.5 7.0 6.5 6.0 5.5 5.0 4.5 4.0 3.5 3.0 2.5 2.0 1.5 1.0 0.5 ppm
1.90 3.81 1.88 1.93 1.95 3.00 1.92 5.28 1.94 3.86 1.90

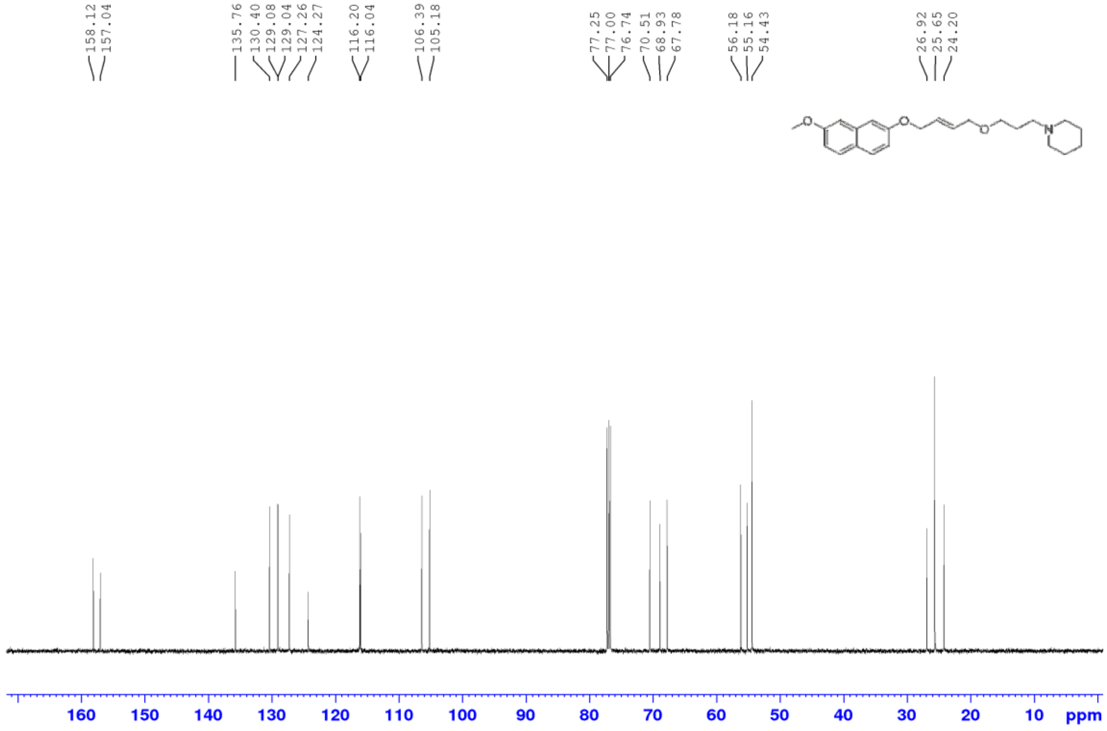
158.12 157.04
135.76 130.40 129.08 129.04 127.26 124.27
116.20 116.04
106.39 105.18
77.25 77.00 76.74 70.51 68.93 67.78
56.18 55.16 54.43
26.92 25.65 24.20
O
O
N
160 150 140 130 120 110 100 90 80 70 60 50 40 30 20 10 ppm

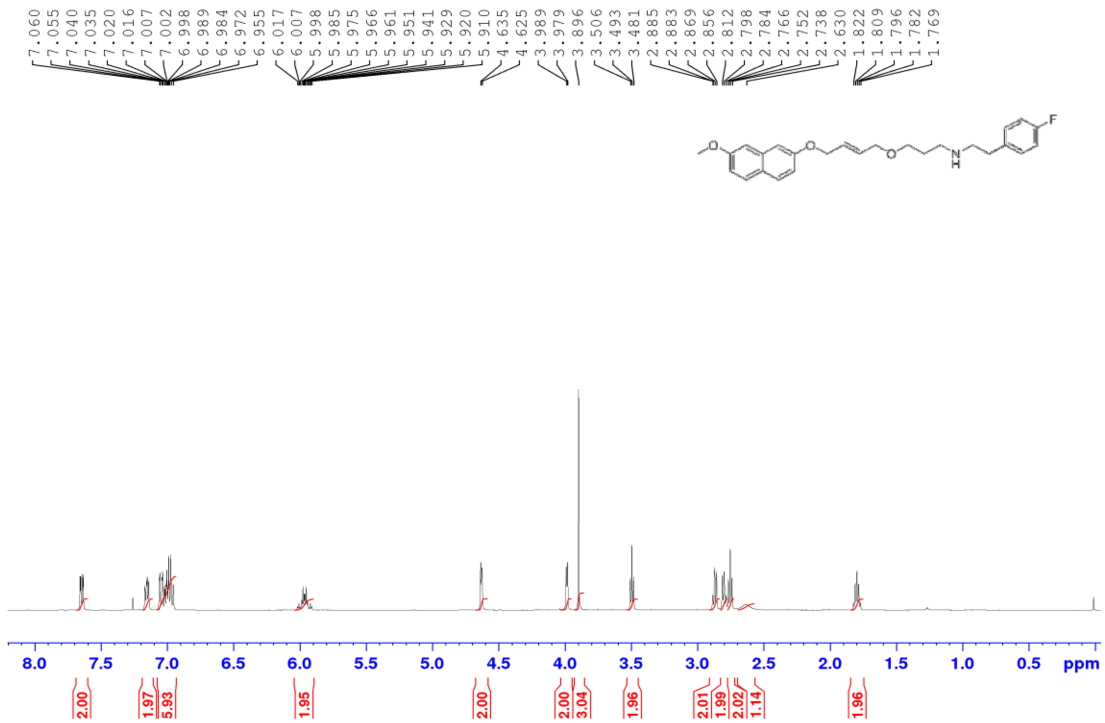
7.060
7.055
7.040
7.035
7.020
7.016
7.007
7.002
6.998
6.989
6.984
6.972
6.955
6.017
6.007
5.998
5.985
5.975
5.966
5.961
5.951
5.941
5.929
5.920
5.910
4.635
4.625
3.989
3.979
3.896
3.506
3.493
3.481
2.885
2.883
2.869
2.856
2.812
2.798
2.784
2.766
2.752
2.738
2.630
1.822
1.809
1.796
1.782
1.769
2.00
1.97
5.93
1.95
2.00
2.00
3.04
1.96
2.01
1.99
2.02
1.14
1.96
ppm

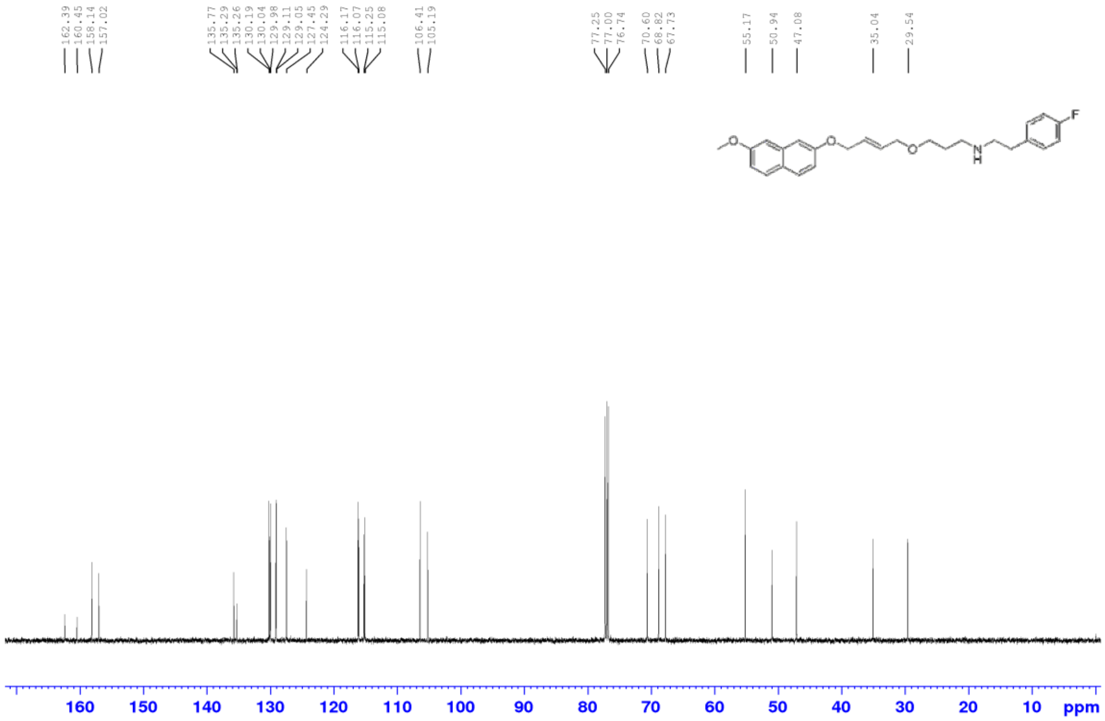
162.39
160.45
158.14
157.02
135.77
135.29
135.26
130.19
130.04
129.98
129.11
129.05
127.45
124.29
116.17
116.07
115.25
115.08
106.41
105.19
77.25
77.00
76.74
70.60
68.82
67.73
55.17
50.94
47.08
35.04
29.54
ppm

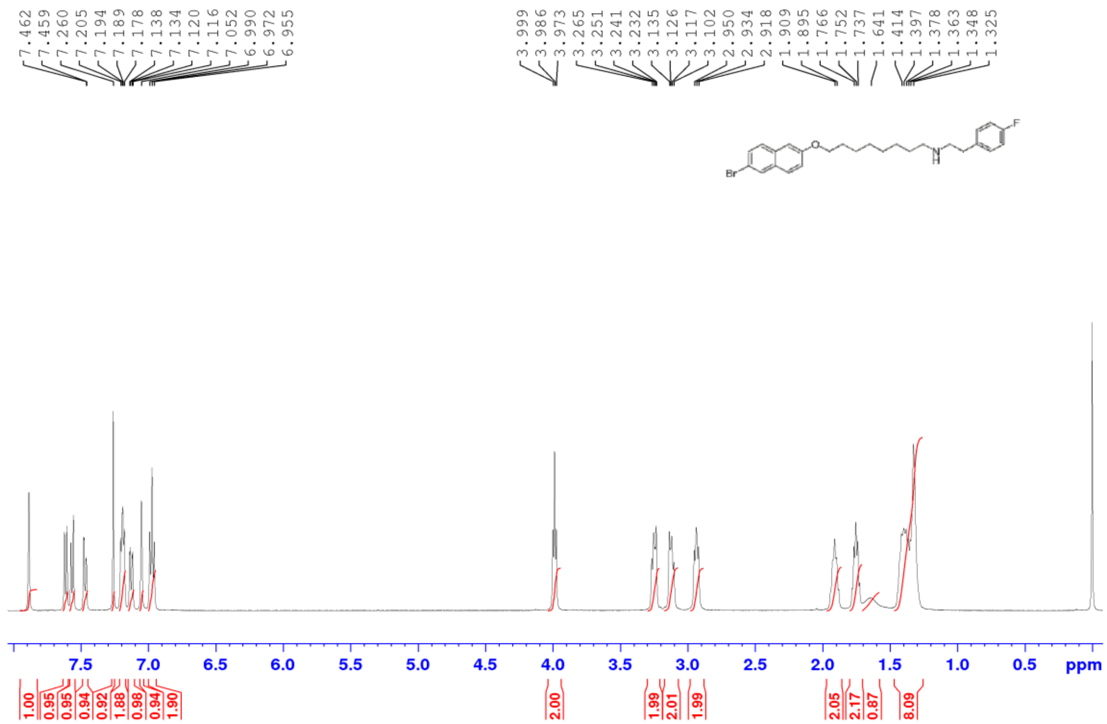

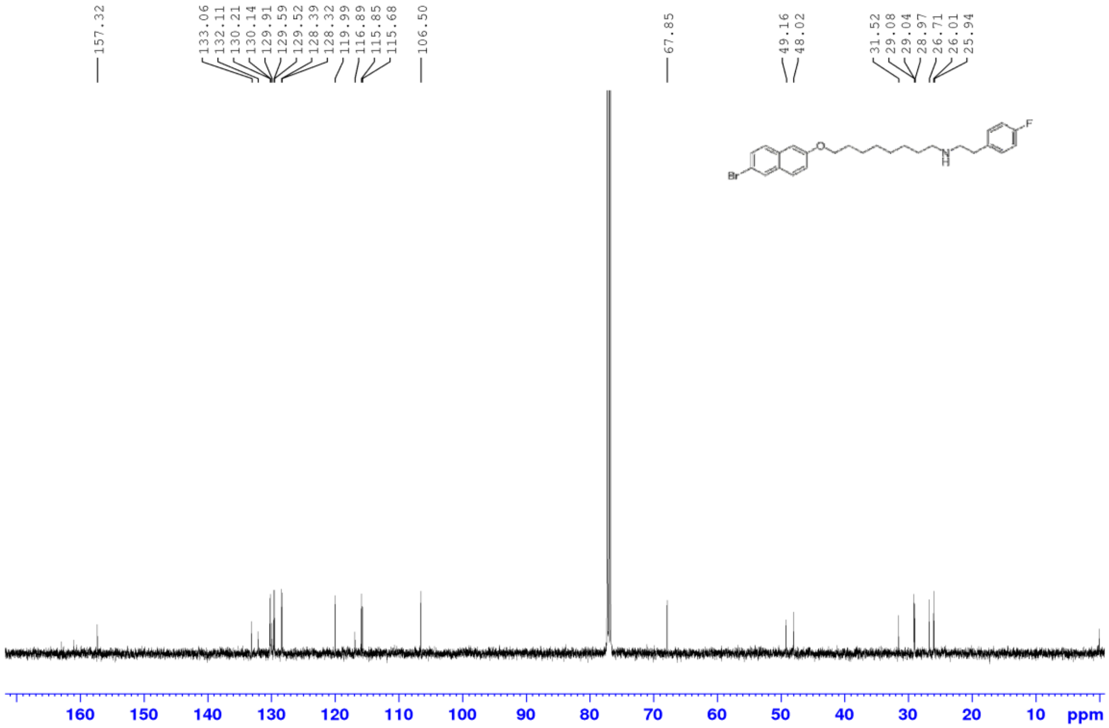

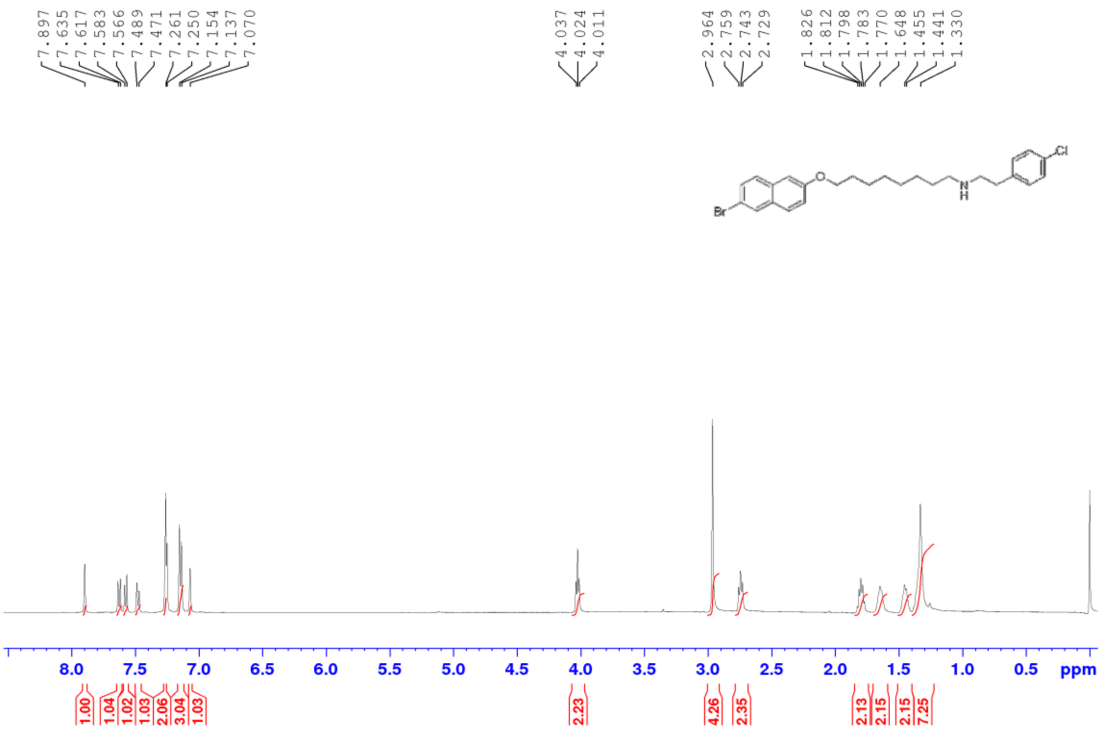

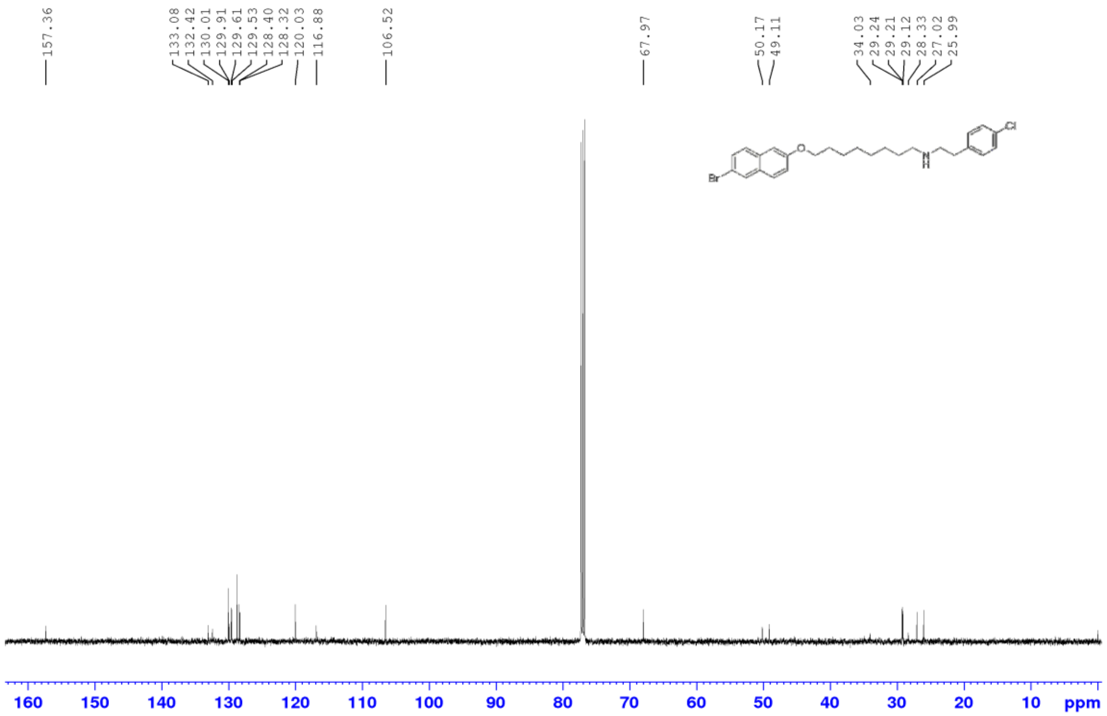

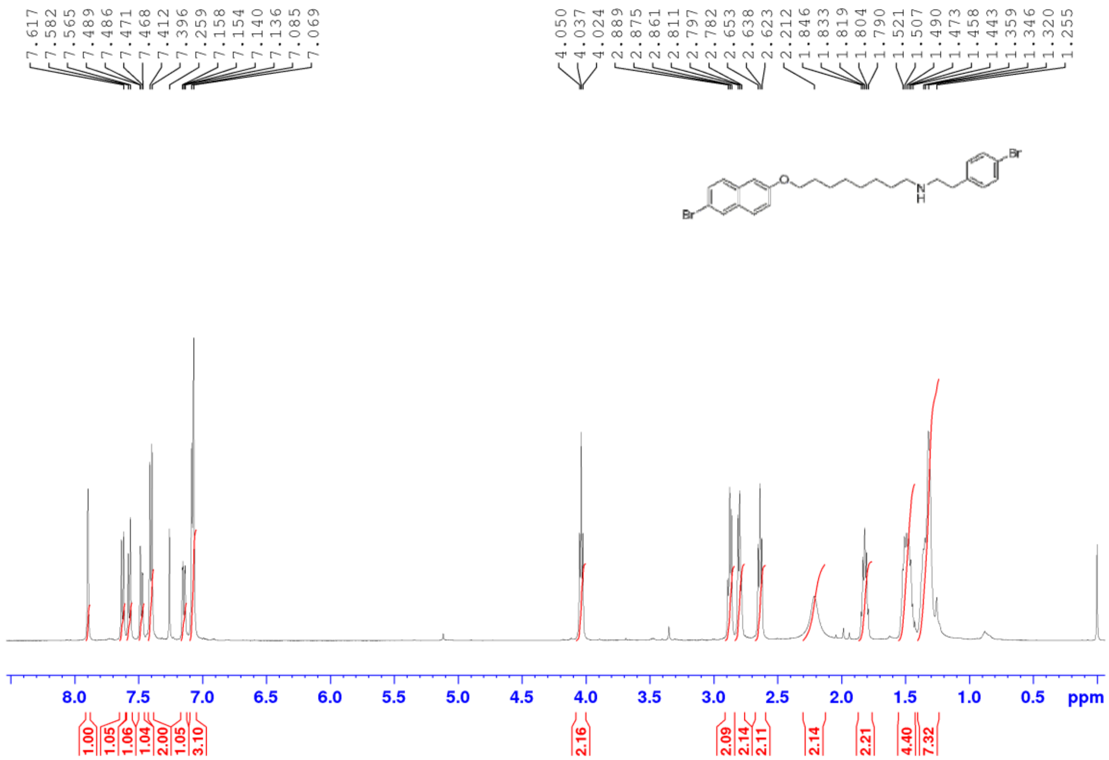

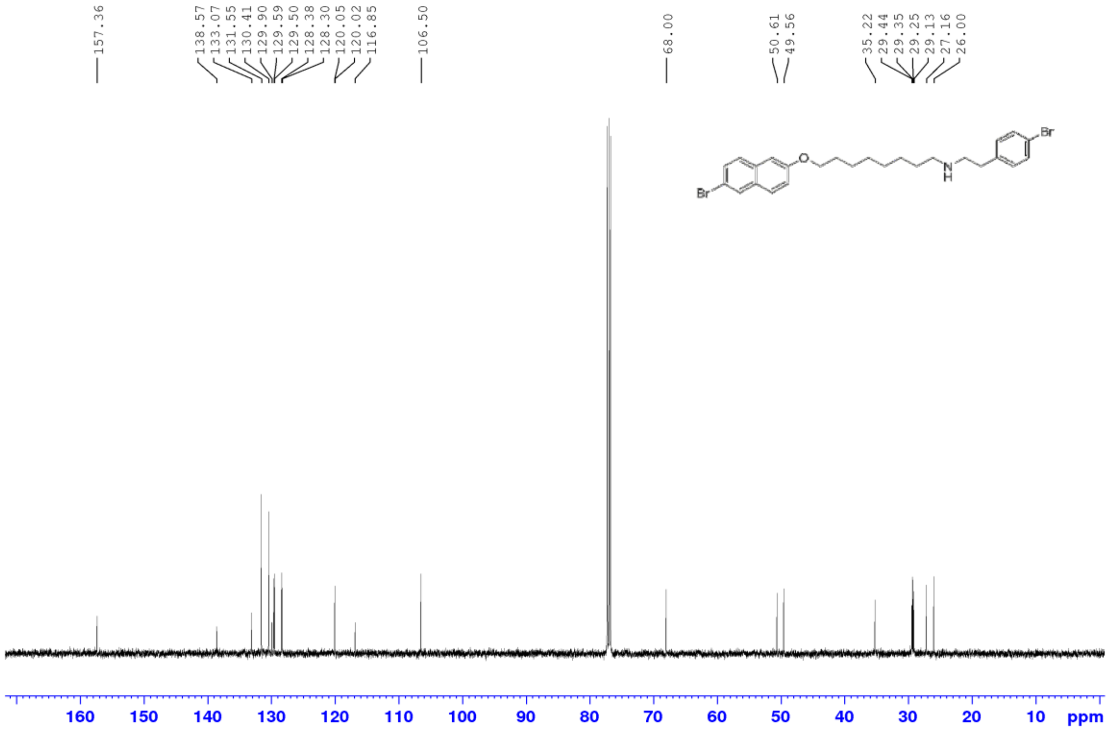

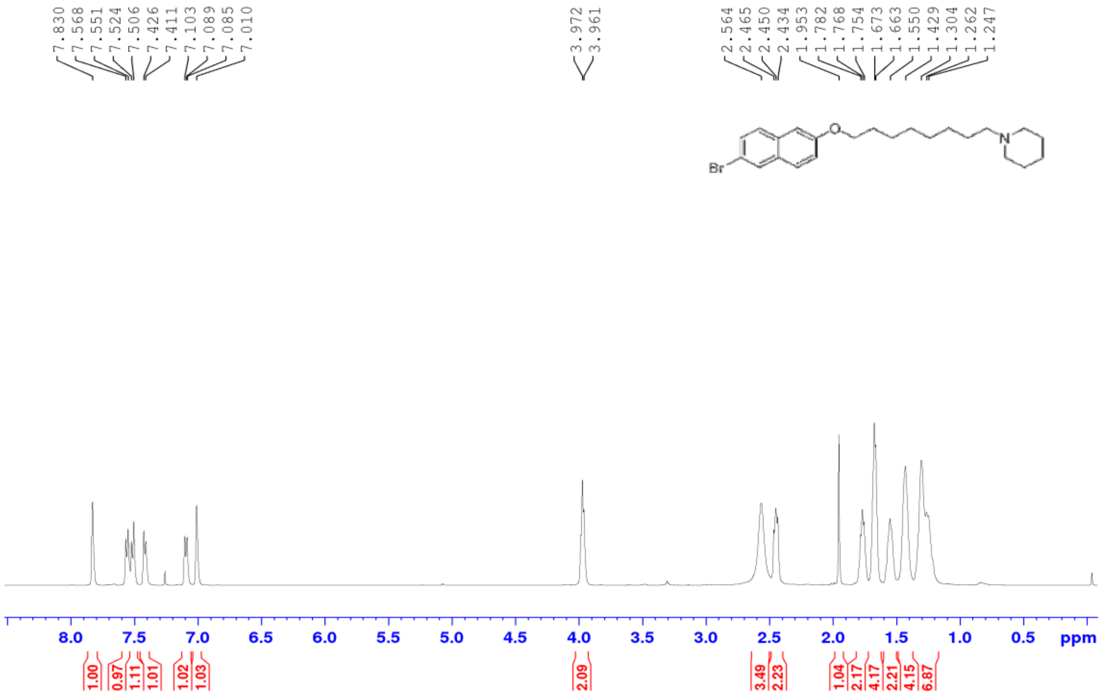
7.830
7.568
7.551
7.524
7.506
7.426
7.411
7.103
7.089
7.085
7.010
3.972
3.961
2.564
2.465
2.450
2.434
1.953
1.782
1.768
1.754
1.673
1.663
1.550
1.429
1.304
1.262
1.247
O
N
Br
8.0 7.5 7.0 6.5 6.0 5.5 5.0 4.5 4.0 3.5 3.0 2.5 2.0 1.5 1.0 0.5 ppm
1.00
0.97
1.11
1.01
1.02
1.03
2.09
3.49
2.23
1.04
2.17
4.17
2.21
4.15
6.87

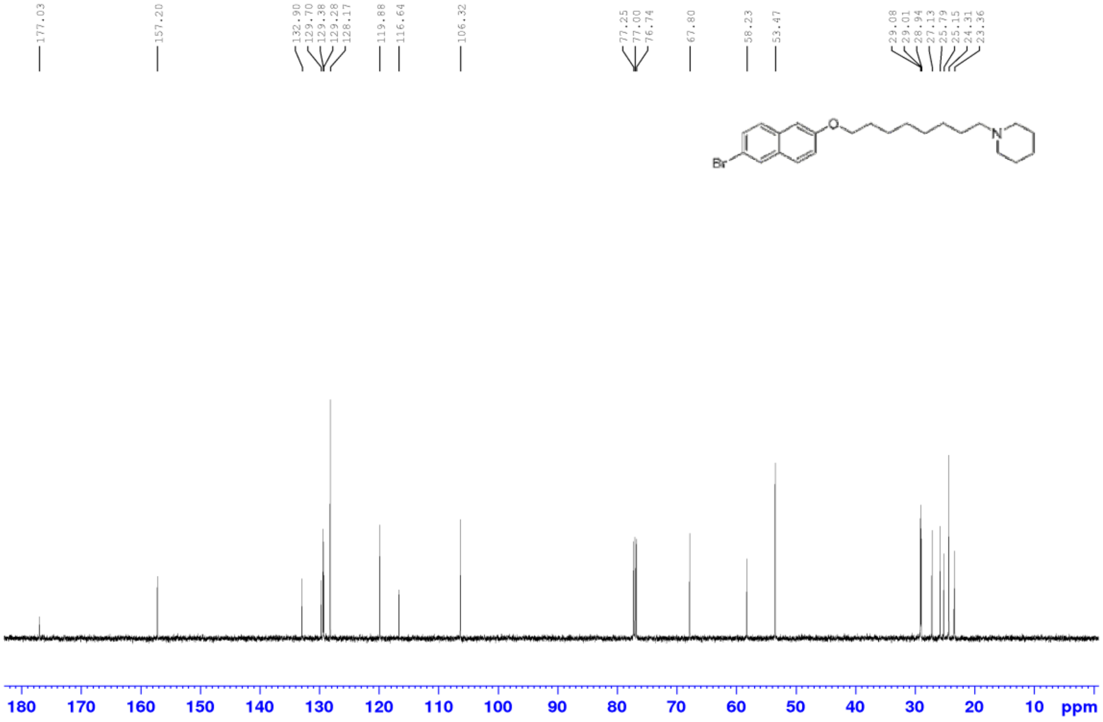
177.03
157.20
132.90
129.70
129.38
129.28
128.17
119.88
116.64
106.32
77.25
77.00
76.74
67.80
58.23
53.47
29.08
29.01
28.94
27.13
25.79
25.15
24.31
23.36
O
N
Br
180 170 160 150 140 130 120 110 100 90 80 70 60 50 40 30 20 10 ppm

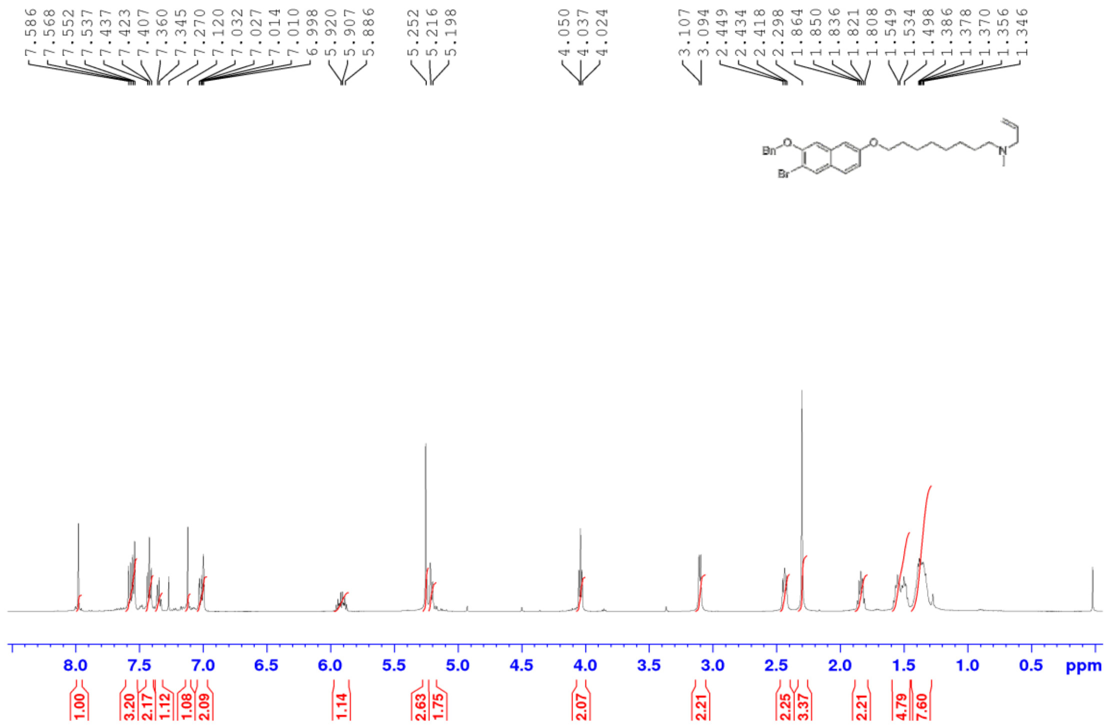

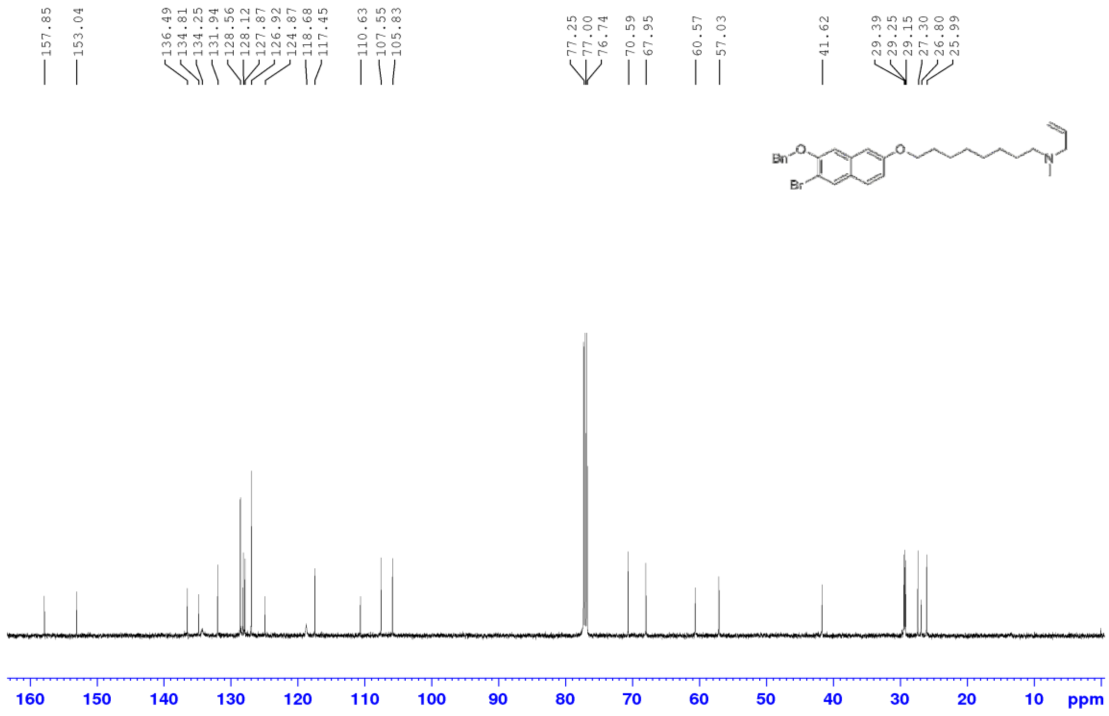

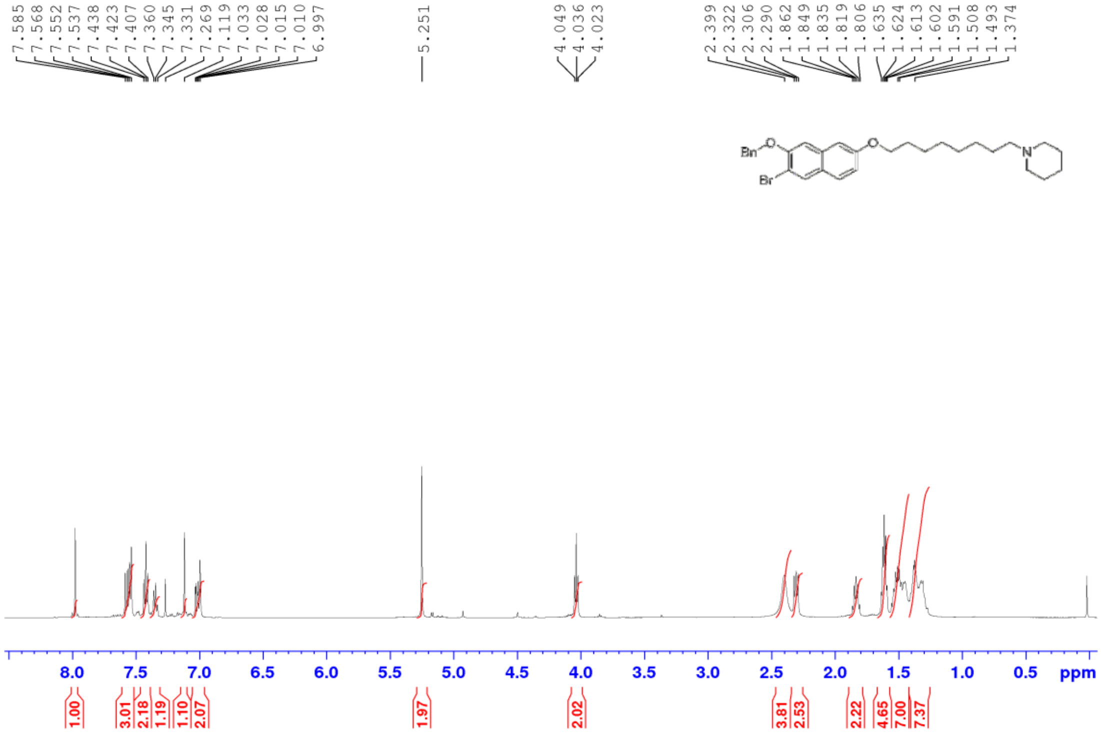
7.585
7.568
7.552
7.537
7.438
7.423
7.407
7.360
7.345
7.331
7.269
7.119
7.033
7.028
7.015
7.010
6.997
5.251
4.049
4.036
4.023
2.399
2.322
2.306
2.290
1.862
1.849
1.835
1.819
1.806
1.635
1.624
1.613
1.602
1.591
1.508
1.493
1.374
Bn
O
O
N
Br
8.0
7.5
7.0
6.5
6.0
5.5
5.0
4.5
4.0
3.5
3.0
2.5
2.0
1.5
1.0
0.5
ppm
1.00
3.01
2.18
1.19
1.10
2.07
1.97
2.02
3.81
2.53
2.22
4.65
7.00
7.37

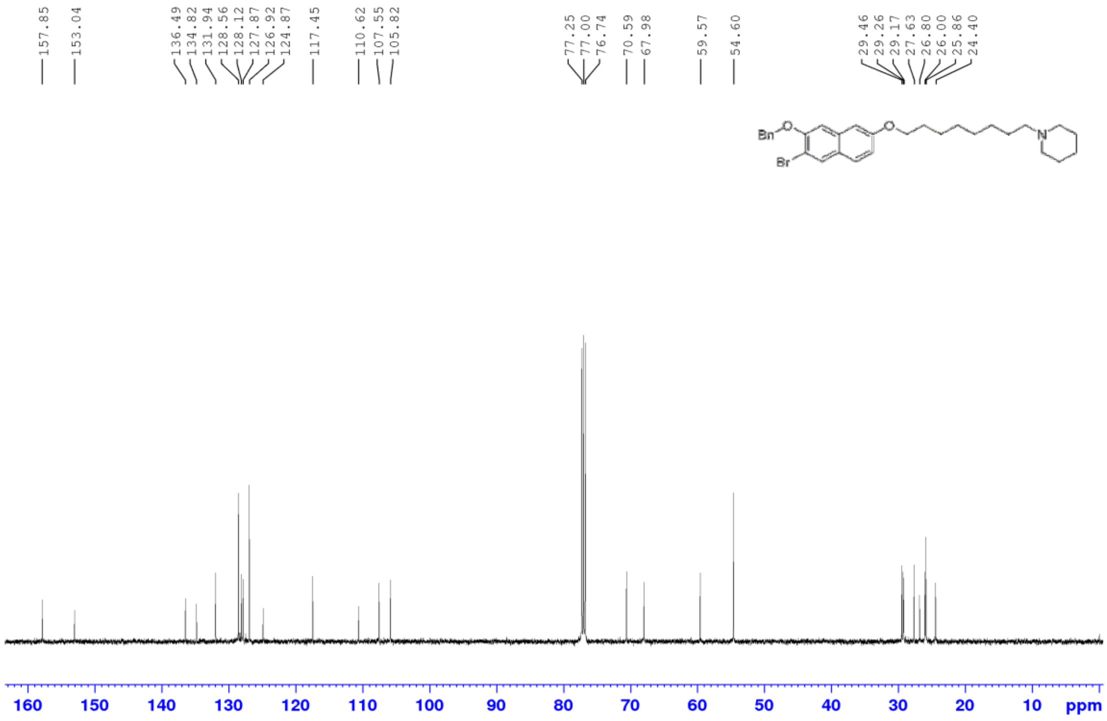
157.85
153.04
136.49
134.82
131.94
128.56
128.12
127.87
126.92
124.87
117.45
110.62
107.55
105.82
77.25
77.00
76.74
70.59
67.98
59.57
54.60
29.46
29.26
29.17
27.63
26.80
26.00
25.86
24.40
Bn
O
O
N
Br
160
150
140
130
120
110
100
90
80
70
60
50
40
30
20
10
ppm

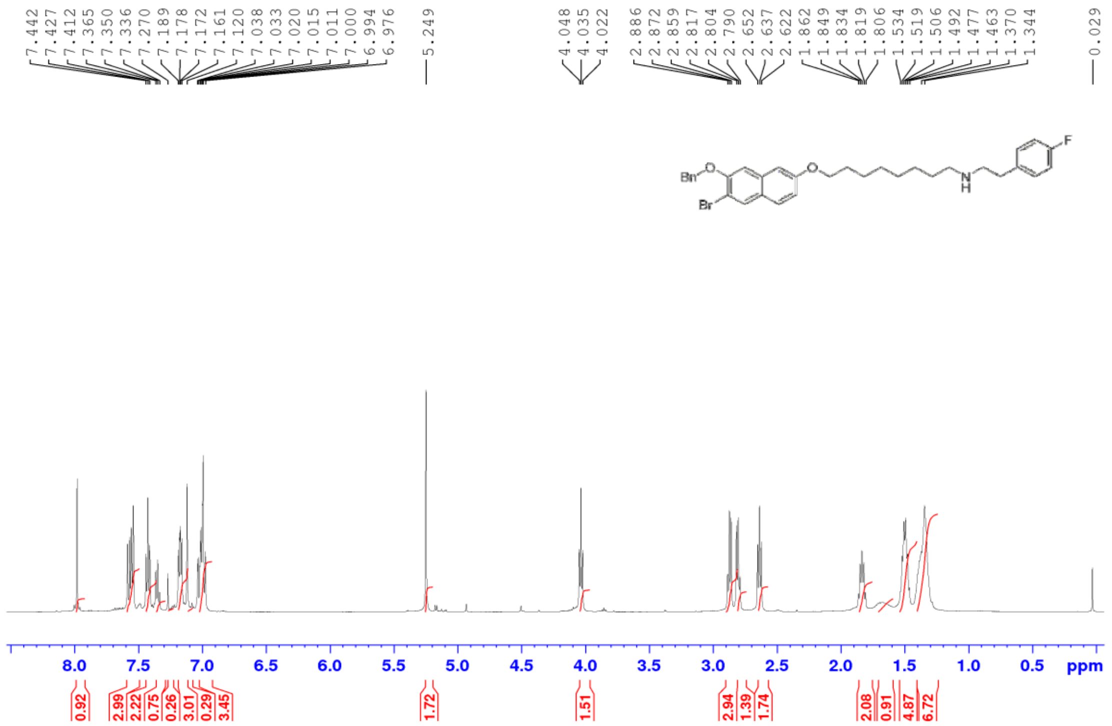

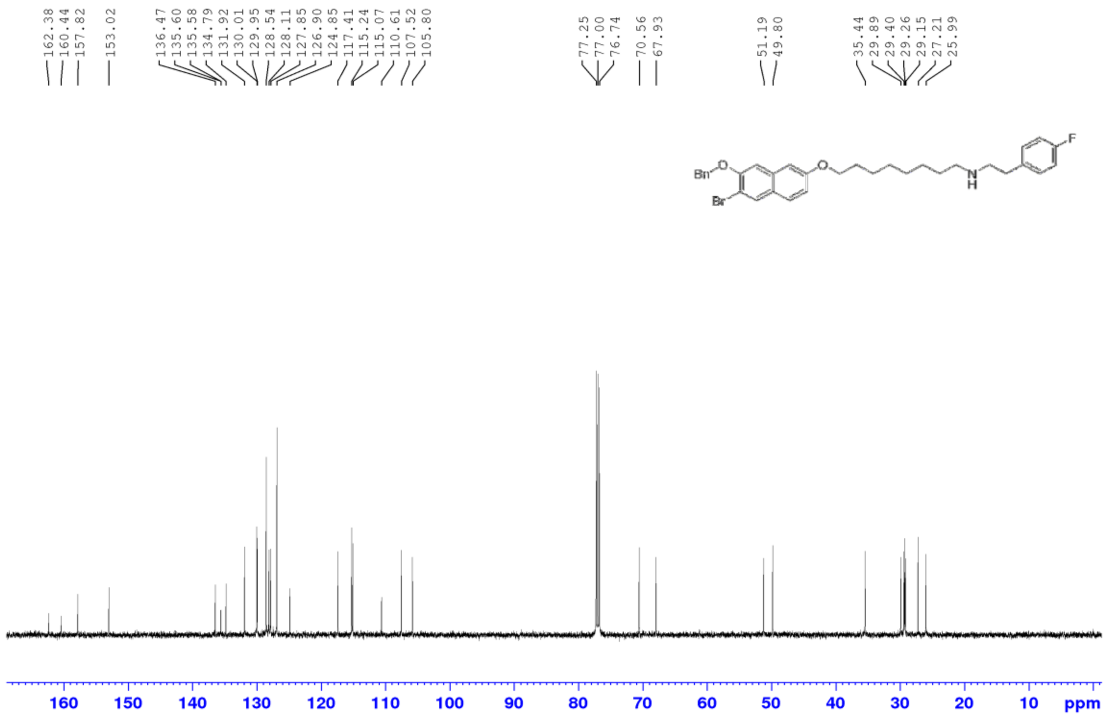

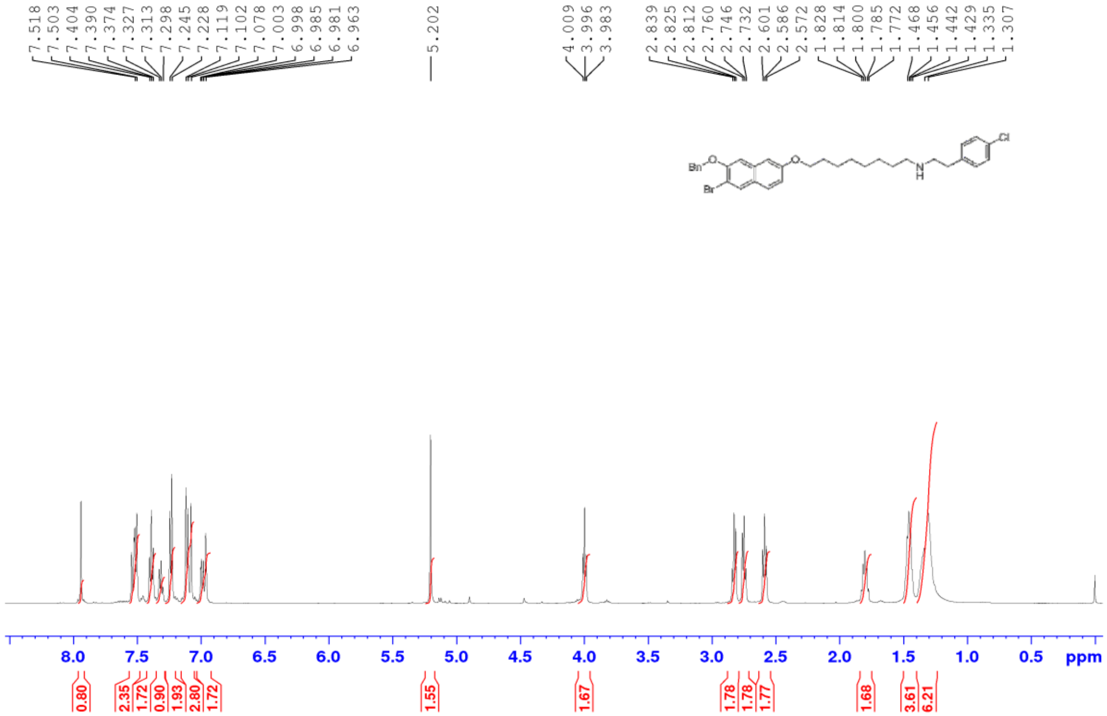

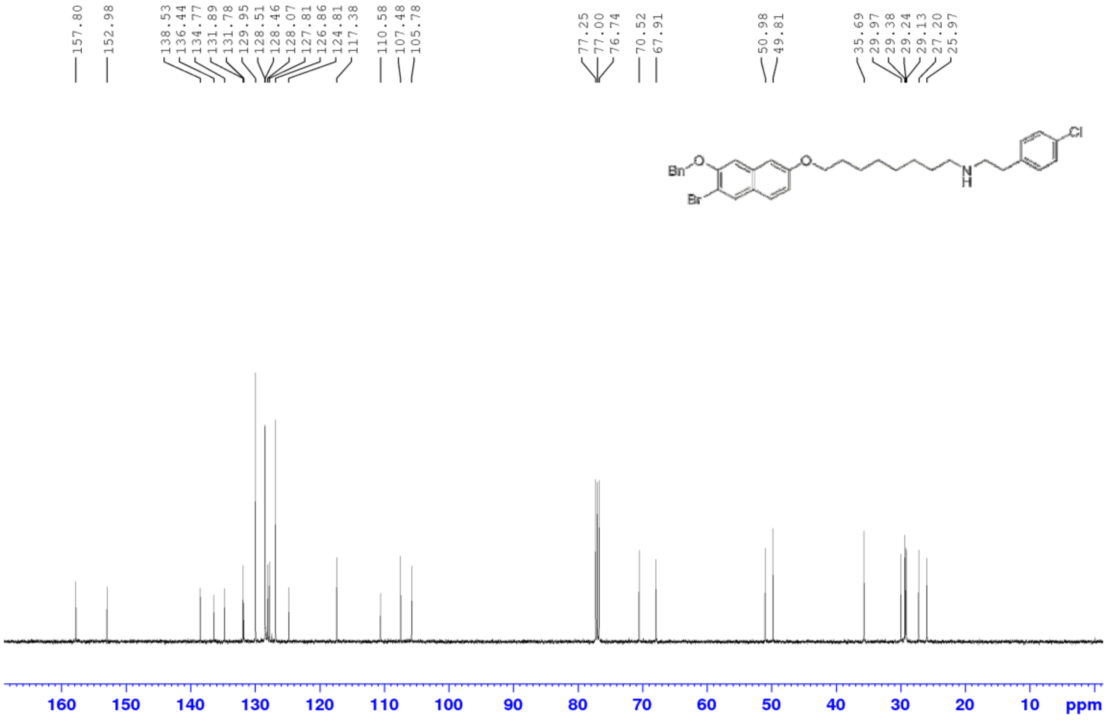

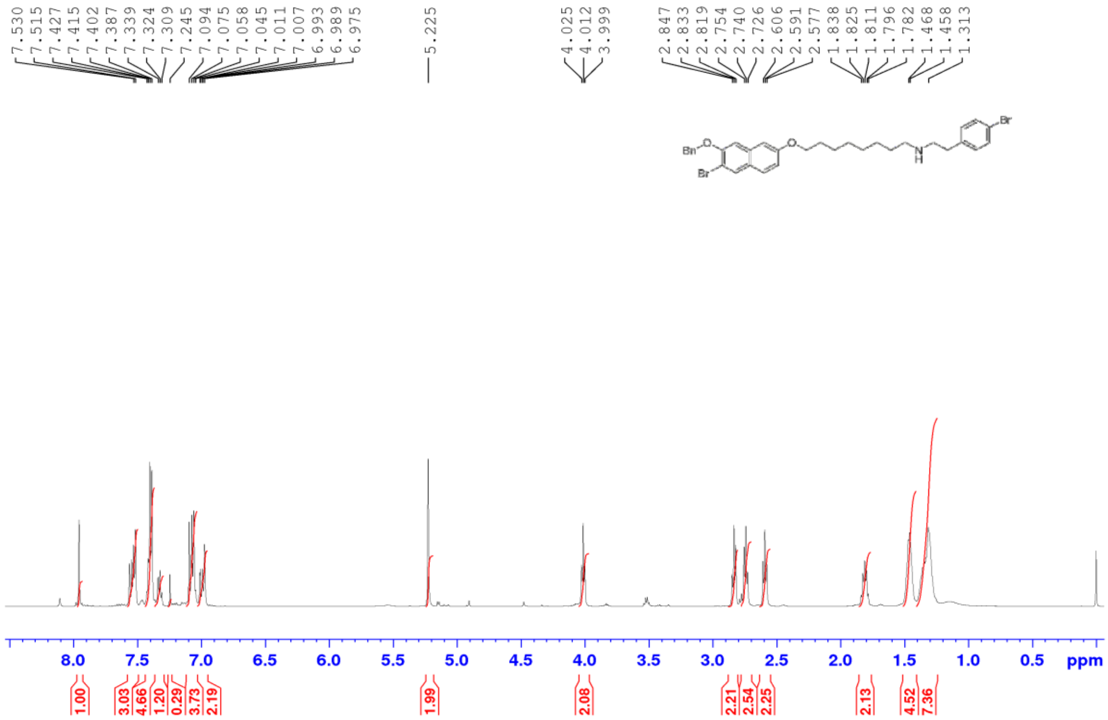
7.530 7.515 7.427 7.415 7.402 7.387 7.339 7.324 7.309 7.245 7.094 7.075 7.058 7.045 7.011 7.007 6.993 6.989 6.975
5.225
4.025 4.012 3.999
2.847 2.833 2.819 2.754 2.740 2.726 2.606 2.591 2.577 1.838 1.825 1.811 1.796 1.782 1.468 1.458 1.313
Bn
O
Br
O
N
H
Br
8.0 7.5 7.0 6.5 6.0 5.5 5.0 4.5 4.0 3.5 3.0 2.5 2.0 1.5 1.0 0.5 ppm
1.00 3.03 4.66 1.20 0.29 3.73 2.19 1.99 2.08 2.21 2.54 2.25 2.13 4.52 7.36

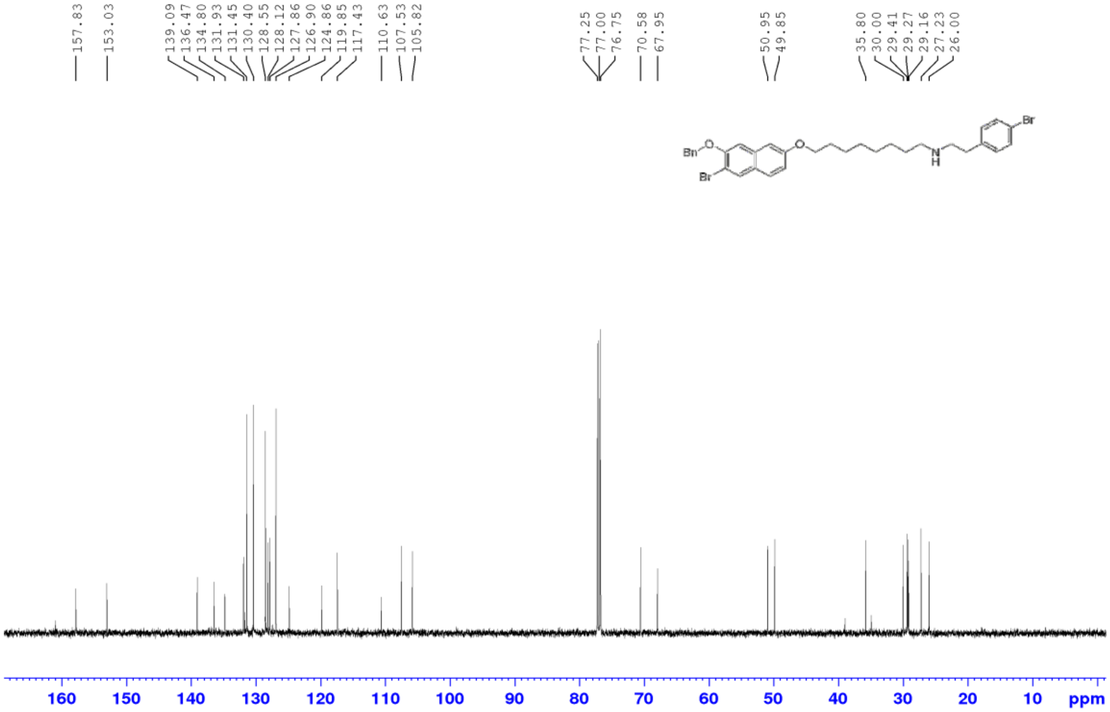
157.83
153.03
139.09 136.47 134.80 131.93 131.45 130.40 128.55 128.12 127.86 126.90 124.86 119.85 117.43
110.63 107.53 105.82
77.25 77.00 76.75
70.58 67.95
50.95 49.85
35.80 30.00 29.41 29.27 29.16 27.23 26.00
Bn
O
Br
O
N
H
Br
160 150 140 130 120 110 100 90 80 70 60 50 40 30 20 10 ppm

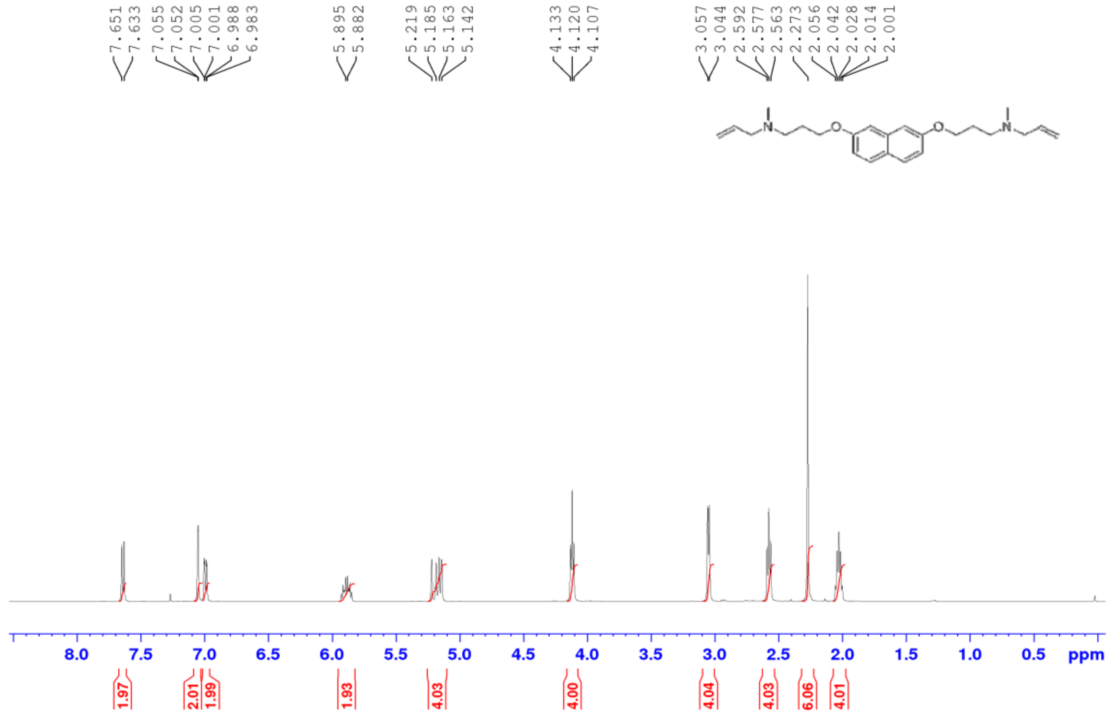
7.651
7.633
7.055
7.052
7.005
7.001
6.988
6.983
5.895
5.882
5.219
5.185
5.163
5.142
4.133
4.120
4.107
3.057
3.044
2.592
2.577
2.563
2.273
2.056
2.042
2.028
2.014
2.001
N
O
O
N
8.0
7.5
7.0
6.5
6.0
5.5
5.0
4.5
4.0
3.5
3.0
2.5
2.0
1.5
1.0
0.5
ppm
1.97
2.01
1.99
1.93
4.03
4.00
4.04
4.03
6.06
4.01

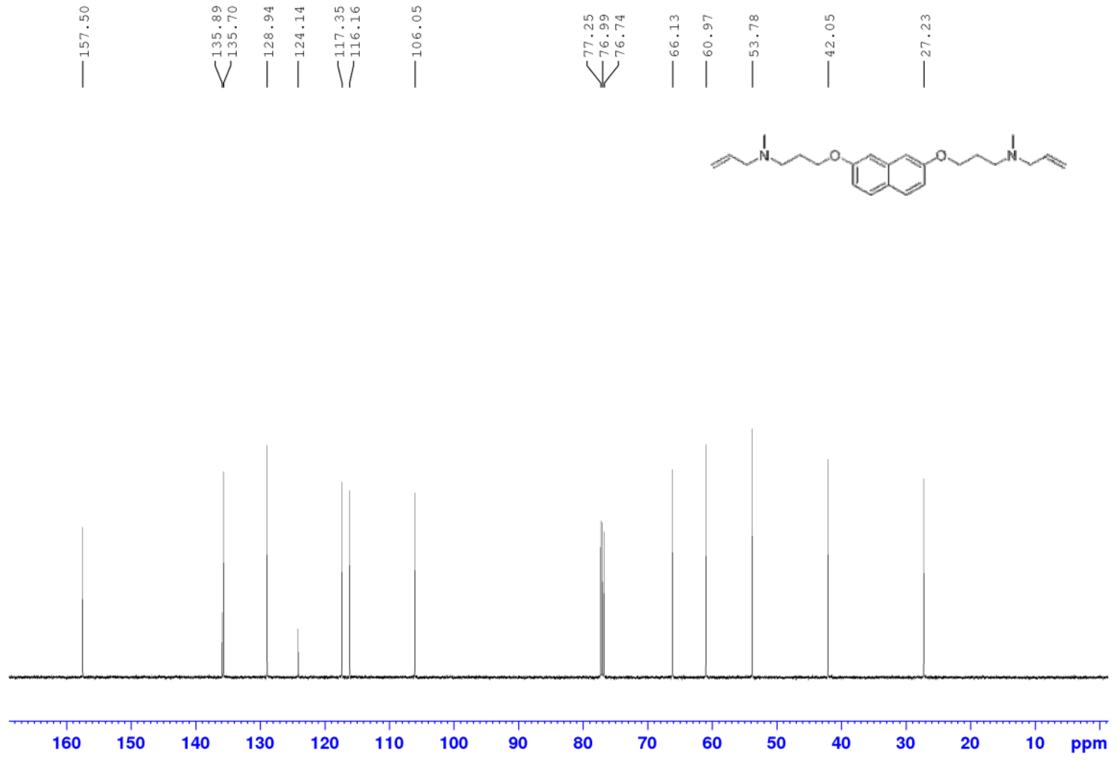
157.50
135.89
135.70
128.94
124.14
117.35
116.16
106.05
77.25
76.99
76.74
66.13
60.97
53.78
42.05
27.23
N
O
O
N
160
150
140
130
120
110
100
90
80
70
60
50
40
30
20
10
ppm

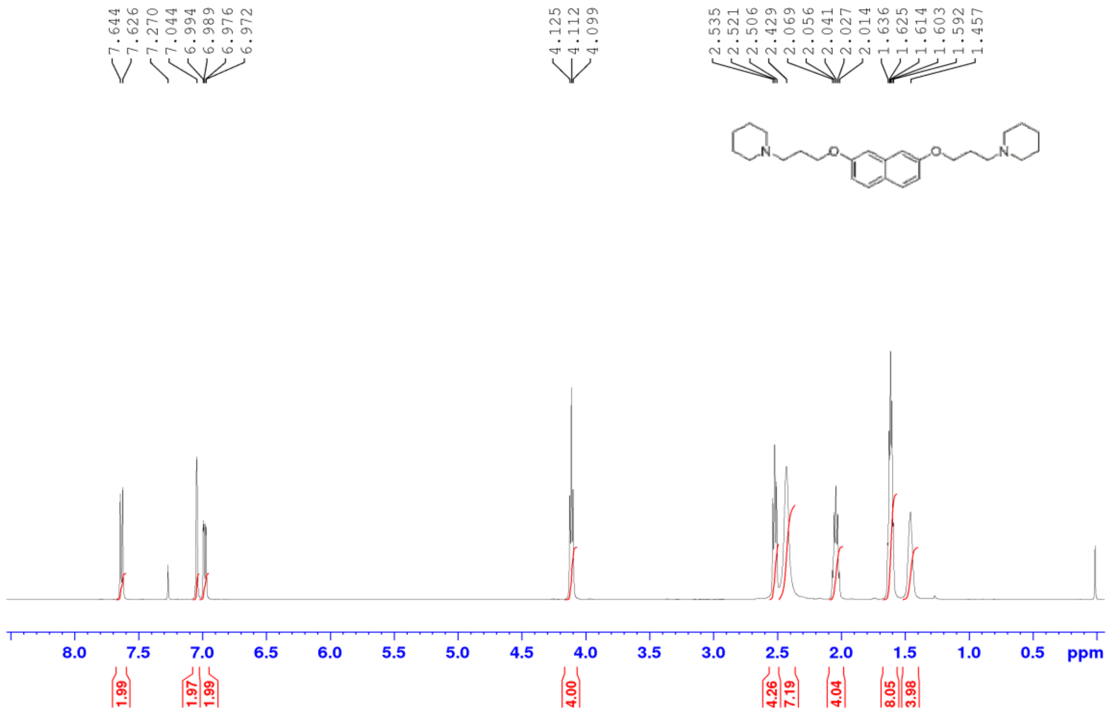
7.644
7.626
7.270
7.044
6.994
6.989
6.976
6.972
4.125
4.112
4.099
2.535
2.521
2.506
2.429
2.069
2.056
2.041
2.027
2.014
1.636
1.625
1.614
1.603
1.592
1.457
8.0 7.5 7.0 6.5 6.0 5.5 5.0 4.5 4.0 3.5 3.0 2.5 2.0 1.5 1.0 0.5 ppm
1.99
1.97
1.99
4.00
4.26
7.19
4.04
8.05
3.98

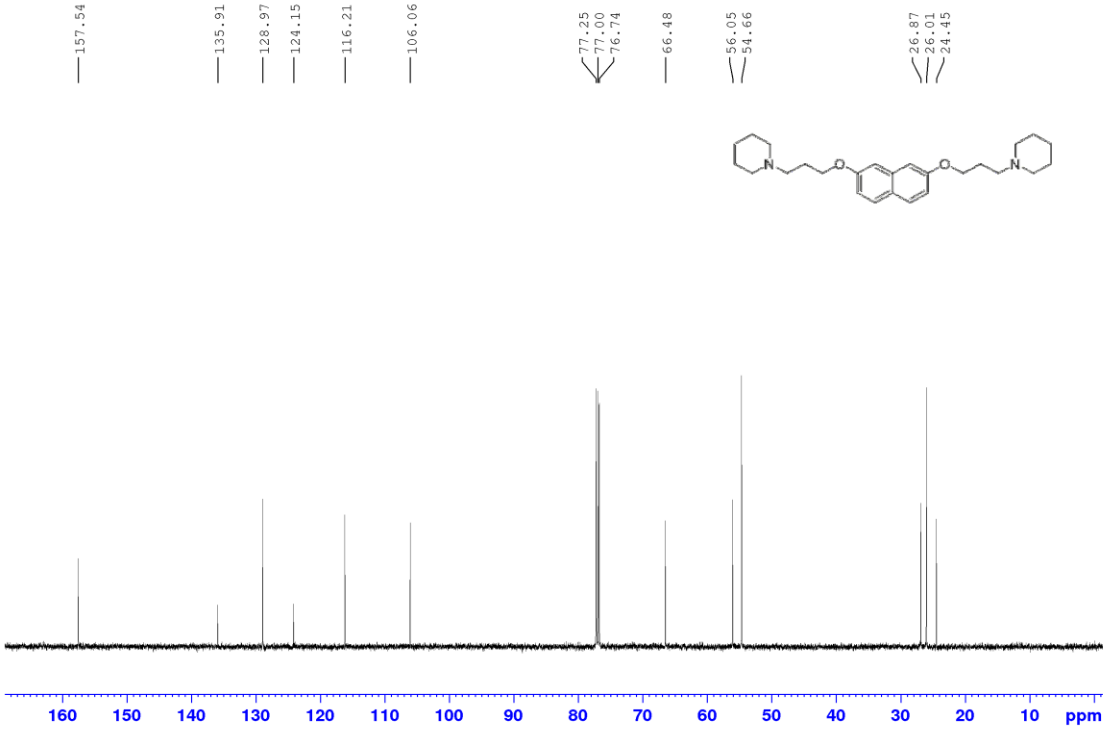
157.54
135.91
128.97
124.15
116.21
106.06
77.25
77.00
76.74
66.48
56.05
54.66
26.87
26.01
24.45
160 150 140 130 120 110 100 90 80 70 60 50 40 30 20 10 ppm

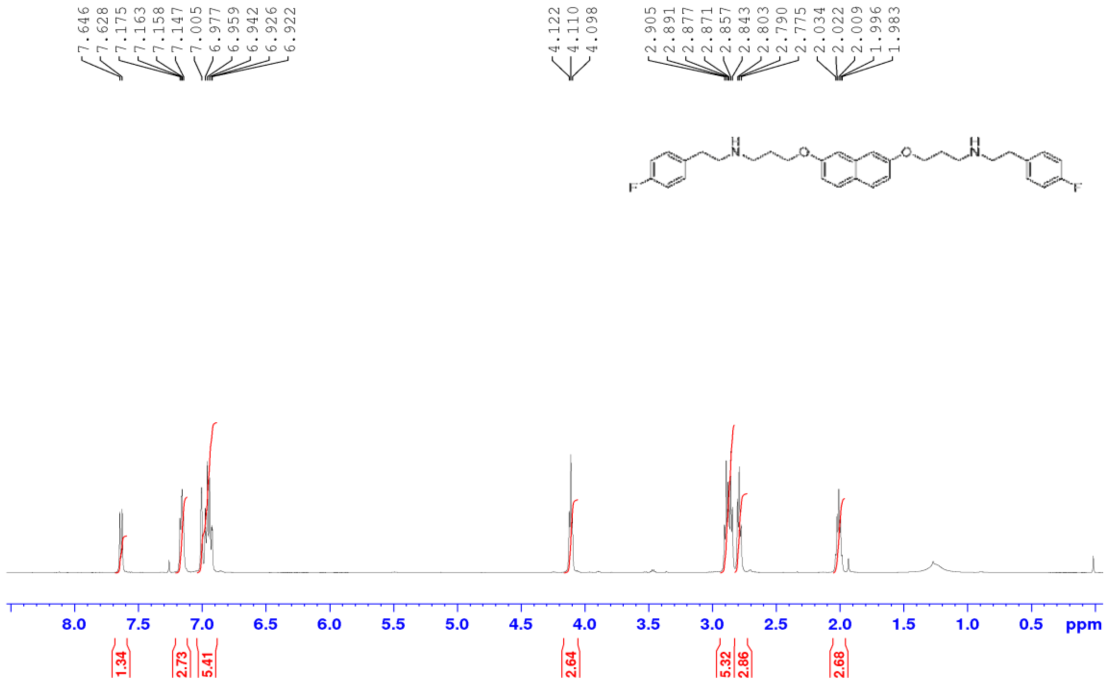
7.646 7.628 7.175 7.163 7.158 7.147 7.005 6.977 6.959 6.942 6.926 6.922
4.122 4.110 4.098
2.905 2.891 2.877 2.871 2.857 2.843 2.803 2.790 2.775 2.034 2.022 2.009 1.996 1.983
8.0 7.5 7.0 6.5 6.0 5.5 5.0 4.5 4.0 3.5 3.0 2.5 2.0 1.5 1.0 0.5 ppm
1.34 2.73 5.41 2.64 5.32 2.86 2.68

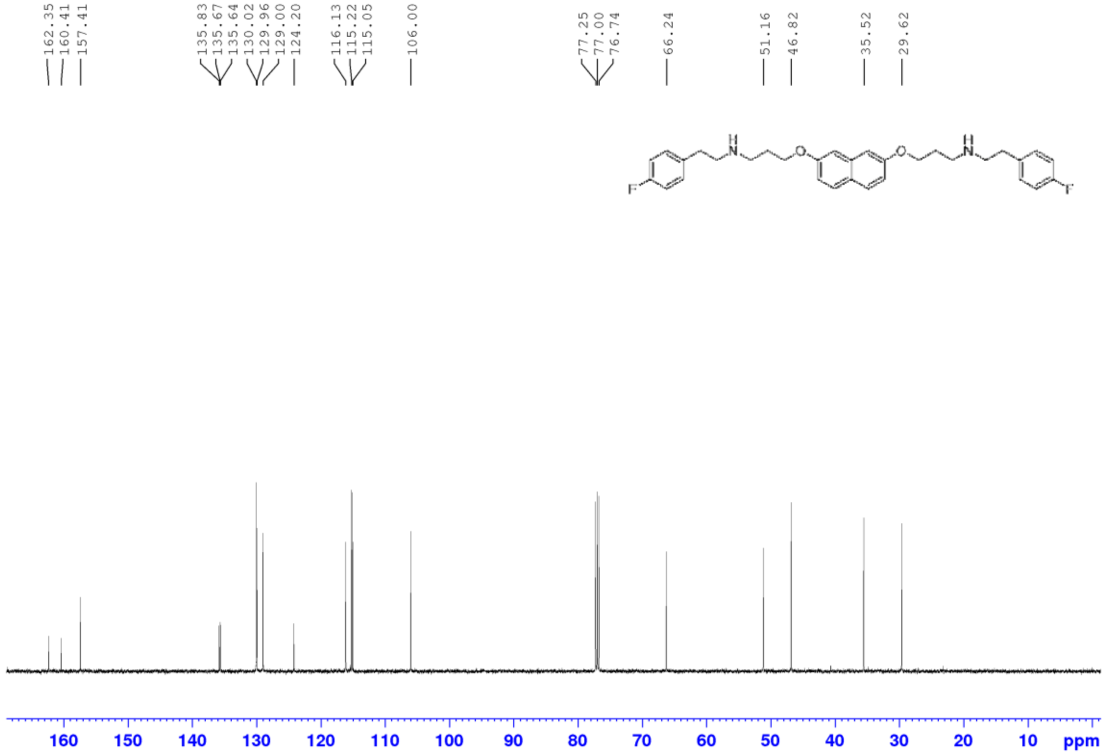
162.35 160.41 157.41
135.83 135.67 135.64 130.02 129.96 129.00 124.20
116.13 115.22 115.05
106.00
77.25 77.00 76.74
66.24
51.16 46.82
35.52 29.62
160 150 140 130 120 110 100 90 80 70 60 50 40 30 20 10 ppm

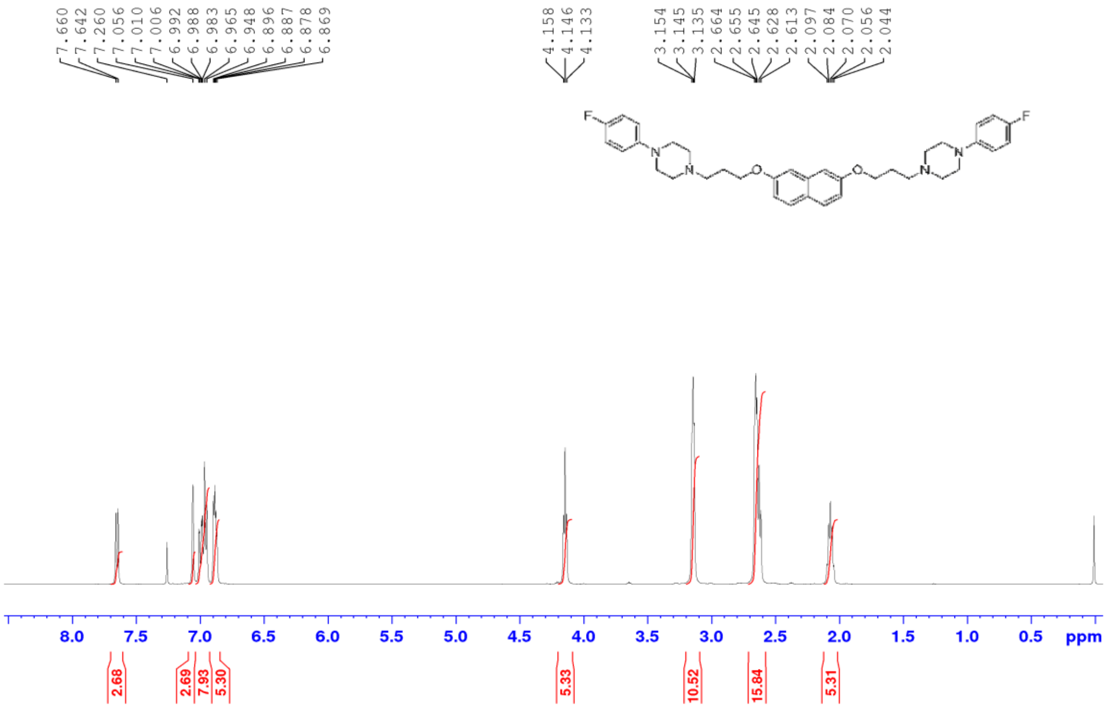
7.660
7.642
7.260
7.056
7.010
7.006
6.992
6.988
6.983
6.965
6.948
6.896
6.887
6.878
6.869
4.158
4.146
4.133
3.154
3.145
3.135
2.664
2.655
2.645
2.628
2.613
2.097
2.084
2.070
2.056
2.044
F
N
N
O
O
N
N
F
8.0
7.5
7.0
6.5
6.0
5.5
5.0
4.5
4.0
3.5
3.0
2.5
2.0
1.5
1.0
0.5
ppm
2.68
2.69
7.93
5.30
5.33
10.52
15.84
5.31

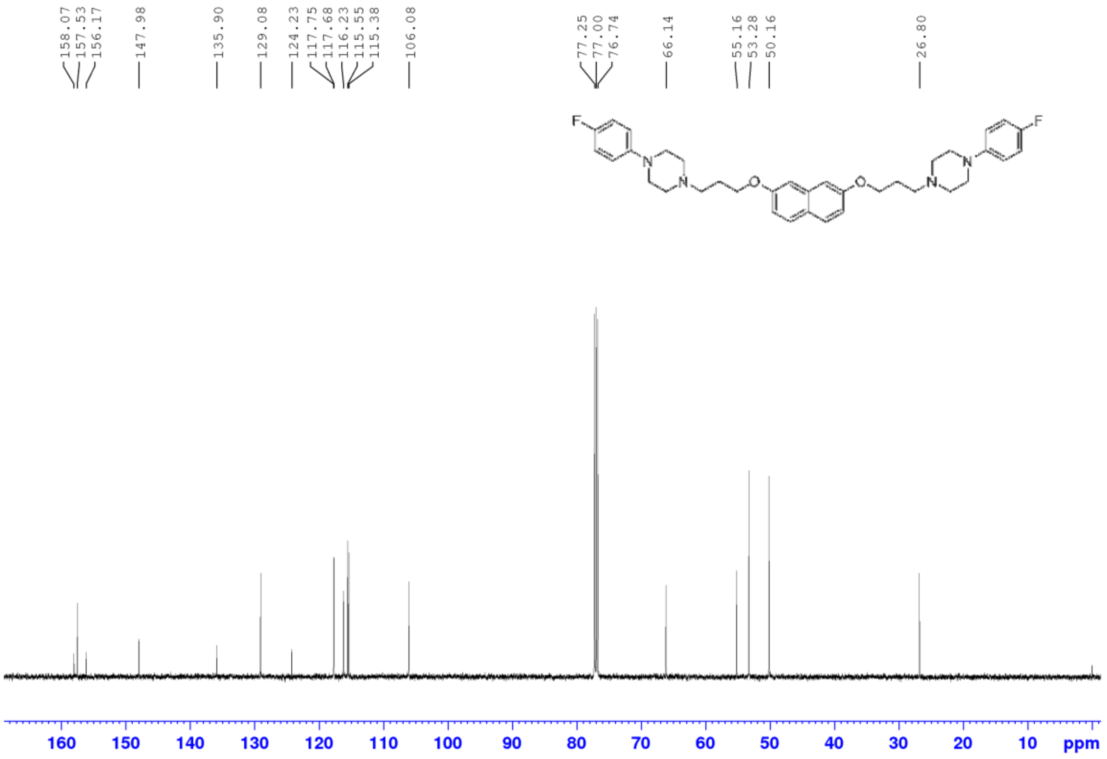
158.07
157.53
156.17
147.98
135.90
129.08
124.23
117.75
117.68
116.23
115.55
115.38
106.08
77.25
77.00
76.74
66.14
55.16
53.28
50.16
26.80
F
N
N
O
O
N
N
F
160
150
140
130
120
110
100
90
80
70
60
50
40
30
20
10
ppm
